# Genetic Diversity, Nitrogen Fixation, and Water Use Efficiency in a Panel of Honduran Common Bean (*Phaseolus vulgaris* L.) Landraces and Modern Genotypes

**DOI:** 10.3390/plants9091238

**Published:** 2020-09-19

**Authors:** Jennifer Wilker, Sally Humphries, Juan Carlos Rosas-Sotomayor, Marvin Gómez Cerna, Davoud Torkamaneh, Michelle Edwards, Alireza Navabi, K. Peter Pauls

**Affiliations:** 1 Department of Plant Agriculture, University of Guelph, Guelph, ON N1G 2W1, Canada; jwilker@uoguelph.ca (J.W.); dtorkama@uoguelph.ca (D.T.); edwardsm@uoguelph.ca (M.E.); 2 Department of Sociology and Anthropology, University of Guelph, Guelph, ON N1G 2W1, Canada; shumphri@uoguelph.ca; 3 Departamento de Ciencia y Producción Agropecuaria, Escuela Agrícola Panamericana, Zamorano, Tegucigalpa 11101, Honduras; jcrosas@zamorano.edu; 4 Fundación para la Investigación Participativa con Agricultores de Honduras, La Ceiba, Atlántida 561, Honduras; marvin.gomez@est.zamorano.edu

**Keywords:** nitrogen fixation, symbiosis, bean, landrace, PPB, participatory breeding, climate resilient, Honduras

## Abstract

Common bean (*Phaseolus vulgaris* L.) provides critical nutrition and a livelihood for millions of smallholder farmers worldwide. Beans engage in symbiotic nitrogen fixation (SNF) with Rhizobia. Honduran hillside farmers farm marginal land and utilize few production inputs; therefore, bean varieties with high SNF capacity and environmental resiliency would be of benefit to them. We explored the diversity for SNF, agronomic traits, and water use efficiency (WUE) among 70 Honduran landrace, participatory bred (PPB), and conventionally bred bean varieties (HON panel) and 6 North American check varieties in 3 low-N field trials in Ontario, Canada and Honduras. Genetic diversity was measured with a 6K single nucleotide polymorphism (SNP) array, and phenotyping for agronomic, SNF, and WUE traits was carried out. STRUCTURE analysis revealed two subpopulations with admixture between the subpopulations. Nucleotide diversity was greater in the landraces than the PPB varieties across the genome, and multiple genomic regions were identified where population genetic differentiation between the landraces and PPB varieties was evident. Significant differences were found between varieties and breeding categories for agronomic traits, SNF, and WUE. Landraces had above average SNF capacity, conventional varieties showed higher yields, and PPB varieties performed well for WUE. Varieties with the best SNF capacity could be used in further participatory breeding efforts.

## 1. Introduction

The common bean (*Phaseolus vulgaris* L.) is the most important food legume grown and consumed worldwide. High in protein, fiber, and essential nutrients, the nutritional profile and affordability of beans relative to other protein sources make beans a dietary staple in developing economies. 

A member of the Fabaceae family, common bean is a predominantly self-pollinating species with a genome size of 587 Mbp and ploidy of 2n = 2x = 22 [1]. The center of origin for common bean is in present-day Central Mexico [2]. As a result of geographical dispersion, the ancestral bean diverged and evolved into two domesticated gene pools. Larger-seeded market classes evolved and were domesticated in South America and belong to the Andean gene pool, while smaller-seeded market classes evolved and were domesticated in Central America and belong to the Middle American gene pool [2,3,4,5,6,7]. 

Beans are traditionally grown as monocrops or with maize in either a relay cropping or an intercropping system in Honduras. There are two growing seasons—the rainy *Primera* (May–September) and the traditionally dry *Postrera* (October–January)—although climate change causes more fluctuation in precipitation levels and the duration of these seasons. Food insecurity is an issue in hillside communities and is a particular problem during the summer months before the *Primera* harvest. In some locations, this hungry period is termed *los junios* after the month during which food becomes scarce. More than 50% of bean production in Honduras takes place on steep hillside slopes (15–30° and greater) [8]. In addition, the country’s infrastructure is poor, and less than 30% of the bean growing area is located within an hour’s travel to a road, thus restricting market access [8].

Bean production in Honduras is affected by various biotic and abiotic stresses, and productivity is low and averaged 785 kg ha^−1^ in 2018 compared to yields in Canada, which averaged 2888 kg ha^−1^ in 2018 [9]. Bean diseases and insect pests comprise the primary biotic stresses of Honduran bean production. The most impactful diseases are Bean Golden Mosaic Virus (BGMV), rust (caused by *Uromyces phaseoli*), web blight (caused by *Rhizoctonia solani*), anthracnose (caused by *Colletotrichum lindemuthianum*), and angular leaf spot (ALS; caused by *Pseudoscercospora griseola*) [8]. The whitefly (*Bemisia tabaci*) is the vector for BGMV and is the most important insect pest of bean in Honduras. Weevils (*Acanthoscelides obtectus* and *Zabrotes subfasciathus*) are serious pests of stored beans, reducing marketability and damaging seed for planting. Climate change is expected to affect the impact of these biotic stresses in bean production and may lead to a shift in the complex of pests and diseases involved [10].

Extreme weather, such as high temperatures and flooding, including from hurricanes, reduces bean production. Climate models for Honduras predict higher temperatures and reduced overall rainfall but more extreme weather events increasing floods in the coming decades [11]. Another abiotic stress impacting bean production is soil health. Soils across Central America are deficient in available phosphorus (P), nitrogen (N), calcium (Ca), and organic matter, and aluminum (Al) and manganese (Mn) toxicity are exacerbated by low soil pH levels. Bean productivity is limited by soil nutrient availability, particularly N and P [12]. Nitrogen deficiency reduces grain yield because N is a structural component of various essential molecules, including chlorophyll, amino acids, nucleic acids, and lipids, required for the production of storage carbohydrates and proteins. Soils can be supplemented with nitrogen fertilizer throughout the growing season to avert yield losses; however, synthetic amendments are expensive, difficult to access, and generally not used by bean growers in Honduras. Instead, bean growers rely on organic forms of N, including that derived through symbiotic nitrogen fixation (SNF), as a nutrient source for their crop.

Beans are capable of generating their own organic nitrogen through SNF where nitrogen-fixing bacteria infect root nodules and reduce atmospheric nitrogen into forms useable by the host plant in exchange for carbohydrates [13]. SNF is a complex biological process and its efficiency is impacted by abiotic, biotic, and genetic factors, including soil nutrient levels, environmental conditions, the presence of efficient *Rhizobium* strains, and genetic constitution of the crop grown [12]. Recent studies have confirmed that SNF capacity in beans has a wide range and can reach high, yield-sustaining levels under optimal conditions [14,15,16,17,18,19,20,21,22]. For example, Kamfwa et al. [14] reported a range of 3.6 to 98.2% nitrogen derived from the atmosphere, and Aserse et al. [20] found that inoculated beans had comparable yields to those grown with nitrogen fertilizer.

Smallholder hillside farmers (0.5–5 ha) comprise approximately 70% of Honduran bean growers, and the remainder of production occurs in foothill and valley regions by larger-scale producers. Hillside farmers cultivate marginal land with steep slopes and low soil fertility, they tend towards subsistence production, and produce primarily for household consumption following traditional practices and planting traditional crop varieties. These smallholder farmers have limited access to markets, which has a two-fold impact, reducing the influence of market demands on what growers produce and limiting access to modern bean varieties and production inputs. These constraints notwithstanding, hillside farmers market approximately 50% of their bean harvest.

Landraces, known locally as *criollos*, comprise the majority of varieties traditionally grown by hillside farmers in Honduras [23]. Ninety-five percent of beans produced across the country are small, light red beans, which are preferred by Hondurans [8], and are also exported to El Salvador and to the United States to meet the needs of Central Americans who have emigrated there. Some black beans are grown in Honduras and are primarily exported to neighboring Guatemala where that market class is favored [8]. Landraces have local genetic adaptation, high genetic diversity, and lack formal genetic improvement [24]. The genetic heterogeneity of bean landraces lends resilience and makes them able to adapt to the changeable growing conditions of mountain hillsides and other marginal areas where they are grown. Among the preferred traits of the landraces included in this study are adaptation to cultivation at a range of altitudes, more marketable seed coat color and appealing kinesthetic properties, and yield stability in a changeable climate. In addition to traditional landraces, hillside bean farmers also grow conventionally bred and participatory bred varieties.

Conventional bean breeding in Honduras has been primarily the responsibility of the *Programa de Investigaciones en Frijol (PIF)* in the Department of Agronomy at the *Escuela Agricola Pan-Americana* (Zamorano) since the late 1980s when government funding to the agricultural research department (*Dirección de Ciencia y Tecnología Agropecuaria, DICTA*) was reduced restricting agricultural research and extension services [25]. The International Center for Tropical Agriculture (CIAT), the Bean/Cowpea Collaborative Research Support Program (CRSP), and the Regional Cooperative Bean Program (*Programa Cooperativo Regional de Frijol*-PROFRIJOL) have also been involved in variety development and/or providing funding for bean research in Honduras. Zamorano’s early breeding focus was on developing conventional varieties with BGMV resistance and improved heat and drought tolerance for the lowland and valley production regions of Honduras [25]. By the late 1990s, Zamorano took leadership in bean breeding for the region, developing small red varieties for Honduras, Guatemala, El Salvador, Nicaragua, and Costa Rica, as well as black bean varieties for Guatemala and Haiti [26]. Conventionally bred commercial varieties are adapted for cultivation across a wide geographic region and have disease resistance and agronomic traits, which can bolster their yields. Adoption of commercial varieties among hillside growers is limited for a number of reasons, including darker seed coat color and other culinary traits, which reduce their marketability, as well as poor yield performance compared to landraces [23]. In the mid 1990s, Zamorano embarked on collaborative research with social scientists from CIAT to explore the social and economic factors that impact adoption of conventional varieties [23]. As a result, participatory research has become an important method used in the Zamorano bean breeding program.

The term participatory plant breeding encompasses two main methods of plant variety development, ‘participatory varietal selection’ and ‘participatory plant breeding’. Participatory varietal selection (PVS) involves farmers locally testing varieties or advanced breeding lines provided by a formal plant breeder and making selections based on their needs [27]. Participatory plant breeding (PPB) involves farmers locally testing early stage (F_2_-F_3_) breeding material and can further involve the farmers actively participating in choosing parents and driving variety development by selecting progeny that meet local needs and preferences. As in other reports on the subject, the term PPB will be used to refer to both PPB and PVS in this study [28,29].

Participatory bean breeding at Zamorano has been facilitated through collaboration with CIAT-initiated *comités de investigación agrícola local* (CIALs), which are village-level farmer research teams that create a space where applied agricultural research can be carried out. For this study, we collaborated with the *Fundación para la Investigación Participativa con Agricultores de Honduras* (FIPAH) and *Programa de Reconstrucción Rural* (PRR). FIPAH supports over 100 CIALs, backstopped by regional offices across the country (https://fipah-hn.org/). PRR is an NGO that works with smallholder farmers in Santa Barbara and Lempira and supports approximately 60 CIALs (https://www.iaf.gov/grants/honduras/2017-prr/) [30]. CIAL members are trained in the scientific method, and most CIALs focus their research on obtaining higher-yielding and climate-resilient corn and bean varieties. The relationship between the CIALs and the bean breeding program at Zamorano is collegial and formal, responding to the needs of the farmers while the research is carried out with scientific rigor [23]. Traits of interest to the farmers are emphasized, and trials are performed using statistically valid designs and research methods. Landraces, conventional varieties, and germplasm from across the region are used in PPB efforts. PPB generates varieties that combine the local adaptation of landraces with improved traits from conventional genotypes such as disease resistance and higher yields. Other traits that factor into selection by farmer-researchers include seed color, appearance and size, pod length, plant architecture, even ripening, early maturity, and various culinary qualities [23]. Zamorano has developed some PPB varieties using landraces as parents in Honduras, Costa Rica, and Nicaragua [31]. Between 1994 and 2015, 24 PPB varieties were developed by Zamorano in collaboration with CIAL groups using participatory research methods, and one of these varieties, ‘PM2-Don Rey’, has been supported for national registration [29]. Adoption of the PPBs among CIAL members is above 60%, and PPBs are gaining wide acceptance among other farmers in communities where participatory research is carried out [29]. Extensive discussion of the development of ‘Macuzalito’, ‘Cedron’, ‘Amilcar’, ‘Esperanceño’, ‘Chepe’, and ‘PM2-Don Rey’ (representing both PPV and PPB methods of variety development) can be found in Humphries et al. [29].

Due to limited production resources and the threat of climate change, farmers in remote hillside communities would benefit from growing high-yielding common bean varieties that are climate resilient and have high nitrogen fixing capacity. To examine Honduran bean germplasm for these traits of interest, we curated a panel of Honduran bean genotypes representative of the traditional landraces and the participatory bred varieties grown by hillside bean farmers, as well as Honduran conventional and North American checks. The current study tests the hypothesis that bean landraces are a good source of germplasm with a high capacity for nitrogen fixation. The objectives of this study were to determine the genotypic and phenotypic diversity of the Honduran panel and to identify germplasm sources for breeding improved varieties suited to hillside production in Honduras.

## 2. Results

### 2.1. Analysis of Genetic Relatedness

**Figure 1:**
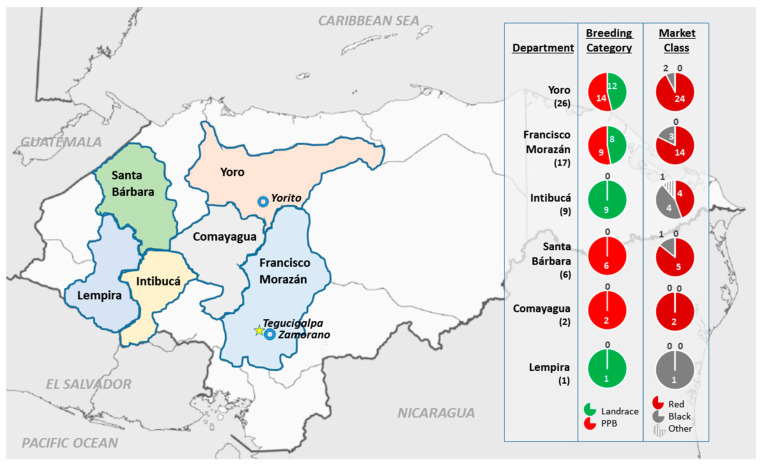
Map of west–central Honduras, outlining the six departments from which landrace and participatory bred (PPB) bean genotypes were sourced for the Honduran Panel. The chart at the right describes the number of landraces and PPB genotypes obtained from each department and the market classes to which those genotypes belong. The location of Yorito where the Honduran field trial was carried out, Zamorano where the *Escuela Agrícola Panamericana* is located, as well as the capital of Honduras, Tegucigalpa, are shown.

Landrace and PPB plant material for the Honduran panel were sourced from six municipalities across west–central Honduras. The majority of genotypes came from Yoro (26) and Francisco Morazán (17), with less than 20 genotypes coming from Intibucá, Santa Bárbara, Comayagua, and Lempira combined (Figure 1). Descriptions of the genotypes can be found in Materials and Methods Section 4.1.

**Figure 2:**
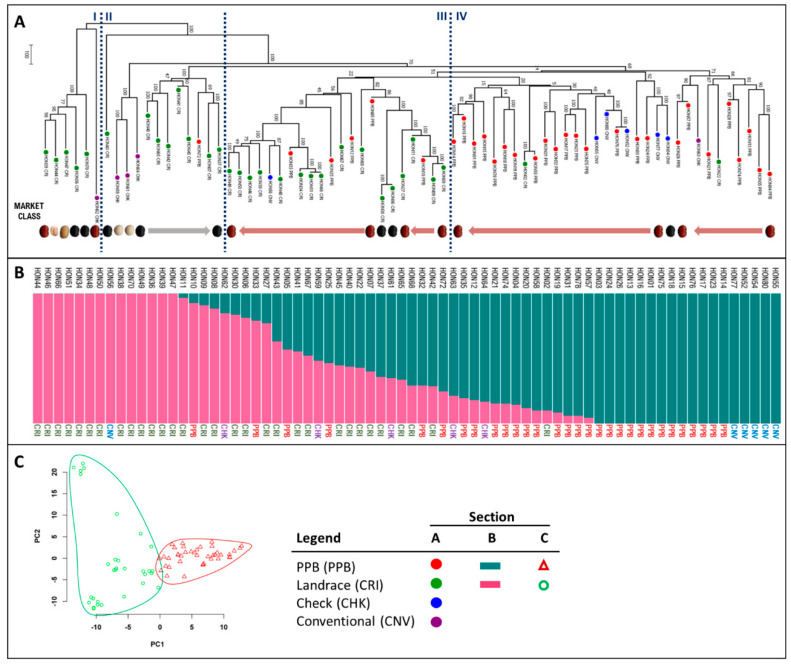
Analysis of genetic structure and relatedness of 72 genotypes of the Honduran panel. (**A**) Dendrogram of evolutionary genetic relatedness. Abbreviations are: participatory bred (PPB), Landrace (CRI), North American check (CHK), and Honduran conventional (CNV) genotypes. Market classes are denoted by representative beans. (**B**) Genetic structure plot using two genetic groupings (ΔK = 2). **C**. Principal component analysis indicating two genetic groupings in the panel. Genotype descriptions are found in Section 4.1. (Note: In section A, grouping names I–IV are assigned to natural subsections of the tree for descriptive purposes and do not correspond to the genetic groups presented in section B.).

The genetic structure of the Honduran panel was explored to determine the evolutionary relatedness of the genotypes in the panel and the genetic composition of the genotypes. It is apparent from the topology of the phylogenetic tree (Figure 2A) that the landrace genotypes (‘CRI’) generally group into clusters of connected branches in the tree structure that are positioned in the left half of the figure, denoted as groupings I, II, and III (Figure 2A). The PPB genotypes (‘PPB’) grouped into separate clusters that are positioned in the right half of the figure, denoted as grouping IV (Figure 2A). Grouping I at the left of the tree, is comprised of the ‘Milpero’ genotypes, two landraces (HON70 and HON43), and Merlot (HON62). The Milpero landraces belong to diverse market classes, including black, small red, white, and carioca, and they included genotypes that did not flower at Elora in 2014. The remaining landrace genotype clusters were generally delineated by market class membership, with black genotypes comprising grouping II (including HON07, HON45, HON41, HON42, HON65, HON40, and HON43) and small red genotypes comprising grouping III (including HON08, HON09, HON11, HON27, HON68, HON67, HON66, HON51, HON34, HON48, HON38, HON46, HON50, and HON49). The landraces ‘Concha Rosada’ (HON02) and ‘Rosado’ (HON22) are displaced and found among the PPB branches of the tree. The North American check genotypes (‘CHK’; including HON64, HON61, and HON59) formed a separate cluster that branched off between the Milpero landraces and the black landraces in grouping II. ‘OAC Rosito’ (HON63), clustered with the Honduran PPBs. All Honduran conventional genotypes (‘CNV’; including black HON54, and small red HON77, HON52, HON80, and HON55) grouped with the PPBs (grouping IV), except ‘Dorado’ (HON56), which is found among the landraces (grouping III). Six PPB genotypes (including HON10, HON05, HON12, HON25, HON33, and HON72) were found within the landrace clusters of the tree (groupings II and III).

The genetic similarity of genotypes in the panel is depicted in a STRUCTURE plot using two subpopulations (*K* = 2) (Figure 2B). Fourteen of the landraces (including all of the Milpero types) belong to one genetic subgroup at the left of the plot and the PPB varieties belong to the other subgroup at the right of the plot, with an intermediary admixed group (Figure 2B). The Honduran conventional genotypes, except ‘Dorado’ (HON56), group with the PPBs. The North American check genotypes are found among the admixed genotypes, along with some PPBs and landraces. The principle component analysis of the panel also indicates the relatedness of the genotypes using two genetic groupings (Figure 2C). PC1 divides the genotypes into PPB (green triangles) and landrace (red circles) categories. Along the PC2 axis, the landraces show wide dispersion, with the Milpero group forming a small cluster near the axis at the top of the plot, and the North American check genotypes are scattered among the landraces. In contrast, PC2 generates a denser cluster of PPB genotypes, and the Honduran Conventional genotypes are at the right edge of the plot.

### 2.2. Nucleotide Diversity and Population Differentiation: Landrace and PPB Categories

**Figure 3:**
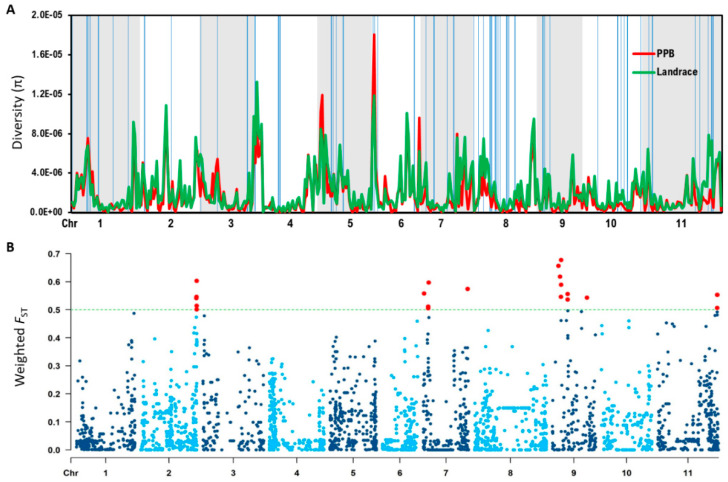
Nucleotide diversity and population differentiation. (**A**) Patterns of nucleotide diversity (π) across the genome between *P. vulgaris* landrace (green) and PPB (red) genotypes. Blue vertical bars show the strongly differentiated (3×) regions. (**B**) Weighted *F*_ST_ plot of genetic variance differentiation among landrace and PPB categories. Significant SNPs are red. Significance threshold *F*_ST_ > 0.5. SNP—single nucleotide polymorphism; Chr—Chromosome.

Nucleotide diversity was measured in the two largest groupings within the Honduran panel, the landraces and the PPBs, to ascertain the genetic diversity of these groups. According to the π statistic, nucleotide diversity for the landrace category overall (π = 3.20 × 10^−4^) was significantly greater (*P* = 0.04, Welch two-sample *t*-test) than that found in the PPB category overall (π = 2.89 × 10^−4^). Additionally, according to the *D* statistic, the overall nucleotide diversity for the landrace category (*D* = 0.669) was significantly greater (*P* = 0.02, Welch two-sample *t*-test) than that found in the PPB category (*D* = 0.476). The positive Tajima’s *D* value indicates that both landraces and PPBs are under balancing selection and implies that both categories are probably experiencing different selective pressure. Fifty-six subregions (>100 Mbp long) across the genome were identified where landrace π values exceeded PPB π values by more than 3 times (Table 1, Figure 3A). These regions, identified on all 11 chromosomes, may contain loci related to traits favored by selection associated with formal plant breeding (Figure 3A).

Calculation of population genetic differentiation (*F*_ST_) between landrace and PPB beans enabled identification of loci under selection between landrace and PPB genotypes. Twenty-six single nucleotide polymorphisms (SNPs) with significant weighted *F*_ST_ values (>0.5) were found on Pv02, Pv07, Pv09, and Pv11 (Table 2, Figure 3B). These SNPs do not fall within the regions of high nucleotide diversity identified in the π comparison above.

### 2.3. Identification of Candidate Genes

Two approaches were used to identify candidate genes associated with regions of significantly high (3×) nucleotide diversity (π) in landraces and high population differentiation (*F*_ST_) between landraces and PPBs, including: exploring the recent bean literature for reported quantitative trait loci (QTL) and searching the bean genome using JBrowse.

QTL associated with various traits have been reported in the literature, including those related to agronomic traits [5,32] and nitrogen fixation [15,33,34,35,36]. Our literature search revealed 10 QTL that fall within regions of significantly high landrace π values, 8 of which are associated with agronomic traits and 2 of which are associated with SNF-related traits. In a GWAS study of agronomic traits in the Middle American Diversity panel (MDP), Wilker et al. (unpublished) found QTL for days to flowering on Pv01 (23.2 Mbp), Pv02 (48.6 Mbp), and Pv06 (13.9 Mbp); QTL for days to maturity on Pv07 (35.6 Mbp) and Pv11 (40.3 Mbp); and QTL for hundred seed weight on Pv01 (23.2 Mbp), Pv05 (32.5 Mbp), and Pv11 (53.5 Mbp). Various candidate genes were found underlying these agronomic QTL and more detailed information is available in Table S1. In a separate GWAS study of agronomic traits in the MDP, Moghaddam et al. [32] found a QTL on Pv01 (42.9 Mbp) associated with growth habit, which contained an RNA polymerase-associated protein RTF1 homolog (Phvul.001G167200). For SNF-related traits, reported QTL that fall within regions of high landrace π values are associated with seed %N content and plant biomass. In a GWAS study of SNF related traits in the MDP, Wilker et al. (unpublished) found a QTL associated with seed %N content at 22.8 Mbp on Pv02. The QTL contains seven putative candidate genes, including a Ras homologous (Rho)/Rho of plants (Rop) family GTPase (Phvul.002G106600). These genes play a role in the symbiotic interaction between the host plant and rhizobia [37]. Two separate studies investigating SNF and related traits in the Andean and the Middle American gene pools identified a QTL associated with shoot biomass at 45.1 Mbp on Pv11 [33,35]. Shoot biomass supports root symbiosis through carbohydrates generated through photosynthesis as well as serving as a sink for N generated through SNF, which is a source of N ultimately stored in the seed [35]. Beyond searching the recent literature for QTL associated with SNF and agronomic traits, we also examined the study by Schmutz et al. [5] which identified >1800 domestication candidate genes in the Middle American gene pool. Over 140 of the domestication genes identified by Schmutz et al. [5] fall within regions of high nucleotide diversity discovered in our study (see Table S1). Two of these genes have a role in symbiosis: Phvul.008G217100 is a short open reading frame (sORF) small protein of the glycerin rich protein family and is expressed during nodule ontology [38]; and Phvul.010G102300 belongs to the plant nuclear factor Y (NF Y) gene family, whose members are involved in nodule ontology, epidermal infection, and rhizobia discrimination [39].

The bean genome was explored using JBrowse in 100 kb segments centered on SNPs with significant genetic differentiation (*F*_ST_) to identify putative candidate genes. All genes found within these regions are listed in Table S2. A diverse range of gene types and functions were seen, including plant defense and stress response genes, enzymes, and transcription factors. The PubMed Central database of NCBI (https://www.ncbi.nlm.nih.gov/pmc/) was used to search for published research on putative candidate genes, and some of those findings are discussed here. The region flanking the significant *F*_ST_ SNP on Pv02 (48.9 Mb) contains two leucine rich repeat disease resistance proteins, Phvul.002G323708 and Phvul.002G323712. This region was identified by Oladzad et al. [40] as a major QTL peak in their GWAS study of *Rhizoctonia solani* resistance in Andean beans. A second region on Pv02 (49.0 Mb) contains a disease resistance gene and one associated with nodulation. Tock et al. [41] found that the pentatricopeptide repeat superfamily protein (Phvul.002G326200) at 49.0 Mb was associated with halo blight damage, while Nova-Franco et al. [42] found that a 1-aminocyclopropane-1-carboxylate oxidase gene (Phvul.002G326600) in this region was associated with nodule senescence. A third region on Pv02 (49.2 Mb) contains a protein kinase superfamily protein (Phvul.002G328300) that Zuiderveen et al. [43] found to be significantly associated with Anthracnose resistance in a GWAS of Andean beans. The region centered at 38.9 Mb on Pv07 contains a protein kinase superfamily protein (Phvul.007G268200), which was downregulated in a slow darkening pinto bean study [44]. On Pv09, the region located at 7.8 Mb contains a GATA transcription factor (Phvul.009G035400), which belongs to a family of transcription factors that have been studied in soybean under nitrogen stress and may play a role in nitrogen metabolism [45]. In the region centered on 13.5 Mb on Pv09, a cytokinin oxidase/dehydrogenase 1 gene (Phvul.009G081800) is located that was found to be upregulated in bean root cortical cells inoculated with arbuscular mycorrhizal fungi under drought stress, compared to noninoculated roots [46].

### 2.4. Diversity for Symbiotic Nitrogen Fixation

The influences of genotype, environment, and the genotype by environment interaction were significant for the combined locations ANOVA for %Ndfa (Table S3); therefore, each environment was analyzed separately for this trait. At each location, significant differences were found between genotypes for %Ndfa (Tables S4–S6). At Elora 2014 (*N* = 49), the average nitrogen fixation capacity was 49.3% and ranged from 21.0% to 64.4%, a difference of 43.4% between the least and most effective genotypes. At Elora 2015 (*N* = 62), the average nitrogen fixation capacity was higher at 55.8%, yet the range for this trait was narrower with a low of 40.5% and a high of 67.3%, a difference of 26.8% between the least and most effective genotypes. At Yorito (*N* = 53), the average nitrogen fixation capacity was 49.0% with a range of 14.0% to 66.4%, a difference of 52.4% between the least and most effective genotypes, which was the greatest range in performance of all locations.

Further, in a separate ANOVA for each location, the genotypes were divided into breeding history categories and their means were compared. In these analyses, significant differences were found among breeding categories at two of the three trial locations. At Elora 2014, the landrace genotypes (*N* = 20, *M* = 52.5 %Ndfa) performed better than all other breeding categories, although the difference between categories was not significant (Table 3). It is evident from the Elora 2014 %Ndfa histogram (Figure S1A) that many landrace genotypes had above average nitrogen fixation performances. At Elora 2015, the Honduran conventional genotypes (*N* = 7, *M* = 59.0 %Ndfa) and the landraces (*N* = 26, *M* = 58.3 %Ndfa) exhibited the best nitrogen fixing capacities, but they were not significantly different from each other (Table 3). The average nitrogen fixing capacities of the North American check genotypes (*N* = 5, *M* = 50.0 %Ndfa) and the PPBs (*N* = 24, *M* = 53.2 %Ndfa) were similar at Elora 2015 and significantly lower than the values for the Honduran conventional and landrace genotypes (Table 4). Of particular note, Merlot (HON62) fixed the most N at Yorito (66.4%), almost 6% more than the next best genotype. This genotype, bred for Northern US production, also performed well at Elora in 2014 (64.3 %Ndfa), but had the worst performance among conventional genotypes at Elora 2015 (43.5 %Ndfa). Merlot has very dark green leaves, an indicator of plant N status, and consistently had high leaf chlorophyll content when measured with the SPAD meter in the Elora trials. (SPAD was not measured at Yorito.) As with the Elora 2014 trial, many landrace genotypes had above average nitrogen fixation performances at Elora 2015 (Figure S1B). At Yorito, the landrace genotypes (*N* = 22, *M* = 46.4 %Ndfa) showed significantly higher nitrogen fixing capacities than the PPBs (*N* = 22, *M* = 40.1 %Ndfa), whereas, the check and Honduran conventional genotypes had intermediary SNF means and did not have significantly different nitrogen fixing performance values when compared to each other nor the other breeding categories (Table 5). As we found at the other trial locations, the landraces showed above average nitrogen fixing performance at Yorito (Figure S1C).

The top five landraces with the highest SNF performance at Yorito were Vaina Rosada (60.6 %Ndfa; HON34), Cincuenteño (59.5 %Ndfa; HON48), Negro Cuarenteño (57.0 %Ndfa; HON42), Olanchano Negro (56.4 %Ndfa; HON65), and Paraísito (53.6 %Ndfa; HON49). These landraces represent both small red and black market classes and are from three different departments (Yoro, Francisco Morazán, and Intibucá). Vaina Rosada, Cincuenteño, and Paraísito are already used in participatory breeding efforts between Zamorano and FIPAH, and a number of the resulting PPB varieties were included in our panel (including HON05, HON23, HON25, HON26, HON28, HON31, HON32, and HON33). The PPB progeny of these landraces ranged in SNF capacity from 26.6 to 53.3 %N at Yorito, which is noteworthy considering SNF was not a breeding objective. Amilcar (53.3 %Ndfa; HON05) is among the top five SNF performing PPB varieties at Yorito, which also included Conan 33 (55.7 %Ndfa; HON24), Campechano (54.5 %Ndfa; HON57), San Jose (51.1 %Ndfa; HON35), and Arbolito Negro (50.8 %Ndfa; HON72). Both small red and black beans are represented in this list, and they come from three departments (Yoro, Francisco Morazán, and Santa Barbara). 

Leaf chlorophyll content was measured at Elora in 2014 and 2015, and these values were analyzed in separate ANOVAs because of the repeated-measure nature of trait data collection. The combined ANOVA indicated significant differences for the fixed effects of genotype, environment, and the genotype by environment interaction at both the early vegetative and reproductive stages (Table S7). When this trait was analyzed by location, significant differences were found between genotypes at both locations and for both growth stages (Table S7).

### 2.5. Diversity for Agronomic Traits

A series of agronomic traits were measured for this study, including carbon discrimination (Δ^13^C) as an indicator of water use efficiency, plant growth stages (days to flowering and maturity), yield (kg ha^−1^), and hundred seed weight. Significant differences were found for the fixed effects of genotype, environment, and the genotype by environment interaction for the agronomic traits carbon discrimination (Δ^13^C), days to flowering (DTF) and days to maturity (DTM), yield (kg ha^−1^), and hundred seed weight (HSW) in a combined ANOVA (Table S3). These traits were therefore analyzed further within locations.

Significant differences were found between genotypes at all locations (Tables S4–S6) for carbon discrimination (in Δ^13^C units) calculated according to the method of Farquhar et al. [47] from seed carbon discrimination (δ^13^C) values obtained from isotope analysis of seed samples. At Elora 2014 (*N* = 48), Δ^13^C values ranged from 18.4‰ to 21.4‰ (Figure S2A), and at Elora 2015 (*N* = 62), the range was similar, from 17.4‰ to 21.1‰ (Figure S2B). At Yorito (*N* = 53), the Δ^13^C values were lower, ranging from 16.4‰ to 20.0‰ (Figure S2C). When genotypes were divided into breeding categories and compared, significant differences were only found at Yorito (Table 5), where the average PPB Δ^13^C value (*N* = 22, *M* = 17.7‰) was significantly lower than the average landrace Δ^13^C value (*N* = 22, *M* = 18.2‰) (Table 5).

Significant differences were found between genotypes in DTF measured at Elora in 2014 and 2015 (Tables S4–S6). At Elora 2014 (*N* = 58), the average was 50 DTF with a range of 42–62 DTF (Figure S3A). Some Honduran genotypes (including HON9, HON13, HON18, HON28, HON32, HON33, HON36, HON39, HON44, and HON47) did not flower at that first trial location and were replaced by different genotypes at the subsequent locations. At Elora 2015 (*N* = 57), the average was 50 DTF with a range of 42–55 DTF (Figure S3B). When genotypes were divided into breeding categories and compared, significant differences were found at Elora 2014 only (Table 2). Overall, the landrace genotypes flowered the earliest (*N* = 22, *M* = 48 days) and they were significantly earlier than the PPB genotypes (*N* = 26, *M* = 52 days) (Table 2).

Significant differences were found among genotypes for DTM measured at Elora in 2014 and 2015 (Tables S4–S6). At Elora 2014 (*N* = 35), the average was 112 DTM with a range of 97–120 DTM (Figure S4A). At Elora 2015 (*N* = 56), the average was 111 DTM with a range of 94–115 DTM (Figure S4B). Significant differences in DTM were found only at Elora 2015 (Table 3), when genotypes were grouped by breeding history and contrasted. At Elora 2015, landraces (*N* = 23, *M* = 109 days) matured significantly earlier than PPBs (*N* = 22, *M* = 112 days).

Significant differences were found among the yields of genotypes in the Elora trials only (Tables S4 and S5). At Elora 2014 (*N* = 35), the average yield was 828.4 kg ha^−1^ with a range from 325.2–1124.2 kg ha^−1^ (Figure S5A). At Elora 2015 (*N* = 62), the average yield was 1558.6 kg ha^−1^ with a range from 600.5–2263.4 kg ha^−1^ (Figure S5B). At Yorito (*N* = 58), the average yield was 791.1 kg ha^−1^ with a range from 299–1471 kg ha^−1^ (Figure S5C). Significant differences were found only in the Elora trials among breeding categories (Table 2 and Table 3). At Elora 2014, the North American check (*N* = 5, *M* = 933.7 kg ha^−1^) and the Honduran landrace (*N* = 14, *M* = 912.8 kg ha^−1^) genotypes yielded significantly more than the other categories (Table 2), and at Elora 2015, the PPBs (*N* = 23, *M* = 1686.2 kg ha^−1^) yielded significantly more than the landraces (*N* = 27, *M* = 1396.5 kg ha^−1^; Table 3). At Yorito, the Honduran conventional genotypes (*N* = 6, *M* = 956.5 kg ha^−1^) returned the highest yields, followed by the PPBs (*N* = 24, *M* = 823.2 kg ha^−1^), while the landraces (*N* = 24, *M* = 745.2 kg ha^−1^) and the North American checks (*N* = 4, *M* = 669.9 kg ha^−1^) had lower yields (Table 4).

Significant differences were found among hundred seed weight (HSW) calculated for samples from the Elora trials (Tables S4 and S5). At Elora in 2014 (*N* = 49), the average HSW was 20.2 g with a range from 13.3–29.4 g. At Elora in 2015 (*N* = 62), the average HSW was 21.4 g with a range from 14.5–31.9 g. No significant differences were found among genotypes grouped by breeding history (data not shown).

Plant height was measured only at the Yorito location (*N* = 60), and significant differences were found between genotypes for this trait (Table S6). The average height was 35.3 cm with a range of 4 cm to 47 cm. Significant differences were not found when breeding history categories were contrasted (data not shown).

### 2.6. Trait Correlation and Genotype by Trait Biplot Analyses

**Figure 4:**
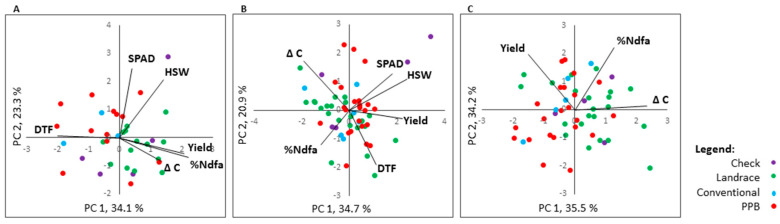
Biplot analysis of traits for genotypes of the Honduran panel in three location years. (**A**) Elora 2014; (**B**) Elora 2015; and (**C**) Yorito 2014–15. DTF, days to flowering; Yield, yield (kg ha^−1^); HSW, 100 seed weight (g); Δ C, carbon discrimination; %Ndfa, percent nitrogen derived from the atmosphere; and SPAD, leaf chlorophyll content at 100% flowering.

Pearson correlation analyses were performed on LSmeans for each trial environment to determine trait interactions (Table S8). In addition, trait correlations and genotype performance were visualized using genotype × trait biplots for each location (Figure 4). In the biplots, positive correlations between traits are evidenced by vectors forming acute angles, for example between SPAD and HSW at Elora in 2014 and 2015 (Figure 4B), whereas negative correlations between traits are evidenced by obtuse angles formed between vectors, such as that formed by DTF and yield at Elora in 2014 (Figure 4A). A right-angle formed between trait vectors indicates a weak or lack of association between those traits. The results of our correlation and biplot analyses support each other.

#### 2.6.1. %Ndfa

At Elora in 2014, %Ndfa was negatively correlated with DTF (*r* = −0.31) but was positively correlated with Δ^13^C (*r* = 0.45) and with yield (*r* = 0.38) (Table S8). In the Elora 2014 biplot (Figure 4A), the landrace genotypes are clustered towards the yield and the %Ndfa vectors. The Honduran conventional genotypes are more closely associated with the DTF vector, as are the majority of the Honduran conventional genotypes (Figure 4A).

At Elora in 2015, %Ndfa was not significantly associated with any other trait (Table S8). The biplot analysis showed that DTF and Δ^13^C are not associated with %Ndfa, and leaf chlorophyll content at flowering (SPAD) and HSW have a negative relationship with %Ndfa (Figure 4B). As in the Elora 2014 biplot, the landrace genotypes cluster towards the %Ndfa vector (Figure 4B).

At Yorito, %Ndfa was not significantly correlated with other traits (Table S8). The biplot analysis for Yorito shows landrace genotypes cluster more towards the %Ndfa and Δ^13^C vectors, whereas the PPB genotypes cluster away from the %Ndfa vector and are more closely associated with the yield vector (Figure 4C).

#### 2.6.2. Agronomic Traits

Leaf chlorophyll content at the early reproductive stage (SPAD) was positively correlated with hundred seed weight (HSW) at both Elora 2014 (*r* = 0.36) and Elora 2015 (*r* = 0.44) (Table S8). DTF was negatively associated with yield at Elora 2014 (*r* = −0.48), but no association was found between these traits at Elora 2015. DTF was negatively associated with Δ^13^C at Elora 2015 (*r* = −0.37), but no association was found between these traits at Elora 2014 (Table S8). Yield was positively associated with HSW (*r* = 0.49) and negatively associated with Δ^13^C (*r* = −0.33) at Elora 2015; however, these associations were not repeated at the other trial locations (Table S8).

#### 2.6.3. High-Yielding and High-Fixing Genotypes

**Figure 5:**
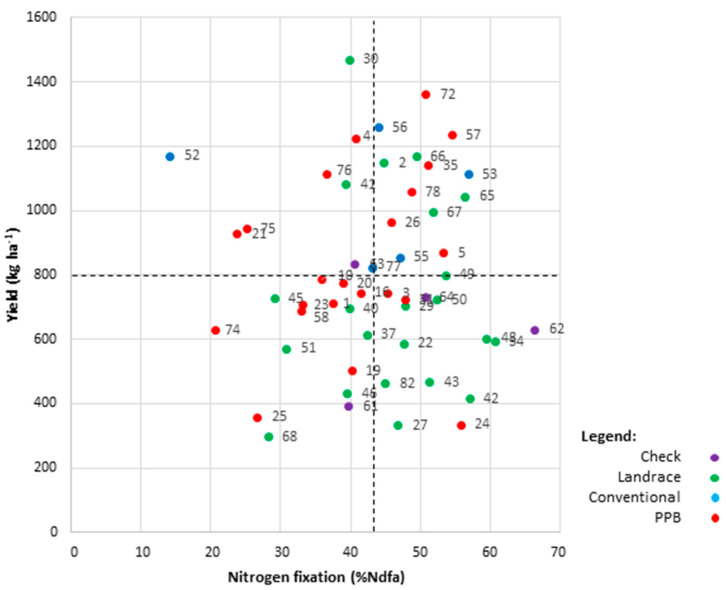
Genotype performance for nitrogen fixation (x axis) and yield (y axis) at Yorito. For this trial, average yield was 799 kg ha^−1^ and average nitrogen fixation was 43.3 %Ndfa, and these values are shown on the plot in dashed lines, dividing the plot into quadrants. Genotypes in the upper right quadrant are higher yielding and higher nitrogen-fixing.

The aim of any breeding program is to generate high-yielding genotypes, and in this study an additional goal was to identify genotypes that were also high-N fixing. It is particularly useful to examine genotype performance at Yorito, where growing conditions are representative of the marginal production regions in Honduras. At Yorito, 14 genotypes had above-average yields coupled with above-average SNF performance (Figure 5). These included four Honduran conventional varieties (HON56, HON53, HON55, and HON77), four landraces (HON2, HON49, HON65, and HON66) and six PPB varieties (HON5, HON26, HON35, HON57, HON72, and HON78). Of the six PPB varieties, five were developed through participatory varietal selection, and one was developed through participatory plant breeding. These genotypes are dispersed throughout the phylogenetic tree (Figure 2A), suggesting a lack of close genetic relatedness; however, three of the high-yielding high-fixing PPB varieties share common genotypes in their pedigrees: HON5 and HON26 have a common landrace parent, Cincuenteño (HON48); HON26 shares a conventional parent, Tio Canela 75 (HON55), with HON78; and, HON5 has Amadeus 77 as a parent, which is a daughter of Tio Canela 75. There was no apparent relationship between release date and higher yields, nor was there a temporal trend for SNF performance.

## 3. Discussion

### 3.1. Genotype Origins and Pedigree Explain Honduran Panel Structure

The patterns observed in the phylogenetic tree and STRUCTURE diagrams derived from the SNP compositions of the lines in the Honduran panel largely agree with what is known about their geographic origins and their pedigrees, but there are also a few exceptions. The two large groupings in the dendrogram based on SNP similarities (groups I–III and IV) show a clear separation (with some exceptions) between landraces (CRI) and material that has been conventionally bred or is the product of participatory plant breeding (PPB). Among the landraces, the small red beans that came from Yoro, Francisco Morazán, and Intibucá, were randomly interspersed throughout group III of the phylogenetic tree with no particular pattern, based on genotype origin. In contrast, clustering of genotypes by region of origin is found among the black bean landraces (groups I and II). Group I consists of the black bean landraces, which came from Intibucá, Yoro, Francisco Morazán, and Lempira, and contains the Milpero landraces (HON36, HON47, HON44, and HON39) and ‘Negro Opalaca’ (HON70), which are all from Intibucá, and ‘Negro’ (HON43), which is found alone on the next branch is from the neighboring department, Lempira [‘Merlot’ (HON62) is also found in this region of the tree and is discussed below]. These landraces are the most distantly related genotypes with respect to the rest of the panel. The Milpero landraces were daylight sensitive when grown at Elora in 2014, and their photoperiod sensitivity was likely inherited from a common ancestor. All photoperiod sensitive varieties in the panel may carry the dominant *ppd* gene responsible for control of this trait [48]. The remaining black landraces from Yoro, Francisco Morazán, and Intibucá are found without any particular pattern throughout the next branch of the tree, and two are found among the small red landrace branches (HON06 and HON30). The STRUCTURE analysis shows that most of the black genotypes are admixed, suggesting a closer genetic relationship to the conventional and PPB germplasm. No small red landraces were found within the black landrace branches; however, two small red landraces, ‘Concha Rosada’ (HON02) and ‘Rosado’ (HON22), are displaced and found in group IV among the PPBs. The STRUCTURE analysis shows that Concha Rosada contains only ~10% genetic material from the landrace subpopulation, and Rosado is almost equally admixed between the landrace and PPB subpopulations. In the case of Concha Rosada, this may be explained by the fact that this landrace is widely grown and has been used as a parent in participatory breeding efforts and thus shares ancestry with many PPB varieties. Alternatively, Concha Rosada may not be a traditional landrace but instead a creolized variety derived from a formal-sector variety introduced to the Yorito region in the early 1980s [23]. The reason that Rosado is found among the PPB varieties in the tree is less apparent. Rosado has not been used as a parent for any of the PPB varieties in the panel according to the pedigree information available. Rosado recently arrived in the Yorito region, and a survey of bean farmers revealed that its origin is unknown [29]. According to M. Gomez (FIPAH), the initial population of Rosado showed phenotypic heterogeneity, and some selection has been made to create a uniform line for PPB breeding in collaboration with Zamorano. At Elora 2014, Rosado had uneven maturity, which may indicate that the seed planted that year, and the seed grown for DNA extraction, was not a fixed homogeneous line, and this heterogeneity may have resulted in misplacement of this genotype in the phylogenetic tree.

The organization of the PPB branches of the phylogenetic tree can be explained in part by common ancestry. The Honduran conventional varieties, ‘Tio Canela 75’ (HON55) and ‘Amadeus-77’ (HON52), and the landraces, ‘Estica’ (HON11) and ‘Vaina Rosada’ (HON34), have been used frequently in generating the PPB genotypes in this panel. For example, five of the seven genotypes in the left-most PPB branch (HON19, HON18, HON15, HON16, and HON14) are derived from crosses with Tio Canela 75 or Estica, or both. The STRUCTURE plot indicates that these genotypes contain a greater proportion of conventional/PPB than landrace genetic material, and therefore, they are found within the PPB branches of the tree. However, six PPB varieties (HON10, HON05, HON12, HON25, HON33, and HON72) are found among the landrace branches of the tree, which indicates that they share greater genetic similarity to their landrace parent than the other genotypes in their pedigrees. For example, FPV 921-65 (HON33) has the landrace Vaina Rosada (HON34) and Amadeus 77 in its pedigree. FPV 921-65 is found in the same branch as Vaina Rosada among the landraces in the tree, and the STRUCTURE analysis shows that FPV 921-65 has more similarity to the landrace genetic subgroup. Marcelino (HON10) and Amilcar (HON05) also contain >50% landrace genetic material (Figure 2B) and are found among the landrace branches. Fourteen PPB genotypes have between 5% and 50% admixture with the landrace subgroup. Alleles favoring agronomic characteristics for local adaptation and culinary traits contributed by landrace parents were likely prioritized under selection among PPB progeny from crosses between landrace and conventional genotypes, resulting in PPB varieties consisting of a large proportion of landrace genetic material.

Common parentage may explain the clustering of the five Honduran conventional genotypes (Amadeus-77, CENTA San Andrés, Tio Canela 75, Aifi Wuriti, and Carrizalito) in the panel. CENTA San Andrés, Amadeus-77, and Carrizalito have common ancestry. Tio Canela 75 is a parent of breeding line EAP 9510-77, which was released in the early 2000s in countries across Central America, including in El Salvador, as CENTA San Andrés, and in Honduras, as Amadeus-77. Tio Canela 75 is also a parent of EAP 9510-1, a sister line of EAP 9510-77, which was released in Honduras as Carrizalito in 2003 and as Telire in Costa Rica in 2004 [49]. Accordingly, these genotypes are closely associated in the phylogenetic tree, although some genetic differentiation has occurred between CENTA San Andres and Amadeus-77. The line ‘MD 30-75’ (released as Tio Canela 75) was used as a parent in generating four of the five conventional genotypes’ mentioned above. This is likely a result of the effort to introduce BGMV resistance into Central American germplasm, as MD 30-75 is a highly resistant line, which carries the *bgm-1* resistance gene [50]. DEORHO (HON53) was not genotyped in our study; however, it also has MD 30-75 in its pedigree, and it is reasonable to suppose that it would also appear in this region of the dendrogram. Aifi Wuriti was the only black conventional genotype included in the panel. Interestingly, it is most closely related to three small red genotypes (Carrizalito, Conan 33, and Cedrón) rather than the small black genotypes, although Aifi Wuriti does not appear in the pedigrees of any of the small black PPB genotypes in the panel. The final Honduran conventional genotype, ‘Dorado’, is unusual because it is found among the landrace genotypes in the phylogenetic tree and the STRUCTURE analysis. This may be explained by the lack of a common genotype in its pedigree compared to the other conventional genotypes.

The placement of the North American check varieties (except OAC Rosito) as a separate group within the dendrogram is consistent with the unique alleles that they would be expected to have relative to the genotypes in this panel. The location of the check varieties in the landrace portion of the tree (groups I and II) may reflect the genetic diversity and wide-ranging geographic origins of the germplasm used in the University of Guelph/AAFC and Michigan State University/USDA-ARS bean breeding programs, which are aimed at introducing resistance to abiotic and biotic stresses and improving various agronomic traits. The locations of the North American checks interspersed throughout the admixed portion of the STRUCTURE plot also indicate that genetic diversity has been retained in these modern North American genotypes. OAC Rosito is a special case, since it was derived from El Salvadoran germplasm, and it is found among the PPBs in the phylogenetic analysis where it is most closely related to two PPB genotypes, Campechano (HON57) and Quebradeño (HON26). According to the STRUCTURE analysis, OAC Rosito is more genetically similar to the Honduran Conventional/PPB sub-group than the landraces. This suggests either that El Salvadoran landraces are not similar to Honduran landraces, or more likely that the landrace population from which OAC Rosito was developed was actually a creolized conventional variety. Germplasm provided by Zamorano has been used in El Salvadoran variety development since the early 2000s [26], and this could explain the genetic similarity of OAC Rosito to the PPB varieties in our panel, which were developed in collaboration with Zamorano.

### 3.2. Optimizing Use of Genetic Diversity of Honduran Landraces and PPB Varieties

The larger nucleotide diversity among the landraces (π = 3.20 × 10^−4^) than observed in the PPBs (π = 2.89 × 10^−4^) in the current study is consistent with general observations that landraces are more diverse than materials that are products of selection [51,52,53,54]. However, other studies that compared diversity in wild to domesticated bean accessions found wider diversification between those groups than we found between landraces and PPBs in our panel. Nanni et al. [55] reported that within the Mesoamerican gene pool, nucleotide diversity was 3.2 times higher among wild genotypes (π = 17.34 × 10^−3^) than domesticated genotypes (π = 5.43 × 10^−3^). The difference between landraces and PPBs in the current study was only 1.1 times, probably because of the small population size and because these genotype groups do not represent extremes of the diversity continuum that was sampled in the previous study. Both landraces and PPBs are selections from wild accessions, and the PPBs have probably not been as strongly selected as conventionally derived varieties.

The high level of diversity in Honduran landraces suggests they could be a source of novel alleles that could be used in breeding to improve various traits. Landraces are adapted to the environmental conditions of the locations where they were maintained, in some cases, over thousands of years. Landraces that were grown in fluctuating environments and in low-input agricultural systems may be enriched for rare alleles enabling phenotypic plasticity and inherent responsiveness to diverse abiotic and biotic stresses [56]. Landraces, in regions where they are still grown, have often been pushed to marginal production environments where their performance often exceeds that of modern cultivars [29,57,58].

Dry bean landrace germplasm across Mesoamerica is genetically diverse [59]. Soil conditions across this region are poor, and the terrain ranges from low to high altitude with steep slopes, leading to certain trait adaptations in the landraces. For example, ‘Common Red Mexican’, a red-seeded landrace from Mexico, has been found to be drought resistant [60], while ‘Puebla 152’, a black-seeded landrace also from Mexico, has superior SNF capacity [61]. Originating in the Andean region, G19833, a ‘Chaucha Chuga’ landrace from Perú, has tolerance to high levels of soil aluminum and low levels of phosphorus [56] and resistance to a number of bean diseases [62,63]. Our survey of the literature found genes in high landrace π regions associated with abiotic stress tolerance, phosphorus use efficiency, and nitrogen fixation (see Table S1). Conservation of landraces and mobilization of the unique genetic diversity they contain through plant breeding can help address the future need for higher yielding and climate resilient varieties.

### 3.3. Regions of High Genetic Differentiation Indicate Regions Impacted by Selection

Genetic divergence between the PPB and landrace subpopulations in the panel is indicated by regions of high genetic differentiation (*F*_ST_), and these genomic regions may contain loci that have been subjected to selection pressure. We identified several regions on chromosomes Pv02, Pv07, Pv09, and Pv11 where *F*_ST_ values exceeded 0.5. Similarly, in a study of genetic diversity of Italian bean landraces, Lioi et al. [64] reported that genomic regions related to domestication were concentrated on Pv02, Pv07, and Pv09 for Mesoamerican types. For comparisons between wild and domesticated bean landraces, Papa et al. [65] also reported significantly larger levels of *F*_ST_ differentiation around genomic regions associated with domestication. While the genetic distance between the Honduran landraces and PPB genotypes included in our study is not likely as wide as that between the wild and landrace genotypes investigated by Papa et al. [65], similar trends towards genetic differentiation between landrace and PPB genotypes developed with modern breeding objectives and germplasm could be expected.

In particular, the genomic regions with large *F*_ST_ differences may be associated with traits that were a focus of selection in PPB breeding. However, an extensive search of the recent bean literature did not reveal any known QTLs associated with agronomic, SNF, or WUE traits that are located within the regions of large *F*_ST_ differentiation identified in this study. This may be because this is the first genomic survey study of Honduran material, and the distinguishing traits between landraces and PPB materials are specific to materials from that region or expressed in that location. In particular, because we are comparing two domesticated groups of genotypes, namely farmer traditional landraces and PPB varieties, the genes underlying the regions of large differentiation found in our study could be those responsible for local adaptation, culinary qualities, and favorable plant traits, rather than traits associated with domestication [5]. Additionally, the conventional germplasm used to generate PPB genotypes, either through crosses with landraces or through varietal selection, is largely limited to material in the Zamorano breeding program, which may have a specific genetic architecture.

In spite of the lack of previous QTL evidence for selection for domestication in the high *F_ST_* regions, several genes in those regions that have been studied for various reasons may be associated with domestication. For example, disease resistance genes, such as those found on Pv02 for Rhizoctonia resistance (Phvul.002G323708, Phvul.002G323712; [40]), Halo blight resistance (Phvul002G326200; [41]), and Anthracnose resistance (Phvul.002G328300; [43]), and on Pv08 for Anthracnose resistance (Phvul.008G019600; [66]), and for the bean-rust interaction (Phvul.008G270500; [67]), have been associated with domestication in several crops [68,69]. Genes controlling agronomic traits have been identified in domestication studies in other crops [70,71,72]. Additionally, genes that affect survival in diverse growing conditions may have also been favored over the course of domestication. Two such genes are located in a region of high genetic diversity on Pv08; the ethylene-responsive transcription factor (Phvul.008G019600; [73]), an ortholog of an Arabidopsis gene known to be involved in flooding tolerance [74], and the transcription factor IIIA (Phvul.008G270400), which is upregulated in phosphorus-restricted conditions [75]. Soltani et al. [73] suggest that further studies are needed to understand the process of local adaptation and allelic selection using bean landraces and wild populations. Insight to develop climate-resilient crops can be drawn from the study of crop adaptation under natural selection and domestication [76,77].

### 3.4. Landraces are Superior Nitrogen Fixers

Although some genotype- and environment-influenced variability was seen in our study, our examination of symbiotic nitrogen fixation in the Honduran panel revealed a wide range of capacity for this trait. The superiority of the landraces for SNF capacity at all trial locations may be the consequence of the continual selections of these materials under conditions of low soil fertility endemic to Central America. Even today, these materials continue to be grown by small scale farmers who do not have access to fertilizer inputs. Under these conditions, bean genotypes which have developed efficient associations with nitrogen-fixing bacteria would have a larger source of nitrogen for metabolic processes and better phenotypic fitness compared to poor nitrogen-fixing genotypes. Strong nitrogen-fixers would have a competitive advantage in the low input environments and would likely be preferentially selected over time. There may be parallels between the selection pressures during landrace evolution and the selection of heirloom bean varieties, which have also been shown to have strong SNF capacity [18].

There are few studies that have investigated SNF capacity of bean landrace genotypes. Heilig et al. [33] used ‘Puebla 152’, a black-seeded Mexican landrace known for its nitrogen fixing capacity [78], in a cross with conventional genotype ‘Zorro’ to create a RIL population to study SNF. The authors found that Puebla 152 fixed between 13.0 to 45.5% of the nitrogen in samples (seed + biomass), which was slightly more than Zorro, which fixed between 5.4 to 44.4% [33]. Many landraces in our study fixed more N than Puebla 152, indicating that Honduran landraces may be a useful source of SNF capacity. The SNF performance of Zorro ranged from 47.7 to 53.0%Ndfa in our study, a mid-range performance among our check genotypes, and overall better than its performance in Heilig’s study [33].

The SNF performance of the progeny of the cross between the conventional genotype Zorro and the landrace Puebla 152 may be predictive of the performance of PPB varieties that are crosses between Honduran conventional varieties and landraces. The SNF performance of Honduran PPB varieties ranged between 20.5 to 55.7%Ndfa at Yorito. Although the focus of the participatory breeding program between FIPAH and Zamorano has been to generate higher-yielding genotypes, rather than on improving SNF performance, the SNF capacity seen for the PPB varieties falls within the upper range of that found for the RILs in Heilig’s study [33].

For the Honduran panel, the insights gleaned from Yorito are of particular interest because this location has growing conditions representative of small-scale growers across the region, and as much as possible, local growing practices were employed in the trial. At Yorito, there was a range in SNF performance in the landraces, and overall genotypes belonging to this breeding history group performed better than the others studied. In addition, PPB genotypes derived from crosses with the best SNF-performing landraces had strong SNF capacity. Zamorano used the methods of participatory varietal selection to develop these PPB varieties with CIALs, enabling local growers to evaluate genotype performance on their farms. For example, Amilcar and San Jose were tested by various CIALs through the regional adaptation nursery (VIDAC, Vivero de Adaptación Centroamericano) in the mid-2000s. Amilcar has the high-SNF performing landrace Cincuenteño in its pedigree. Generally, native Rhizobia inhabit tropical soils and farmers do not use Rhizobia inoculants, although Zamorano disseminates SNF knowledge through the CIALs, including effective Rhizobia inoculants and protocols for use. In addition, the breeding program has the capacity to test SNF performance in ‘*bancales*’ where soil N levels are low and SNF-related traits, such as nodulation, can be observed. PPB breeding for enhanced SNF capacity could be expanded if grower demand and the threat of climate change and resulting raising input costs warrant it.

In addition, the range in SNF performance among the conventionally bred North American checks and Honduran conventional varieties was wide. The superior SNF capacity Merlot exhibited at Yorito would suggest it has value as a breeding parent for this trait in Honduras; however, it has a larger seed size and a dark red seed coat; traits that are less preferred by Honduran consumers and could be challenging to select against in a breeding program. Of the Honduran conventional genotypes, DEORHO (HON53) fixed the highest amount of N (56.8%), but it performed poorly in Elora fixing 34.4% (2014) and 52.6% (2015) of its N. It is not found in the pedigrees of any PPB genotype included in our panel, but DEORHO has been a popular variety in commercial growing regions of the country. It has disease resistance, high yield, and the preferred light red seed coat color, making it a good candidate for future PPB breeding efforts.

### 3.5. PPB Genotypes Have Superior Water Use Efficiency Values

Plants that have higher water use efficiency (WUE) are more drought tolerant, and WUE can be estimated using carbon differentiation (Δ^13^C) values measured from plant biomass. During photosynthesis, plants discriminate against the incorporation of the heavy C isotope (^13^C), depleting ^13^C in plant biomass and driving lower Δ^13^C values [79]. Plants with comparatively low biomass Δ^13^C values can be considered more drought tolerant. WUE has been studied in beans, including landraces. A study by Munoz-Perea et al. [80] of the WUE of 16 dry bean genotypes in drought-stressed and nonstressed environments found that the two landraces included differed in their responses to drought stress, but Common Red Mexican was among the best performers under drought stress conditions. In contrast, in our study, the significantly lower Δ^13^C values measured in Yorito for the PPB genotypes than the landraces indicates that the PPB varieties in our panel may be more resilient to drought conditions than the landraces.

The drought resistant characteristics of the PPBs were likely contributed by the conventional parents. For example, PM2-Don Rey (HON23) was the most WUE PPB genotype at Yorito, with a Δ^13^C value of 16.36‰. PM2-Don Rey was developed through PPB methods from a cross between the landrace, Paraísito, and the Honduran conventional variety, Carrizalito. It was released as a drought-resistant variety in 2016 [81], and it is the first variety from the EAP-Zamorano-CIAL PPB collaborations to be released at the national level. A second PPB genotype, Marcelino (HON10), was developed through participatory varietal selection, and it had similar WUE (16.43‰) to PM2-Don Rey. The PPBs FPY-724-43 (HON16; 17.03‰), Cedrón (HON03; 17.41‰), and Amilcar (HON05; 17.42‰) have the next best WUEs.

Three landraces had WUE values below 17.5‰, including Concha Rosada (HON02; 16.85‰), Chapin Rojo (HON27; 16.86‰), and Chirineño (HON67; 17.30‰). Concha Rosada is of particular note because it is favored by poor farmers for its early maturity, which allows it to escape drought conditions late in the growing season [29,30]. Our study indicates that Concha Rosada not only has drought resistance through ‘drought escape’ but also has WUE characteristics that enable it to survive drought.

Among the conventionally bred genotypes, Carrizalito (HON77) and OAC Rosito (HON63) were the most water use efficient with low Δ^13^C values of 17.47‰ and 17.54‰ at Yorito. Carrizalito is a commercial Honduran variety, and it was used as a parent contributing disease resistance, agronomic, and likely WUE traits, to PM2-Don Rey. Among the check genotypes, OAC Rosito, recently developed at the University of Guelph from an El Salvadoran plant introduction, had the best WUE performance. This characteristic is likely retained from its domestication in Central America, and this enabled it to outperform the other check genotypes that have been developed for production in the Great Lakes region of North America.

In the coming decades, the effects of climate change are predicted to bring drier conditions to Honduras, and drought-resistant crops will help protect yields through periods of minimal rainfall. It has been proposed that WUE can be improved through selection and breeding. In alfalfa, evaluating genotypes for Δ^13^C and selecting for lower Δ^13^C values has been used to improve WUE in this important forage species [79]. The current results indicate that there is variation among the Honduran PPB and conventional bean germplasm in WUE traits, and selecting for lower Δ^13^C values could be applied to beans in Honduras to generate improved varieties that are more resilient to drought conditions.

### 3.6. Conventional Genotypes Have Superior Yields

Releasing varieties with higher yields is the objective of modern breeding programs, and our trial results suggest that improvements impacting yield have been made along the breeding history continuum from landraces to PPBs to conventional varieties. Considering the Honduran germplasm, the landraces were the lowest-yielding group at Yorito, followed by PPBs and the conventional varieties. This result is counter to the findings of early experiments performed by CIALs, where landraces out-yielded conventional materials [23]. However, our trial was conducted at the FIPAH office in Yorito, where soil fertility is less restrictive and the plot is flat, whereas the early CIAL trials were conducted in farmers’ fields, which have low-fertility soils and sloped land; conditions for which conventional materials were not developed. The superior performance of the conventional materials in our trial at Yorito is consistent with the aim of modern breeding practices in generating higher yielding varieties. The North American checks were the highest yielding at Elora 2014, as they were bred for performance in this region, whereas the Honduran conventional and PPBs performed poorly at Elora 2014. The landraces also performed well at Elora 2014, and this may be attributed to phenotypic plasticity resulting from retention of useful nucleotide diversity enabling them to perform well in a new environment.

### 3.7. Utility of Panel Genotypes for Breeding

The different breeding and/or selection histories for the materials contributing to the phenotypic diversity present in the Honduran panel may provide opportunities for improving different traits in beans in the same way that a number of studies with different crops have found unique benefits from the use of landraces. In wheat, for example, cultivation of landraces in low-input systems has led to the conservation of traits that increase the duration of photosynthesis, which can lead to an increase in grain yield [82]. In a study comparing barley landrace and modern cultivar performances under stress conditions, the landraces were higher yielding and were less likely to fail outright [58]. The advantage of using landraces as parents in breeding programs has also been explored. In a study examining barley yields under drought conditions, progeny from crosses using landrace genotypes were found to be higher yielding than progeny from crosses without landraces in their pedigrees [56]. The authors concluded that breeding crops for vulnerable environments could be enhanced by identifying landrace alleles associated with yield performance and abiotic stress adaptation and employing these in breeding programs [56].

In the current study, landraces, which had superior symbiotic nitrogen fixation characteristics could be excellent sources of novel alleles for this trait. Similarly, PPB materials, which had superior WUE, and cultivated varieties, which had superior yields within their target environments, might be exploited, respectively, for these purposes. In general, all the germplasm types that were tested represent useful resources for breeding for important traits in the face of climate change and increasing production costs/demands.

The diversity for SNF capacity inherent in Honduran bean landraces, and their unique adaptation to the microclimates where they are grown, leads us to conclude that the inclusion of landrace germplasm in breeding for enhanced SNF would produce high fixing genotypes with growth and culinary characteristics already accepted by small-scale bean growers.

## 4. Materials and Methods

### 4.1. Plant Material

The Honduran Panel was assembled in 2014 at the University of Guelph in collaboration with agronomists at FIPAH. The initial panel contained 27 landraces, 26 PPB varieties, and 5 Honduran conventional checks provided by FIPAH, as well as 6 North American checks sourced from the University of Guelph bean breeding program.

The landraces consisted of traditional inbred varieties unimproved by modern plant breeding, which are grown by subsistence farmers in hillside communities. The PPB varieties were generated either through participatory varietal selection (PVS) or participatory plant breeding (PPB) through a collaboration between the bean breeding program at Zamorano and CIALs associated with FIPAH. The landraces and PPB varieties were sourced by M. Gomez (FIPAH) from six departments in west–central Honduras. The landraces were from Yoro, Intibucá, Francisco Morazán, and Lempira, and the PPB varieties were from Yoro, Francisco Morazán, Santa Bárbara, and Comayagua (Figure 1). Seed was either collected directly from farmers in their communities or sourced from central seed banks maintained by FIPAH and PRR. The five Honduran conventional checks were developed for production in lower to mid-altitude, hillside and valley commercial-production regions of the country. The six North American varieties consisted of Merlot and OAC Rosito as small red market class checks, Zorro as a black market class check, and three navy beans: OAC Mist, a high-nitrogen-fixing genotype, R99, a nonfixing mutant, and its parent line OAC Rico. All genotypes in the panel belong to race Mesoamerica [83].

In the first trial location (Elora 2014), 10 Honduran genotypes were found to be daylight sensitive and were not grown at the subsequent locations. Additionally, seed of 16 genotypes that exhibited uneven maturity in Elora 2014 were sent to Puerto Rico for seed increase over the winter of 2015. For the second trial location (Yorito, 2014–2015), 12 new genotypes (6 landraces, 5 improved, and 1 Honduran conventional check) were added to the panel. For the third trial location (Elora 2015), seed harvested from Elora 2014, from the Puerto Rican seed increase, and from Yorito were used, as available. An additional Honduran conventional variety was grown that year to fill in the experimental design. Overall, a total of 77 genotypes were tested in the Honduran panel, 50 genotypes of which were grown at all 3 locations. A summary of the genotypes included in the Honduran Panel, including trial year, market class, seed source, and pedigree information is provided in Table 6, Table 7 and Table 8 according to breeding history.

### 4.2. Field Experimental Design and Maintenance

Field trials were carried out at the University of Guelph Elora research station (ERS) in summer 2014 and 2015 and at Yorito, Honduras in the *Postrera* season (planted December, 2014).

#### 4.2.1. Elora

The Elora fields were selected based on low soil nitrogen levels as measured by preplant soil tests and by site crop rotation histories that indicated that no dry bean crops had been produced in those fields for the previous decade at a minimum. In 2014, nitrates (NO_3_^−^) were low at 7.1 ppm, and ammonium (NH^+^_4_) levels were 3.2 ppm. In 2015, nitrates (NO_3_^−^) were very low at 4.8 ppm, and ammonium (NH^+^_4_) levels were 4.5 ppm.

A square lattice design (8 × 8) with two replications was used for each trial. At the ERS in 2014, 135 seeds of each genotype were grown in 4-row plots (150 cm × 190 cm, 37.5 cm between rows) with approximately 6 cm between plants. At the ERS in 2015, 60 seeds of each genotype were grown in 4-row plots (150 cm × 90 cm, 37.5 cm between rows) with approximately 5 cm between plants within rows.

Clean seed of each genotype was coated with commercially available Nodulator (Becker-Underwood) *Rhizobium leguminosarum* bv *phaseoli* peat-based inoculant prior to planting. The day before planting, inoculant powder (1/4 teaspoon, approximately 0.4 g in 2014; 1/8 teaspoon, approximately 0.2 g in 2015) was added to each seed envelope and the contents were shaken to coat the seeds. Inoculated seed was stored at the ERS at 4 °C until planting to maintain inoculant viability. The entire contents of each envelope (coated seed + loose inoculant powder) was planted. Successful inoculation was confirmed each year by observing pink (active) nodules on a few plants chosen at random throughout the trial at flowering time.

At the ERS, plots were maintained with standard practices throughout the growing season, except no-nitrogen fertilizer was used. Preplant fertilizer (0-20-20) at a rate of 200 kg ha^−1^ was applied approximately one week prior to planting. Preplant herbicide was applied to control broadleaf weeds, and pesticides were applied as needed throughout the growing season at standard rates to control leafhoppers, anthracnose, and root rot (see details in Wilker et al. [18]). Plots were manually weeded once before canopy closure each year.

At Elora 2014, the harvest was staggered according to maturity. The plots were pulled by hand at maturity and threshed at the side of the field using a Wintersteiger plot combine (Wintersteiger AG, Upper Austria, Austria) with a Classic Seed-Gauge weighing system by Harvest-Master (Juniper Systems Inc., Logan, UT, USA), and plot seed weight and moisture content were recorded. Plots that did not reach reproductive/physiological maturity were not harvested. In 2015, plot harvest took place after all plots reached maturity with an SPC20 Almaco plot combine (ALMACO, Nevada, IO, USA), which automatically recorded moisture and weight (kg ha^−1^) for each plot at 13% moisture.

#### 4.2.2. Yorito

The Yorito trial site was chosen based on field uniformity, access to irrigation, and proximity to the FIPAH regional office. Soil NO_3_^−^ levels were 18 ppm (“moderate” to “high”) at Yorito, and the field had been used for bean and maize production in previous seasons. The FIPAH agronomist, M. Gomez, indicated that the trial site conditions were representative of the bean production areas around Yorito.

Seed of each North American check variety were provided by the University of Guelph, and seed of all Honduran bean genotypes in the trial were sourced in Honduras. A square lattice design (8 × 8) with two replications was used for the trial. At Yorito, 100 seeds of each genotype were grown in 2-row plots (100 cm × 500 cm, 50 cm between rows) with approximately 30 cm between plants and 3 seeds sown per hole, as per the traditional planting system.

Inoculant for the trial, a mixture of *Rhizobium etli* (CIAT 632) and *R. tropici* (CIAT 899) strains, was provided by J.C. Rosas (EAP-Zamorano) and was applied according to a protocol provided by *PIF* at a rate of 500 g ha^−1^ [93]. Briefly, the peat-based inoculant powder was applied to slightly moistened seed to ensure it adhered well to the seeds, and once sown, the seeds were covered with soil to protect the inoculant from the sun. CIAT 899 has high symbiotic stability and efficient N-fixation characteristics [15].

Plots were maintained with standard practices throughout the growing season. A preplant fertilizer of 12-24-12 NPK was used at a rate of 64.81 kg ha^−1^ as a formulation without N was not available in Honduras. Carbendazim (DEROSAL, Bayer) at a rate of 400 mL ha^−1^ was used to protect the trial from angular leaf spot (*Pseudoscercospora griseola*), common bacterial blight (*Xanthomonas axonopodis* pv. *phaseoli*), and rust (*Uromyces appendiculatus*).

At Yorito, plots were harvested by hand over a number of days as each plot matured. Yield (kg ha^−1^) was measured at harvest, the two plots of each genotype were bulked, and a subsample of seed was sent to Guelph for further analyses.

### 4.3. Phenotyping

#### 4.3.1. Elora

As plots initiated the reproductive phase, DTF was recorded as the date when 50% of the plants in a plot had one flower open. Relative leaf chlorophyll content was measured twice during the growing season (when the mean number of plots had reached (1) the second trifoliate stage and (2) at 100% flowering) using an SPAD 502 Plus Chlorophyll Meter (Konica-Minolta). The meter was calibrated according to manufacturers’ instructions each time the unit was powered-on (https://www.specmeters.com/assets/1/22/2900P_SPAD_502.pdf). The middle leaflet in the top-most, fully expanded trifoliate leaf was used for the measurements, and three plants were sampled at random per plot.

As plots reached physiological maturity, DTM was recorded as the date when plots were ready to harvest. Three plants were randomly sampled from mature plots, placed in large paper bags, and dried in a repurposed tobacco kiln (De Cloet Bulk Curing Systems, model TPG-360, Tillsonburg, ON, Canada) at 33 °C at the ERS for 24–48 h. Roots were cut from each plant and the above-ground biomass was weighed. Plants were then threshed using an indoor belt thresher (Agriculex SPT-1A, Guelph, ON, Canada), their seed collected, weighed, and counted. Hundred seed weights (HSW) were calculated.

#### 4.3.2. Yorito

As plots initiated the reproductive phase, DTF was recorded as the date when 50% of the plants in a plot had one flower open. Disease ratings and agronomic scores were collected throughout the growing season; however, statistical analyses revealed no significant differences between genotypes, and these traits are not further reported here. DTM was recorded as the date when 90% of the pods in a plot had changed color.

### 4.4. Isotope Analysis

Seed from each plot was processed and analyzed as detailed in Wilker et al. [18]. Briefly, seed was finely ground, precisely measured, and the isotope analyses were carried out using mass spectrometry at the Agriculture and Agri-food Canada (AAFC) gas chromatography mass spectrometry facility in Lethbridge, Alberta. Samples were analyzed for δ^15^N (‰) and δ^13^C (‰).

To calculate the percent nitrogen derived from the atmosphere (%Ndfa), the natural abundance method was used on seed samples in this study [94]. Seed N represents the total N accumulated by a plant over the course of the growing season, and seed N values are representative of whole-plant N values [21]. Additionally, processing seed samples is more efficient than shoot tissue.

The natural abundance method uses the following equation,

$$\%Ndfa=\frac{\delta^{15}N_{reference\;plant}-\delta^{15}N_{fixing\;plant}}{\delta^{15}N_{reference\;plant}-B}$$

where *δ*^15^*N_ref. plant_* is the rate of *δ*^15^*N* in the reference genotype (R99), *δ*^15^*N_fixing plant_* is the *δ*^15^*N* of the N-fixing bean genotype, and B is the average *δ*^15^*N* of beans grown in an environment where its entire N source is from fixation [95]. The B-value was obtained for this experiment as described by Farid (2015) [36]. Briefly, *δ*^15^*N* was measured and averaged for 20 bean genotypes from both the Andean and Middle American gene pools, which were grown in a growth room in N-free media. Normalized *δ*^15^*N* values were used for all genotypes, and an average of *δ*^15^*N* values for R99 were used in %Ndfa calculations.

To estimate water use efficiency, δ^13^C values obtained from GCMS seed analysis were used following the methods proposed by Farquhar et al. [47]. Because the current WUE discussion utilizes Δ^13^C values, the raw δ^13^C values were converted to Δ^13^C using the following equation:

$$\Delta C=\frac{\delta^{13}C_{air}-\delta^{13}C_{plant}}{1+\delta^{13}C_{plant}}$$

where *δ*^13^*C_air_* is the current free atmospheric level of approximately −8‰ and *δ*^13^*C_plant_* is calculated per plant seed sample using appropriate C isotope standards. For example, a plant with a δ^13^C value of −28.2‰ yields Δ^13^C = (−0.008 + 0.0282)/(1 − 0.0282) = 0.0202/0.9718 = 20.7‰.

### 4.5. Genotyping

To enable discovery of the genetic structure of the HON panel, 73 genotypes (see Table 1) were genotyped for single nucleotide polymorphisms (SNPs). DNA was extracted following the same methods outlined in Wilker et al. [18]. Briefly, plants were grown in a controlled environment (16 h photoperiod, 22 °C) at the University of Guelph, and young-leaf tissue samples were harvested, freeze dried, and the DNA extracted according to manufacturer’s instructions using the NucleoSpin Plant II kit (Macherey-Nagel, Dueren, Germany). DNA of adequate quality was sent to the Genome Quebec Innovation Centre (McGill University, Montreal) for SNP genotyping using the Illumina Infinium iSelect Custom Genotyping BeadChip (BARCBEAN6K_3) containing 5398 SNPs [96]. TASSEL was used to filter the SNP data (MAF > 0.01) to a set representing 72 individuals and containing 4314 polymorphic SNPs [97]. Missing data comprised less than 3% of the data, and these were subsequently imputed using Beagle v4.1 [98] as described by Torkamaneh and Belzile [99].

### 4.6. Population Structure

The population structure of the Honduran panel was determined using a number of methods, as follows. First, the population structure was estimated using variational Bayesian inference implemented in fastSTRUCTURE [100]. Five runs were performed for each number of populations (K) set from 1 to 10 using the 4.3K genome-wide SNPs identified in this study. A ChooseK analysis was conducted to determine the number of subpopulations that maximize the marginal likelihood. Then, a principal component analysis (PCA) was conducted in TASSEL and plotted using PCAshiny in R [101]. Finally, the evolutionary relationships among the genotypes of the panel were inferred using the Neighbor-Joining method with the genome-wide SNP data [102]. The taxa were clustered together using the bootstrap test (1000 replicates) [103]. The tree was drawn to scale, with branch lengths (next to the branches) in the same units as those of the evolutionary distances used to infer the phylogenetic tree. The evolutionary distances were computed using the Maximum Composite Likelihood method and the units correspond to the number of base substitutions per site. Evolutionary analyses were conducted in MEGA7 [104,105].

### 4.7. Genetic Diversity

The levels of genetic diversity in the landrace and PPB breeding history categories of the HON panel were assessed using the 4.3K SNP dataset and VCFtools [106]. The π statistic provides an indication of polymorphism within a population as measured by nucleotide diversity, and Tajima’s D (*D*) provides an indication of selection pressure [107,108]. Both π and *D* were measured in sliding windows of 1 Mb across the genome using the—window-pi and—TajimaD options in VCFtools [106], which resulted in an average of 6 SNPs per window. The pairwise π and *D* values were also calculated among different subpopulations. Genome-wide averages of π and *D* for each breeding history category were generated by taking the average across all windowed calculations. Landrace and PPB π values were compared across the genome, and regions where landrace π exceeded PPB π by more than 3 times were considered highly differentiated, and the regions that were at least 25,000 bp in length were considered significant. To investigate the level of differentiation between the landrace and PPB genotypes, the *F*_ST_ statistic was computed. *F*_ST_ was calculated using the—weir-fst-pop option in VCFtools in sliding windows of 100 bp across the genome [106]. Weighted *F*_ST_ values range from 0 with no genetic differentiation to 1 where fixation of alleles has occurred. *F*_ST_ values exceeding 0.5 were considered significant in our analysis.

### 4.8. Candidate Gene Investigation

A literature search was carried out to identify previously reported QTL and genes that colocalize with regions where π values were highly differentiated between the Landrace and PPB categories. JBrowse (https://legumeinfo.org/genomes/jbrowse/) was used to explore the bean genome around SNPs with significant weighted *F*_ST_ values in order to identify candidate genes. A 100 kb region centered on each significant marker was searched.

### 4.9. Statistical Analysis

Analysis of variance (ANOVA) tests were performed on the phenotypic data collected from each environment and all environments combined, using the GLIMMIX procedure in SAS (version 9.4, SAS Institute, Cary, NC, USA, 2012). In the combined analysis, genotype, environment, and the genotype-by-environment interaction were considered fixed effects, while all other effects and their interactions were considered random. In the separate environment analyses, genotypes were considered fixed effects, while all other effects and the interaction effects were considered random. The Shapiro–Wilk test was performed on the residuals in the UNIVARIATE procedure to test their normality [109]. Random and independent distributions of the residuals were visually examined by plotting the studentized residuals against the predicted values. Data that generated outlier residuals were removed from the data set. Further, single degree of freedom contrasts were conducted in GLIMMIX between breeding history categories—landraces, PPBs, and conventional and check genotypes—contrasting each category to each of the others. Repeated measures of leaf chlorophyll content (SPAD) were taken, and separate ANOVA tests were used to compare SPAD values at each time point in a combined analysis and by environment. The least squared means (LSmeans) for each trait were computed using the LSMEANS statement in the GLIMMIX procedure for each genotype.

Using the LSmeans calculated above, the pair-wise Pearson’s coefficients of correlation were computed for all traits in the CORR procedure in SAS. The PRINCOMP and PRINQUAL procedures were used in SAS to generate the principal component (PC) values, to estimate the proportion of variance accounted for by each PC, and to plot PC1 against PC2 to generate genotype x trait (GT) biplots to determine genotype and trait interactions in each environment [110].

## 5. Conclusions

The aim of our study was to evaluate a large set of Honduran landraces and varieties generated through participatory plant breeding, as well as check conventional genotypes, to ascertain their value in future breeding efforts. We used simple genomics and phenotyping to characterize the panel. Our genetic analyses found that the panel is divided into predominantly-landrace and predominantly-PPB groupings, with Honduran conventional genotypes sharing most similarity to the PPBs. Breeding history and pedigrees account for this division. The genetic diversity analysis revealed that landraces have retained a higher level of nucleotide diversity than PPB genotypes, which we attribute to selection pressure imposed by breeding for different production environments/objectives, and the use of a small number of conventional/elite parents in breeding efforts. The nucleotide diversity inherent in landraces can be used to increase the frequency of rare alleles in breeding programs. Beyond genetic characterization, it is important to classify germplasm for trait phenotypic diversity, which could be employed in breeding, because landraces that have evolved in adverse environments contribute adaptive traits to variety development.

Two traits that contribute to climate resiliency, nitrogen fixation capacity, and water use efficiency were evaluated in our study. Genotypes with good nitrogen fixation capacity are an asset for remote hillside growers who have limited funds and limited opportunity to purchase inputs because of poor market access. Landraces were shown to have superior SNF capacity and are already favored by hillside producers. Genotypes with enhanced water use efficiency will also be an asset to hillside growers in a future with drier and more changeable growing conditions. The PPB and conventional varieties in our study show promising characteristics for drought resilience. Further evaluation of PPB varieties under drought conditions is warranted.

Honduran bean production will continue to be carried out predominantly by small-scale hillside producers. The widely grown farmer landraces are locally adapted and accepted by consumers, and future breeding efforts should deliver varieties that maintain the inherent SNF capacity of landraces, while enhancing drought resilience and producing high yields. PPB methods employed in the breeding efforts between EAP-Zamorano and FIPAH- and PRR-supported CIALs have succeeded in generating promising PPB varieties. One variety that combines these characteristics is Amilcar (HON05). It is a small red PPB variety selected among germplasm provided by Zamorano for improvement by a CIAL in Yoro. Ultimately, Amilcar has been widely accepted because of its commercial value, culinary qualities, and disease resistance, but its climate resiliency traits and yield potential are of critical importance. Landrace characterization, conservation, and employment in breeding programs will bring continued benefits.

## Figures and Tables

**Table 1:** Regions of the *P. vulgaris* (2.0) genome where high nucleotide diversity (π) was discovered in landrace genotypes compared to PPB genotypes. A literature search was performed to identify candidate genes within these regions. See Table S1 for candidate gene annotation. (Chr—Chromosome).

	Region of High Diversity		π Value		Candidate Genes
Chr	Start (Mbp)	End (Mbp)	Landrace	PPB	Number
**1**	23	24	5.50 × 10^−7^	1.31 × 10^−7^	2
**1**	40	41	7.71 × 10^−7^	1.83 × 10^−7^	4
**1**	41	42	1.24 × 10^−6^	2.49 × 10^−7^	4
**1**	42	43	1.22 × 10^−6^	3.98 × 10^−7^	1
**1**	47	48	8.60 × 10^−7^	9.66 × 10^−8^	3
**2**	4	5	1.95 × 10^−6^	6.51 × 10^−7^	0
**2**	22	23	1.71 × 10^−6^	1.64 × 10^−7^	8
**2**	32	33	5.53 × 10^−7^	1.22 × 10^−7^	5
**2**	48	49	5.92 × 10^−6^	1.62 × 10^−6^	18
**3**	34	35	6.73 × 10^−7^	1.97 × 10^−7^	8
**3**	48	49	1.17 × 10^−6^	1.89 × 10^−7^	8
**4**	12	13	1.53 × 10^−6^	2.62 × 10^−7^	0
**4**	17	18	4.05 × 10^−7^	6.55 × 10^−8^	0
**4**	38	39	4.74 × 10^−7^	9.66 × 10^−8^	6
**4**	40	41	4.32 × 10^−7^	1.27 × 10^−7^	0
**5**	24	25	3.55 × 10^−7^	6.55 × 10^−8^	1
**5**	25	26	2.10 × 10^−6^	1.92 × 10^−7^	0
**5**	27	28	7.46 × 10^−7^	1.31 × 10^−7^	1
**5**	32	33	1.16 × 10^−6^	3.28 × 10^−7^	11
**6**	9	10	1.21 × 10^−6^	6.55 × 10^−8^	7
**6**	13	14	1.02 × 10^−6^	1.31 × 10^−7^	14
**6**	15	16	4.82 × 10^−7^	1.55 × 10^−7^	6
**7**	8	9	4.32 × 10^−7^	9.66 × 10^−8^	1
**7**	20	21	5.27 × 10^−7^	6.55 × 10^−8^	1
**7**	26	27	3.98 × 10^−6^	1.01 × 10^−6^	0
**7**	35	36	3.36 × 10^−6^	7.38 × 10^−7^	9
**7**	39	40	2.43 × 10^−6^	3.32 × 10^−7^	8
**8**	11	12	5.44 × 10^−6^	1.74 × 10^−6^	1
**8**	15	16	1.63 × 10^−6^	5.32 × 10^−7^	2
**8**	18	19	1.30 × 10^−6^	3.98 × 10^−7^	1
**8**	23	24	5.27 × 10^−7^	6.55 × 10^−8^	0
**8**	29	30	1.35 × 10^−6^	3.32 × 10^−7^	0
**8**	38	39	3.29 × 10^−7^	3.33 × 10^−8^	0
**8**	41	42	7.62 × 10^−7^	2.26 × 10^−7^	2
**8**	44	45	1.35 × 10^−6^	1.62 × 10^−7^	0
**8**	45	46	9.15 × 10^−7^	2.90 × 10^−7^	0
**8**	51	52	2.86 × 10^−6^	6.33 × 10^−7^	6
**8**	52	53	1.21 × 10^−6^	9.66 × 10^−8^	12
**8**	57	58	3.45 × 10^−7^	6.55 × 10^−8^	0
**9**	16	17	7.81 × 10^−7^	1.60 × 10^−7^	0
**9**	18	19	9.04 × 10^−7^	3.33 × 10^−8^	3
**9**	21	22	1.94 × 10^−6^	4.99 × 10^−7^	0
**10**	17	18	2.97 × 10^−7^	9.32 × 10^−8^	0
**10**	33	34	2.26 × 10^−6^	7.41 × 10^−7^	0
**10**	35	36	9.78 × 10^−7^	2.62 × 10^−7^	2
**10**	37	38	1.07 × 10^−6^	1.94 × 10^−7^	7
**10**	39	40	3.00 × 10^−6^	7.34 × 10^−7^	1
**11**	2	3	8.74 × 10^−7^	1.93 × 10^−7^	3
**11**	7	8	8.00 × 10^−7^	1.55 × 10^−7^	23
**11**	10	11	1.37 × 10^−6^	3.01 × 10^−7^	7
**11**	37	38	2.64 × 10^−6^	7.84 × 10^−7^	1
**11**	40	41	9.07 × 10^−7^	3.01 × 10^−7^	3
**11**	45	46	7.85 × 10^−6^	9.80 × 10^−7^	9
**11**	47	48	7.31 × 10^−6^	1.53 × 10^−6^	0
**11**	48	49	1.52 × 10^−6^	4.99 × 10^−7^	0
**11**	53	54	6.90 × 10^−7^	2.28 × 10^−7^	11

**Table 2:** Regions of the *P. vulgaris* (2.0) genome where SNPs with significantly high weighted *F*_ST_ values (>0.5) were found. JBrowse was used to search for candidate genes within 100 Kb of significant SNPs. Candidate gene descriptions are listed in Table S2. (https://legumeinfo.org/genomes/jbrowse/). (Chr—chromosome).

Chr	SNP Position (Mbp)	*F*_ST_ Value	Number of Candidate Genes
2	48.9	0.541	8
	49.1	0.546	11
	49.1	0.603	13
	49.2	0.514	10
	49.2	0.501	
7	0.6	0.558	15
	0.7	0.558	
	4.2	0.505	13
	4.2	0.511	
	4.7	0.597	7
	38.9	0.574	10
9	5.5	0.656	8
	6.9	0.618	3
	7.7	0.546	5
	7.8	0.677	9
	7.9	0.589	
	13.5	0.536	11
	13.6	0.536	
	13.7	0.556	11
	30.6	0.543	6
11	52.4	0.553	10
	52.4	0.506	
	52.4	0.553	
	52.4	0.553	
	52.5	0.553	
	52.5	0.553	

**Table 3:** F-test of fixed effect breeding category in the GLIMMIX analysis, and the breeding category LSmeans comparisons of genotypes in the HON panel grown at Elora, 2014.

	N Derived from the Atmosphere (%)		Carbon Discrimination (Δ) (‰)		Flowering (Days)		Maturity (Days)		Yield (kg ha^−1^)	
	*F*	*Pr > F*	*F*	*Pr > F*	*F*	*Pr > F*	*F*	*Pr > F*	*F*	*Pr > F*
**Breeding category**	1.86	0.1441	1.51	0.2195	6.13	0.0007	3.06	0.0368	7.15	0.0004
	LSmean *	***SE***	LSmean *	***SE***	LSmean *	***SE***	LSmean *	***SE***	LSmean *	***SE***
**Check**	51.4 ^a^	0.04	20.2 ^a^	0.23	48.6 ^ab^	1.44	114.9 ^a^	2.0	933.7 ^a^	53.18
**Conventional**	43.3 ^a^	0.04	19.6 ^a^	0.24	51.0 ^ab^	1.44	108.6 ^a^	2.82	700.9 ^ab^	77.10
**Landrace**	52.5 ^a^	0.02	20.1 ^a^	0.14	48.2 ^b^	0.87	109.9 ^a^	1.19	912.8 ^a^	28.39
**PPB**	46.7 ^a^	0.02	19.9 ^a^	0.14	51.6 ^a^	0.83	114.0 ^a^	1.26	721.6 ^b^	30.51

* Means labeled with different letters within trait categories are significantly different according to ANOVA, *p* = 0.05.

**Table 4:** F-test of fixed effect breeding category in the GLIMMIX analysis, and the breeding category LSmeans comparisons of genotypes in the HON panel grown at Elora, 2015.

	N Derived from the Atmosphere (%)		Carbon Discrimination (Δ) (‰)		Flowering (Days)		Maturity (Days)		Yield (kg ha^−1^)	
	*F*	*Pr > F*	*F*	*Pr > F*	*F*	*Pr > F*	*F*	*Pr > F*	*F*	*Pr > F*
**Breeding category**	6.69	0.0004	0.88	0.4535	0.94	0.4225	3.27	0.0251	3.66	0.0148
	LSmean *	***SE***	LSmean *	***SE***	LSmean *	***SE***	LSmean *	***SE***	LSmean *	***SE***
**Check**	50.0 ^a^	0.03	19.8^a^	0.32	50.8 ^a^	0.83	113.1 ^ab^	1.65	1454.7 ^ab^	145.59
**Conventional**	59.0 ^bc^	0.02	19.8^a^	0.29	49.6 ^a^	0.75	109.9 ^ab^	1.42	1613.2 ^ab^	121.38
**Landrace**	58.3 ^b^	0.01	19.5^a^	0.24	49.3 ^a^	0.36	109.0 ^a^	0.63	1396.5 ^a^	58.55
**PPB**	53.2 ^ac^	0.01	19.6^a^	0.24	49.3 ^a^	0.36	112.1 ^b^	0.65	1686.2 ^b^	62.74

* Means labeled with different letters within trait categories are significantly different according to ANOVA, *p* = 0.05.

**Table 5:** F-test of fixed effect breeding category in the GLIMMIX analysis, and the breeding category LSmeans comparisons of genotypes in the HON panel grown at Yorito, 2014–2015.

	N Derived from the Atmosphere (%)		Carbon Discrimination (Δ) (‰)		Days to Flowering (Days)		Yield (kg ha^−1^)	
	*F*	*Pr > F*	*F*	*Pr > F*	*F*	*Pr > F*	*F*	*Pr > F*
**Breeding category**	3.72	0.0143	3.18	0.0280	3.60	0.0163	1.34	0.2647
	LSmean *	***SE***	LSmean *	***SE***	LSmean *	***SE***	LSmean *	***SE***
**Check**	49.5 ^ab^	0.04	18.0 ^ab^	0.25	39.0 ^ab^	1.28	669.9 ^a^	135.22
**Conventional**	40.1 ^ab^	0.03	18.0 ^ab^	0.22	36.8 ^ab^	1.06	956.5 ^a^	108.87
**Landrace**	46.4 ^a^	0.01	18.2 ^a^	0.10	36.2 ^a^	0.53	745.2 ^a^	52.16
**PPB**	40.1 ^b^	0.01	17.7 ^b^	0.10	38.3 ^b^	0.54	823.2 ^a^	52.62

* Means labeled with different letters within trait categories are significantly different according to ANOVA, *p* = 0.05.

**Table 6:** Genotypes of the North American check and Honduran conventional breeding categories that were included in the HON panel tested at three field locations, 2014–15. The trials in which each genotype was included and whether the entry was SNP genotyped are indicated. Breeding category, market class, genealogy, and origin are provided where available.

									Origin	
HON Entry	VARIETY	Elora ‘14	Yorito ‘14-’15	Elora ‘15	SNP Genotyped	Breeding Category	Market Class	Genealogy	Institution or Organization	Notes
**59**	OAC Rico	x		x	x	Check	Navy	(Ex Rico 23/Narda)/Ex Rico 23 *See* [84]	University of Guelph	Resistant to BCMV and Anthracnose. Tolerant to white mold. Unremarkable SNF capacity. In other studies it fixed approximately 53% of N [18].
**60**	R99^1^	x	x	x	x	Check	Navy	*See* [85]	Agriculture Agri-Food Canada (AAFC)	Non-nodulating experimental line. Derived from OAC Rico through ethyl methan sulphonate (EMS) mutagesis [86]. Used in natural abundance method to establish a baseline nitrogen level in seed measured for ^14^N and ^15^N accumulation.
**61**	OAC Mist	x	x	x	x	Check	Navy	*See* [87]	University of Guelph	High yielding, late season. Resistant to BCMV and CBB. Generally high SNF capacity. Farid and Navabi (2015) reported that OAC Mist fixed as much as 78.5% of N [16]. Wilker et al. [18] reported that OAC Mist fixed an average of 55% N.
**62**	Merlot	x	x	x	x	Check	Small red	*See* [88]	United States Department of Agriculture—Agriculture Research Service (USDA-ARS)	Intense red seed color. Larger seed size than Honduran beans (mean HSW 39.2 g). Resistant to rust, BCMV, and BCMNV. Susceptible to anthracnose. Moderate SNF capacity. Wilker et al. (unpublished) found Merlot fixed as much as 64.9 % of its N.
**63**	OAC Rosito	x	x	x	x	Check	Small red	*See* [89]	University of Guelph	Developed from a diverse landrace originating in El Salvador [89]. Dark red seed color. Similar seed size to Honduran beans (mean HSW 21.7 g). Resistant to BCMV. Susceptible to Anthracnose and CBB. The SNF capacity of OAC Rosito has not been examined previous to the current study.
**64**	Zorro	x	x	x	x	Check	Black	*See* [90]	Michigan State University	Resistant to rust and anthracnose and is less affected by white mold. Moderate SNF capacity. Wilker et al. [18] reported that Zorro fixed an average of 59%, and Wilker et al. (unpublished) found that Zorro fixed as much as 46.9% of its N.
**80**	CENTA San Andrés ^3^			x	x	Conventional	Small red	EAP 9510-77, [MD 30-75/DICTA 105]	PIF/Zamorano, UPR, CENTA, El Salvador; 2003	Same breeding line as Amadeus-77 [49]. Resistant to BGYMV and BCMV. Heat tolerant and adapted for production in lower-altitude coastal areas [49].
**52**	Amadeus-77 ^3^	x	x	x	x	Conventional	Small red	EAP 9510-77, [MD 30-75/DICTA 105]	PIF/Zamorano, UPR, DICTA, Honduras; 2003	Same breeding line as CENTA San Andres [49]. Resistant to BGYMV and BCMV. Heat tolerant and performs well in low altitude, coastal areas. Widely adopted across Central America, and in 2010, accounted for around 50% of commercial production in the region [26].
**56**	Dorado ^3^	x	x	x	x	Conventional	Small red	DOR 364, [BAT 1215 x (RAB 166 x DOR 125)]	Profrijol, DICTA, Honduras; 1990	Also known as ‘DOR 364’. Resistant to BGYMV and BCMV [49]. Yields well across environments and has mid-range maturity; however, it has a dark red seed coat [49].
**53**	DEORHO ^2,3^	x	x	x		Conventional	Small red	SRC 2-18-1, [Milenio/MD 30-75]	PIF/Zamorano, UPR, DICTA, Honduras; 2007	Also known as ‘DEHORO’ and ‘INTA Matagalpa’. Resistant to BGYMV and BCMV. Higher yielding and desirable light red seed coat color [91]. Popular with Honduran growers, accounting for 23% of the red bean acreage in 2010 [26]. DEORHO was not grown for DNA extraction and consequently was not included in the genetic analyses carried out for this study.
**55**	Tio Canela 75 ^2,3^	x	x	x	x	Conventional	Small red	MD 30-75, [DOR 483//DOR 391/Pompadour J]	PIF/Zamorano, UPR, Honduras; 1996	Resistant to BGYMV and BCMV [49]. Yields well across environments, has mid-range maturity, and has a shiny red seed [49]. Tio Canela 75 is a parent line of Amadeus-77 and Carrizalito.
**77**	Carrizalito ^3^		x	x	x	Conventional	Small red	EAP 9510-1, [MD 30-75/DICTA 105]	PIF/Zamorano, UPR, DICTA, Honduras; 2003	Resistant to BGYMV and BCMV. Early maturity (68–70 DAP) and upright plant architecture. High yielding variety, adapted to mid-altitude production [91].
**54**	Aifi Wuriti ^2^	x	x	x	x	Conventional	Black	EAP 9712-13, Tio Canela 75/DICTA 105/BG12WB12//Tio Canela 75/DICTA 105/AL12	PIF/Zamorano, UPR, SNS, Haiti; 2008	Also known as ‘Negro Olfirwit’. Resistant to BGYMV, BCMV, is tolerant of low soil fertility, and is early-maturing [92]. Popular in Haiti and the Dominican Republic and was successfully adopted by growers in southeast Guatemala [92].

^1^ R99 was genotyped but not included in the genetic analyses (Figure 2). ^2^ Genotypes exhibiting uneven maturity at Elora 2014 and sent to Puerto Rico for seed increase in winter 2015. ^3^ Varieties developed using conventional breeding methods according to J.C. Rosas, pers. comm.

**Table 7:** Genotypes of the Landrace breeding category that were included in the HON panel tested at three field locations, 2014–15. The trials in which each genotype was included and whether the entry was SNP genotyped are indicated. Breeding category, market class, and origin details are provided where available.

								Origin			
HON Entry	VARIETY	Elora ‘14	Yorito ‘14–’15	Elora ‘15	SNP Genotyped	Breeding Category	Market Class	Institution, Farmer, or Organization	Locality	Municipality	Department
**02**	Concha Rosada ^2^	x	x	x	x	Landrace	Small red	FIPAH	Yorito	Yorito	Yoro
**06**	Negro Pedreño	x	x	x	x	Landrace	Black	Odir Palma	La Esperanza	Yorito	Yoro
**07**	Negro Concha Blanca ^2^	x	x	x	x	Landrace	Black	Odir Palma	La Esperanza	Yorito	Yoro
**08**	Balin Rojo ^2^	x	x	x	x	Landrace	Small red	Edy Hernandez	La Patastera	Yorito	Yoro
**09**	Carmelita ^1^	x			x	Landrace	Small red	Francisco Murillo	La Esperanza	Yorito	Yoro
**11**	Estica	x		x	x	Landrace	Small red	Irene Hernandez	La Esperanza	Yorito	Yoro
**22**	Rosado ^2^	x	x	x	x	Landrace	Small red	Odir Palma	La Esperanza	Yorito	Yoro
**27**	Chapin Rojo	x	x	x	x	Landrace	Small red	Daniel Vargas	El Injerto	Comayagua	Francisco Morazán
**29**	Uva	x	x	x		Landrace	Black	Alonso Gutierrez	San Jose	Vallecillo	Francisco Morazán
**30**	Chapin Negro ^2^	x	x	x	x	Landrace	Black	Ovidio Valeriano	Nocoro	Vallecillo	Francisco Morazán
**34**	Vaina Rosada	x	x	x	x	Landrace	Small red	CIAL San Jose	San Jose	Vallecillo	Francisco Morazán
**36**	Milpero Negro ^1^	x			x	Landrace	Black	Bertilio Antonio Rodriguez	San Pedrito	Opalaca	Intibucá
**37**	Negro Vaina Blanca	x	x	x	x	Landrace	Small red	Carmen Azucenaa Giron	Guayabal	Jesus de Otoro	Intibucá
**38**	Mano de Piedra	x	x	x	x	Landrace	Small red	Maria Laines	Maye	Jesus de Otoro	Intibucá
**39**	Milpero Rojo ^1^	x			x	Landrace	Small red	Bertilio Antonio Rodriguez	San Pedrito	Opalaca	Intibucá
**40**	Vaina Blanca	x	x	x	x	Landrace	Black	Tiburcio Dias	Pueblo Viejo	NA	NA
**41**	Negro Arbolito	x	x	x	x	Landrace	Black	Armando Pineda	Crucita Oriente	Jesus de Otoro	Intibucá
**42**	Negro Cuarenteño	x	x	x	x	Landrace	Black	Maria Laines	Maye	Jesus de Otoro	Intibucá
**43**	Negro ^2^	x	x	x	x	Landrace	Black	Antonio Espinosa	Iguala	Lempira	Lempira
**44**	Milpero Gatiador ^1^	x			x	Landrace	Carioca	Evelino Sanchez	La Vegas	NA	NA
**45**	Ponga la Olla	x	x	x	x	Landrace	Black	Estalin Diaz	Pueblo Viejo	NA	NA
**46**	Madura Parejo	x	x	x	x	Landrace	Small red	Maria Lainez	Maye	Jesus de Otoro	Intibucá
**47**	Milpero Blanco ^1^	x			x	Landrace	White	Maria Juana Gutierrez	Monte Verde	Opalaca	Intibucá
**48**	Cincuenteño	x	x	x	x	Landrace	Small red	FIPAH	Yorito	Yorito	Yoro
**49**	Paraísito	x	x	x	x	Landrace	Small red	FIPAH	Yorito	Yorito	Yoro
**50**	Rojo de Seda	x	x	x	x	Landrace	Small red	FIPAH	Yorito	Yorito	Yoro
**51**	Marciano	x	x	x	x	Landrace	Small red	FIPAH	Yorito	Yorito	Yoro
**65**	Olanchano Negro		x	x	x	Landrace	Black	CIAL San Jose de la Mora	San Jose de la Mora	Vallecillo	Francisco Morazán
**66**	Seda-Vallecillo		x	x	x	Landrace	Small red	CIAL San José de la Mora	San Jose de La Mora	Vallecillo	Francisco Morazán
**67**	Chirineño		x	x	x	Landrace	Small red	CIAL Chirinos	Chirinos	Cedros	Francisco Morazán
**68**	Roseño		x	x	x	Landrace	Small red	Adan Bustillo	La Fortuna	Victoria	Yoro
**70**	Negro Opalaca		x	x	x	Landrace	Black	NA	Monte Verde	San Francisco de Opalaca	Intibucá
**82**	Olanchano Rojo		x			Landrace	Small red	San Jose de la Mora	San Jose de la Mora	Vallecillo	Francisco Morazán

^1^ Genotypes exhibiting daylight sensitivity at Elora 2014. ^2^ Genotypes exhibiting uneven maturity at Elora 2014 and sent to Puerto Rico for seed increase in winter 2015. ^NA^ Information not available.

**Table 8:** Genotypes of PPB breeding category that were included in the HON panel tested at three field locations, 2014–15. The trials in which each genotype was included and whether the entry was SNP genotyped are indicated. Breeding category, market class, and origin details are provided where available.

									Seed Origin			
HON Entry	VARIETY	Elora ‘14	Yorito ‘14–’15	Elora ‘15	SNP Genotyped	Breeding Category	Market Class	Genealogy	Institution, Farmer, or Organization	Locality	Municipality	Department
**01**	Macuzalito	x	x	x	x	PPB (PPB)^3^	Small red	PPB-9911-44-5-13M, [Concha Rosada//SRC 1-1-18/SRC 1-12-1]	PIF/Zamorano, FIPAH, Honduras; 2004	Yorito	Yorito	Yoro
**13**	FPY-722-53 ^1^	x			x	PPB (PPB)	Small red	FPY-722-53, Tio Canela 75/ Estica	PIF/Zamorano, FIPAH, CIAL Santa Cruz	Santa Cruz	Yorito	Yoro
**14**	FPY-722-38	x	x	x	x	PPB (PPB)	Small red	FPY-722-38, Tio Canela 75/Estica	PIF/Zamorano, FIPAH, CIAL Santa Cruz	Santa Cruz	Yorito	Yoro
**15**	FPY-722-13 ^1^	x	x		x	PPB (PPB)	Small red	FPY-722-13, Tio Canela 75/Estica	PIF/Zamorano, FIPAH, CIAL Santa Cruz	Santa Cruz	Yorito	Yoro
**16**	FPY-724-43 ^1^	x	x	x	x	PPB (PPB)	Small red	FPY-724-43, Macuzalito/Estica	PIF/Zamorano, FIPAH, CIAL Santa Cruz	Santa Cruz	Yorito	Yoro
**18**	FPY-721-16 ^1^	x			x	PPB (PPB)	Small red	FPY-721-16, Amadeus 77/Estica	PIF/Zamorano, FIPAH, CIAL Santa Cruz	Yorito	Yorito	Yoro
**19**	FPY-722-41 ^2^	x	x	x	x	PPB (PPB)	Small red	FPY-722-41, Tio Canela 75/Estica	PIF/Zamorano, FIPAH, CIAL Santa Cruz, Amilcar Orellana	La Esperanza	Yorito	Yoro
**23**	PM2-Don Rey ^2^	x	x	x	x	PPB (PPB)	Small red	IBC-302-29, Carrizalito//Carrizalito/Paraísito	PIF/Zamorano, UPR, DICTA, ASOCIAL Vallecillo, Reinaldo Funez; 2014	San Isidro	Vallecillo	Francisco Morazán
**25**	FPV-921-4	x	x	x	x	PPB (PPB)	Small red	FPV-921-4, Vaina Rosada/Amadeus 77	CIAL San Isidro	San Isidro	Vallecillo	Francisco Morazán
**26**	Quebradeño	x	x		x	PPB (PPB)	Small red	IBC-307-7, [TC75//TC75/Cincuenteño]	CIAL Quebrada	Trinidad de Quebrada	Vallecillo	Francisco Morazán
**28**	FPV-921-61 ^1^	x				PPB (PPB)	Small red	FPV-921-61, Vaina Rosada/Amadeus 77	CIAL San Isidro	San Isidro	Vallecillo	Francisco Morazán
**31**	FPV-923-25 ^2^	x	x	x	x	PPB (PPB)	Small red	FPV-923-25, Vaina Rosada/Conan 33	CIAL San Isidro	San Isidro	Vallecillo	Francisco Morazán
**32**	FPV-923-21 ^1^	x			x	PPB (PPB)	Small red	FPV-923-21, Vaina Rosada/Conan 33	CIAL San Isidro	San Isidro	Vallecillo	Francisco Morazán
**33**	FPV 921-65 ^1^	x			x	PPB (PPB)	Small red	FPV 921-65, Vaina Rosada/Amadeus 77	CIAL San Isidro	San Isidro	Vallecillo	Francisco Morazán
**03**	Cedrón	x	x	x	x	PPB (PVS)^4^	Small red	EAP 9508-93, [Bribri//MD 30-37//PR 9177-214-1/Tio Canela 75]	PIF/Zamorano, FIPAH, Honduras; 2005	Yorito	Yorito	Yoro
**04**	Chepe	x	x	x	x	PPB (PVS)	Small red	703-SM15216-11-5	PIF/Zamorano, CIAT, FIPAH, Honduras; 2012	Yorito	Yorito	Yoro
**05**	Amilcar ^2^	x	x	x	x	PPB (PVS)	Small red	IBC 308-24, Amadeus 77//Amadeus 77/Cincuenteño	PIF/Zamorano, FIPAH, Honduras; 2012	Yorito	Yorito	Yoro
**10**	Marcelino ^2^	x	x	x	x	PPB (PVS)	Small red	EAP 9508-41, Bribri/MD 30-37//PR 9177-214-1/Tio Canela 75	PIF/Zamorano, FIPAH, Edy Hernandez; 2012	La Patastera	Yorito	Yoro
**12**	Esperanceño ^2^	x	x	x	x	PPB (PVS)	Small red	PR 0310-26-3-3, VAX 6	PIF/Zamorano, UPR, CIAT, FIPAH, Pablo Orellana; 2011	La Esperanza	Yorito	Yoro
**17**	Paisano PF	x	x	x	x	PPB (PVS)	Small red	MER-2212-28, Milenio/Amadeus 77	PIF/Zamorano, PRR, CIAL Palmicha Fatima; 2011	Palmichal Fatima	Siguatepeque	Comayagua
**20**	523-DFBS 15092-04-4	x	x	x	x	PPB (PVS)	Small red	523-DFBS 15092-04-4	PIF/Zamorano, CIAT, FIPAH	Yorito	Yorito	Yoro
**21**	519-DFBZ 15094-39-4 ^2^	x	x	x	x	PPB (PVS)	Small red	519-DFBZ 15094-39-4	PIF/Zamorano, CIAT, FIPAH	Yorito	Yorito	Yoro
**24**	Conan 33	x	x	x	x	PPB (PVS)	Small red	PRF-9659-25B-1, [EAP 9503/RS3//Bribri/MD 30-37////EAP 9503/RS3//A429/K2///V8025/XR 16492//APN83/CNC]	PIF/Zamorano, FIPAH, Asocial Vallecillo; 2005	Trinidad de Quebrada	Vallecillo	Francisco Morazán
**35**	San Jose	x	x	x	x	PPB (PVS)	Small red	X0-233-171-4, VAX 3	PIF/Zamorano, UPR, CIAT, FIPAH, CIAL San José; date NA	San Jose	Vallecillo	Francisco Morazán
**57**	Campechano	x	x	x	x	PPB (PVS)	Small red	SX14825-7-1	PIF/Zamorano, CIAT, PRR, ASOCIALAYO; 2012	La Buena Fe	Zacapa	Santa Bárbara
**58**	Don Kike	x	x	x	x	PPB (PVS)	Small red	MDSX14797-6-1	PIF/Zamorano, CIAT, PRR, ASOCIALAYO; 2012	La Buena Fe	Zacapa	Santa Bárbara
**72**	Arbolito Negro		x	x	x	PPB (PVS)	Black	SJC 729-89, Negro Vaina Blanca/PRF 9924-50N	PIF/Zamorano, PRR, ASOCIALAYO; 2013	La Buena Fe	Zacapa	Santa Bárbara
**74**	Rojo Delicia		x	x	x	PPB (PVS)	Small red	703-SM15216-11-4-VR	PIF/Zamorano, CIAT, PRR, ASOCIALAYO; 2015	La Buena Fe	Zacapa	Santa Bárbara
**75**	Don Cristóbal		x	x	x	PPB (PVS)	Small red	SRC1-12-1-8, [DOR476//XAN155/DOR364]	PIF/Zamorano, CIAT, PRR, CIAL Laguna Seca; 2015	Laguna Seca	Taulabe	Comayagua
**76**	Victoria		x	x	x	PPB (PVS)	Small red	SRS 56-3, [Amadeus77/SEA5]	PIF/Zamorano, PRR, CIAL Nueva Esperanza; 2015	Nueva Esperanza	Concepción Sur	Santa Bárbara
**78**	Nueva Esperanza		x	x	x	PPB (PVS)	Small red	DICZA 9801, UPR9606-2-2/MD 30-37	PIF/Zamorano, PRR, CIAL Nueva Esperanza; 2005	Nueva Esperanza	Concepción Sur	Santa Bárbara

^1^ Genotypes exhibiting daylight sensitivity at Elora 2014. ^2^ Genotypes exhibiting uneven maturity at Elora 2014 and sent to Puerto Rico for seed increase in winter 2015. ^3^ PPB are participatory plant bred varieties derived from a cross between a landrace and a breeding line; classified by FIPAH. ^4^ PVS are varieties derived from participatory varietal selection where breeding lines are selected through generations of testing by CIALs; classified by FIPAH. ^NA^ Information not available.

## Supplementary Materials

The following are available online at https://www.mdpi.com/2223-7747/9/9/1238/s1, **Table S1**: Candidate genes identified in regions of the *P. vulgaris* (2.0) genome where high nucleotide diversity (π) was discovered in landrace genotypes compared to PPB genotypes [5,32,33,35,37,38,39,46,63,67,111,112,113,114,115]. **Table S2**: Candidate genes identified in regions of the *P. vulgaris* (2.0) genome where SNPs with significantly high weighted FST values (>0.5) were found [40,42,43,44,46,116,117]. **Table S3**: F-test of fixed and Pearson’s χ^2^ test of random effects in the combined GLIMMIX mixed model analysis of the Honduran panel genotypes tested in multiple field locations in Ontario, Canada and Yorito, Honduras, 2014–2015. **Table S4**: F-test of fixed effect of genotype overall and by breeding history category, and the Pearson’s χ^2^ test of random effects in the GLIMMIX analysis of 63 genotypes tested at Elora, Ontario, Canada, 2014. **Table S5**: F-test of fixed effect of genotype overall and by breeding history category, and the Pearson’s χ^2^ test of random effects in the GLIMMIX analysis of 63 genotypes tested at Elora, Ontario, Canada, 2015. **Table S6**: F-test of fixed effect of genotype overall and by breeding history category, and the Pearson’s χ^2^ test of random effects in the GLIMMIX analysis of 63 genotypes tested at Yorito, Honduras, 2014–2015. **Table S7**: F-test of fixed effects and Pearson’s χ^2^ test of random effects in the GLIMMIX mixed-model analysis of leaf chlorophyll content (SPAD) at early vegetative (SPAD1) and reproductive (SPAD2) stages in the Honduran panel overall (Combined) and at Elora in 2014 and 2015. **Table S8**: Phenotypic (r_p_) correlations among %Ndfa and other traits estimated in the Honduran panel grown in three locations in 2014–2015. Values shown for traits within environments are significantly correlated at the 5% level. The number of genotypes analyzed in each correlation is presented in brackets for each significant correlation. **Figure S1**: Histograms of Nitrogen derived from the atmosphere (%) values of genotypes comprising the HON panel tested at three field locations from 2014–2015. A. Elora 2014, B. Elora 2015, and C. Yorito. Breeding history category averages with standard errors are presented, followed by individual genotype LSmeans with standard errors. North American check genotypes (CK; purple), Honduran conventional genotypes (CV; blue), landraces (LR; green), and PPB varieties (PB; red). Genotype numbers correspond to those listed in Table 6, Table 7 and Table 8. **Figure S2**: Histograms of carbon discrimination (Δ) values of genotypes comprising the HON panel tested at three field locations from 2014–2015. A. Elora 2014, B. Elora 2015, and C. Yorito. Breeding history category averages with standard errors are presented, followed by individual genotype LSmeans with standard errors. North American check genotypes (CK; purple), Honduran conventional genotypes (CV; blue), landraces (LR; green), and PPB varieties (PB; red). Genotype numbers correspond to those listed in Table 6, Table 7 and Table 8. **Figure S3**: Histograms of days to flowering of genotypes comprising the HON panel tested at two field locations from 2014–2015. A. Elora 2014, B. Elora 2015. Breeding history category averages with standard errors are presented, followed by individual genotype LSmeans with standard errors. North American check genotypes (CK; purple), Honduran conventional genotypes (CV; blue), landraces (LR; green), and PPB varieties (PB; red). Genotype numbers correspond to those listed in Table 6, Table 7 and Table 8. **Figure S4**: Histograms of days to maturity of genotypes comprising the HON panel tested at two field locations from 2014–2015. A. Elora 2014 and B. Elora 2015. Breeding history category averages with standard errors are presented, followed by individual genotype LSmeans with standard errors. North American check genotypes (CK; purple), Honduran conventional genotypes (CV; blue), landraces (LR; green), and PPB varieties (PB; red). Genotype numbers correspond to those listed in Table 6, Table 7 and Table 8. **Figure S5**: Histograms of yield (kg ha^−1^) values of genotypes comprising the HON panel tested at three field locations from 2014–2015. A. Elora 2014, B. Elora 2015, and C. Yorito. Breeding history category averages with standard errors are presented, followed by individual genotype LSmeans with standard errors. North American check genotypes (CK; purple), Honduran conventional genotypes (CV; blue), landraces (LR; green), and PPB varieties (PB; red). Genotype numbers correspond to those listed in Table 6, Table 7 and Table 8. **Data Repository files**: Phenotypic, SNP, and genetic diversity datasets. Available at https://doi.org/10.5683/SP2/DJB7VY
