# Supplementary material for: Genetic Diversity, Nitrogen Fixation, and Water Use Efficiency in a Panel of Honduran Common Bean (Phaseolus vulgaris L.) Landraces and Modern Genotypes
Source: Plants (Basel). 2020 Sep 19;9(9):1238. doi: 10.3390/plants9091238 (PMC7569834; doi:10.3390/plants9091238)
Supplement: Supplementary file 1 [file plants-09-01238-s001.zip › Figures S1-S5 - Copy.pdf]

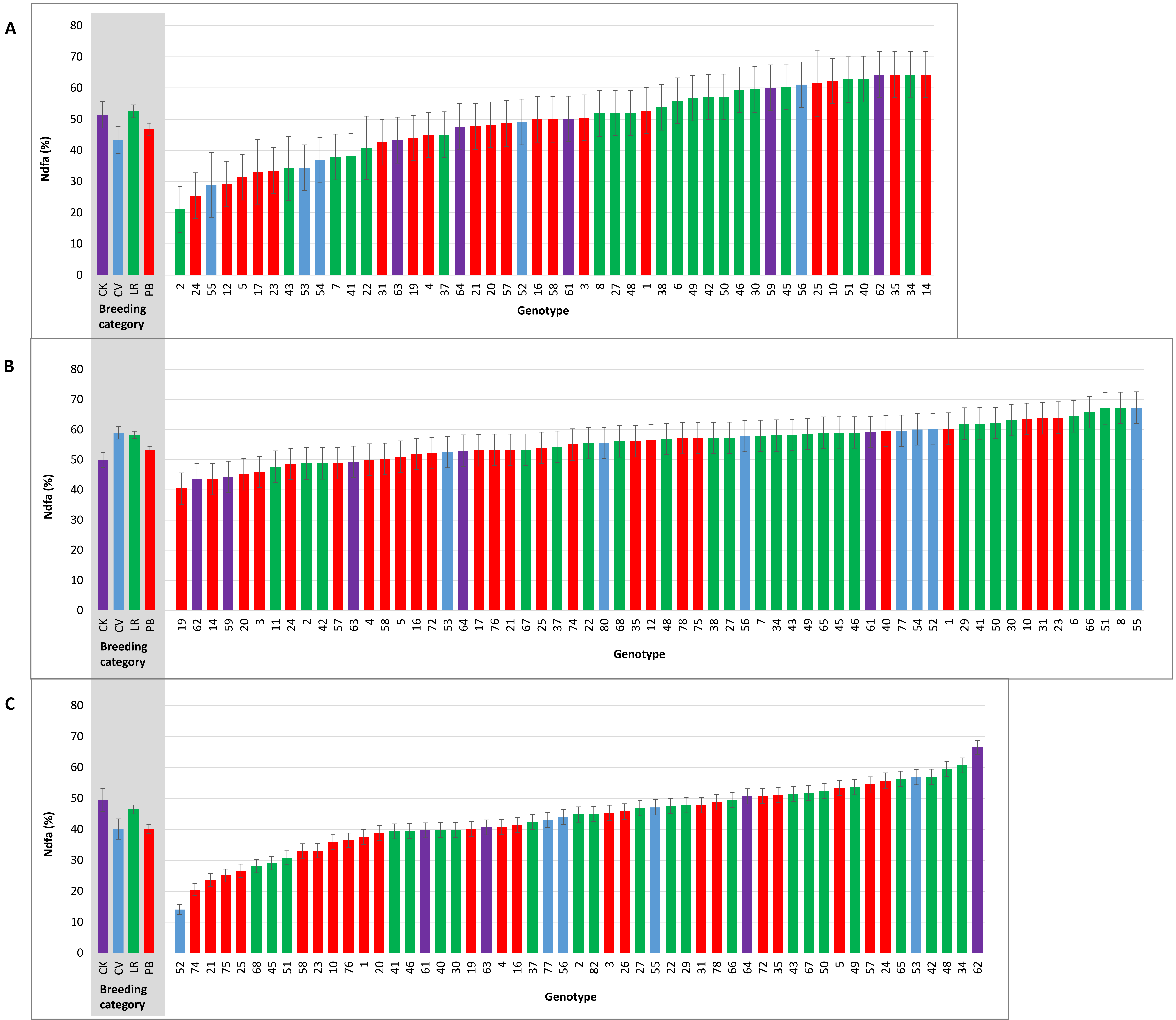
A
Ndfa (%)
0 10 20 30 40 50 60 70 80
CK CV LR PB
Breeding category
2 24 55 12 5 17 23 43 53 54 7 41 22 31 63 19 4 37 64 21 20 57 52 16 58 61 3 8 27 48 1 38 6 49 42 50 46 30 59 45 56 25 10 51 40 62 35 34 14
Genotype
B
Ndfa (%)
0 10 20 30 40 50 60 70 80
CK CV LR PB
Breeding category
19 62 14 59 20 3 11 24 2 42 57 63 4 58 5 16 72 53 64 17 76 21 67 25 37 74 22 80 68 35 12 48 78 75 38 27 56 7 34 43 49 65 45 46 61 40 77 54 52 1 29 41 50 30 10 31 23 6 66 51 8 55
Genotype
C
Ndfa (%)
0 10 20 30 40 50 60 70 80
CK CV LR PB
Breeding category
52 74 21 75 25 68 45 51 58 23 10 76 1 20 41 46 61 40 30 19 63 4 16 37 77 56 2 82 3 26 27 55 22 29 31 78 66 64 72 35 43 67 50 5 49 57 24 65 53 42 48 34 62
Genotype

Figure S1. Histograms of Nitrogen derived from the atmosphere (%) values of genotypes comprising the HON panel tested at three field locations from 2014-2015. **A**. Elora 2014, **B**. Elora 2015 and **C**. Yorito. Breeding history category averages with standard errors are presented, followed by individual genotype LSmeans with standard errors. North American check genotypes (CK; purple), Honduran conventional genotypes (CV; blue), landraces (LR; green), and PPB varieties (PB; red). Genotype numbers correspond to those listed in Tables 6-8.

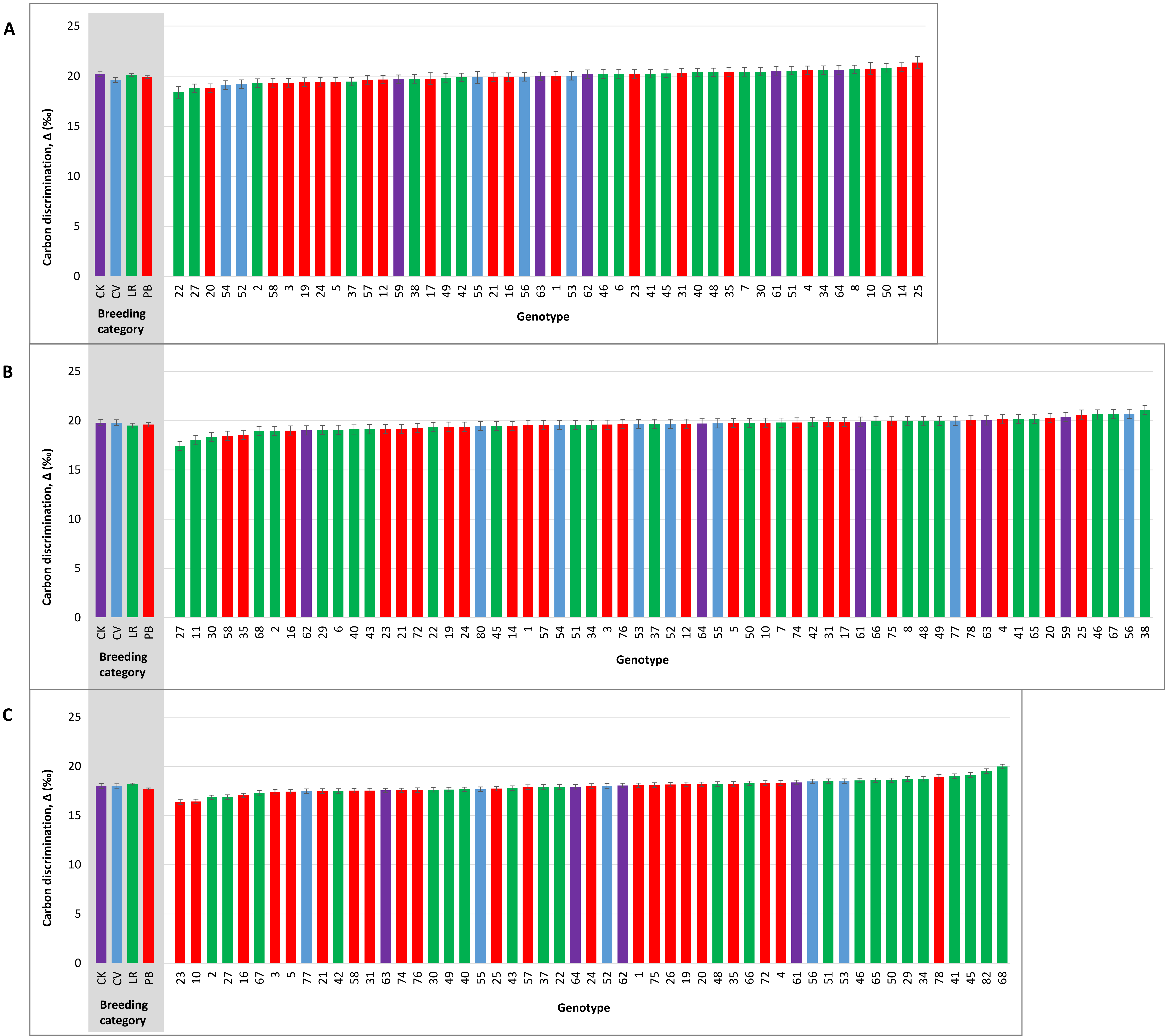
A
B
C
Carbon discrimination, Δ (‰)
0
5
10
15
20
25
CK CV LR PB
Breeding category
Genotype
22 27 20 54 52 2 58 3 19 24 5 37 57 12 59 38 17 49 42 55 21 16 56 63 1 53 62 46 6 23 41 45 31 40 48 35 7 30 61 51 4 34 64 8 10 50 14 25
27 11 30 58 35 68 2 16 62 29 6 40 43 23 21 72 22 19 24 80 45 14 1 57 54 51 34 3 76 53 37 52 12 64 55 5 50 10 7 74 42 31 17 61 66 75 8 48 49 77 78 63 4 41 65 20 59 25 46 67 56 38
23 10 2 27 16 67 3 5 77 21 42 58 31 63 74 76 30 49 40 55 25 43 57 37 22 64 24 52 62 1 75 26 19 20 48 35 66 72 4 61 56 51 53 46 65 50 29 34 78 41 45 82 68

Figure S2. Histograms of carbon discrimination (Δ) values of genotypes comprising the HON panel tested at three field locations from 2014-2015. **A**. Elora 2014, **B**. Elora 2015 and **C**. Yorito. Breeding history category averages with standard errors are presented, followed by individual genotype LSmeans with standard errors. North American check genotypes (CK; purple), Honduran conventional genotypes (CV; blue), landraces (LR; green), and PPB varieties (PB; red). Genotype numbers correspond to those listed in Tables 6-8.

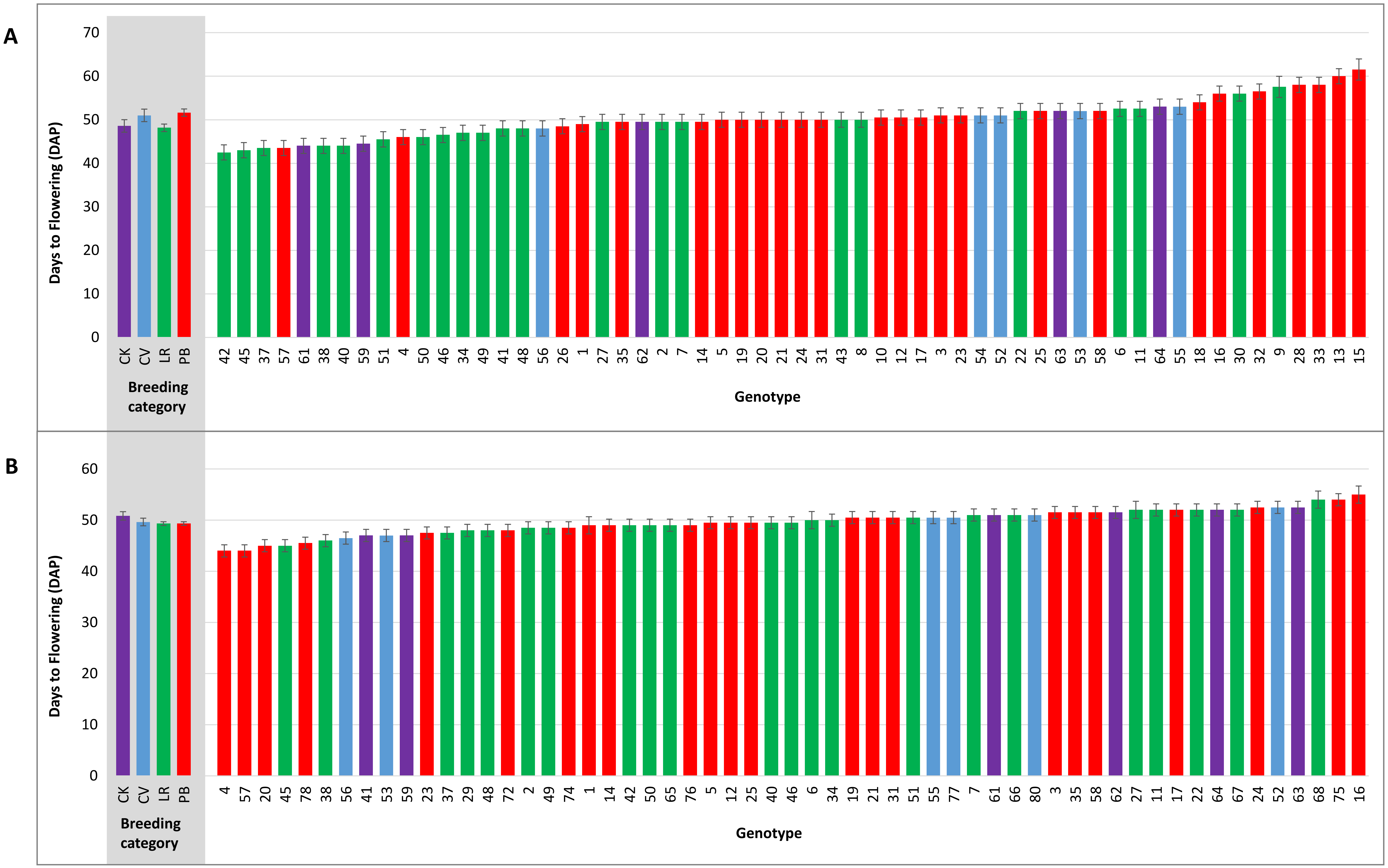
A
Days to Flowering (DAP)
Breeding category
Genotype
CK CV LR PB
42 45 37 57 61 38 40 59 51 4 50 46 34 49 41 48 56 26 1 27 35 62 2 7 14 5 19 20 21 24 31 43 8 10 12 17 3 23 54 52 22 25 63 53 58 6 11 64 55 18 16 30 32 9 28 33 13 15
B
Days to Flowering (DAP)
Breeding category
Genotype
CK CV LR PB
4 57 20 45 78 38 56 41 53 59 23 37 29 48 72 2 49 74 1 14 42 50 65 76 5 12 25 40 46 6 34 19 21 31 51 55 77 7 61 66 80 3 35 58 62 27 11 17 22 64 67 24 52 63 68 75 16

Figure S3. Histograms of days to flowering of genotypes comprising the HON panel tested at two field locations from 2014-2015. **A**. Elora 2014, **B**. Elora 2015. Breeding history category averages with standard errors are presented, followed by individual genotype LSmeans with standard errors. North American check genotypes (CK; purple), Honduran conventional genotypes (CV; blue), landraces (LR; green), and PPB varieties (PB; red). Genotype numbers correspond to those listed in Tables 6-8.

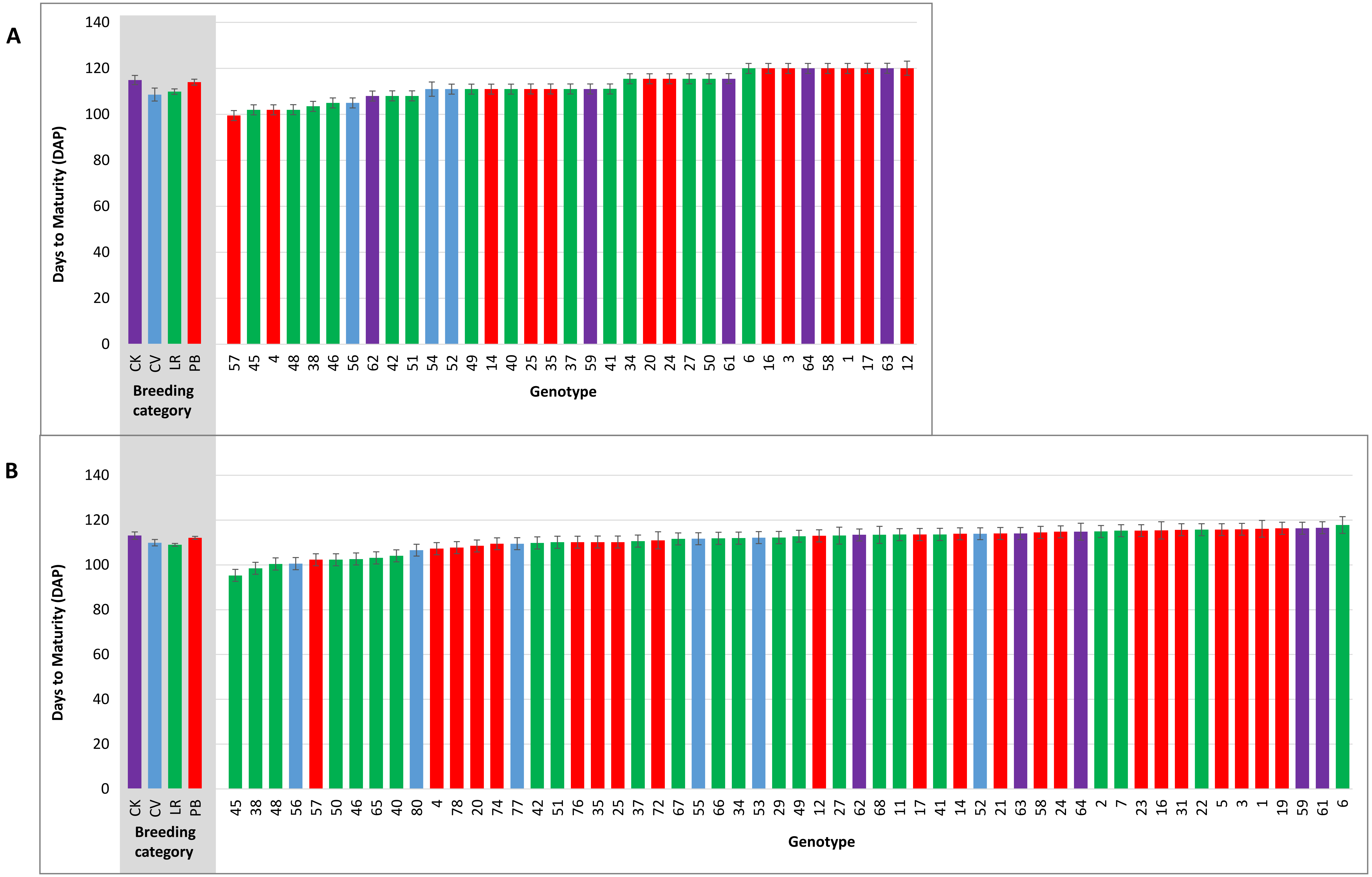
A
B
Days to Maturity (DAP)
140
120
100
80
60
40
20
0
CK
CV
LR
PB
Breeding category
Genotype
57 45 4 48 38 46 56 62 42 51 54 52 49 14 40 25 35 37 59 41 34 20 24 27 50 61 6 16 3 64 58 1 17 63 12
45 38 48 56 57 50 46 65 40 80 4 78 20 74 77 42 51 76 35 25 37 72 67 55 66 34 53 29 49 12 27 62 68 11 17 41 14 52 21 63 58 24 64 2 7 23 16 31 22 5 3 1 19 59 61 6

Figure S4. Histograms of days to maturity of genotypes comprising the HON panel tested at two field locations from 2014-2015. **A**. Elora 2014, **B**. Elora 2015. Breeding history category averages with standard errors are presented, followed by individual genotype LSmeans with standard errors. North American check genotypes (CK; purple), Honduran conventional genotypes (CV; blue), landraces (LR; green), and PPB varieties (PB; red). Genotype numbers correspond to those listed in Tables 6-8.

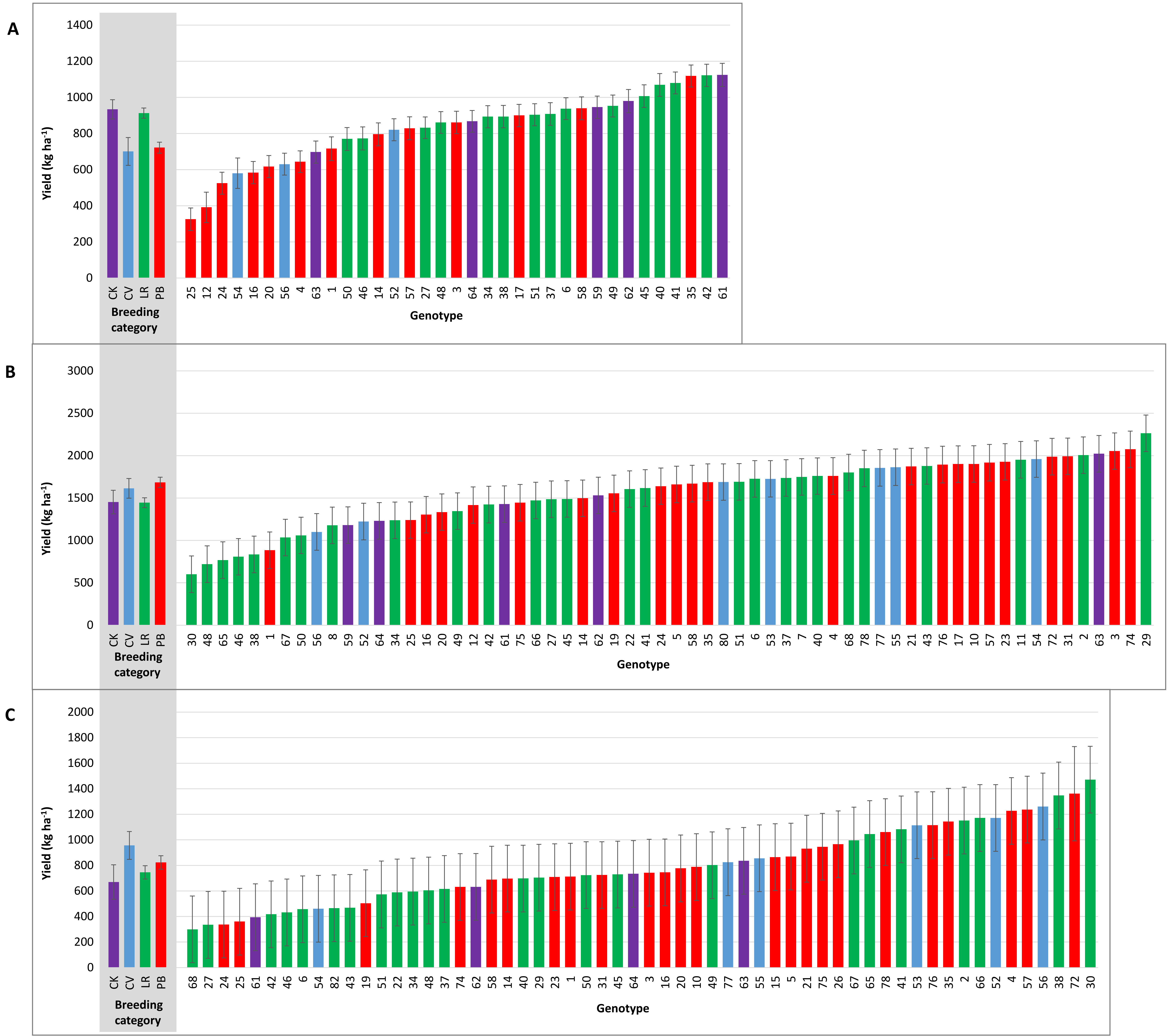
A
1400
1200
1000
800
600
400
200
0
Yield (kg ha-1)
CK CV LR PB
Breeding category
25 12 24 54 16 20 56 4 63 1 50 46 14 52 57 27 48 3 64 34 38 17 51 37 6 58 59 49 62 45 40 41 35 42 61
Genotype
B
3000
2500
2000
1500
1000
500
0
Yield (kg ha-1)
CK CV LR PB
Breeding category
30 48 65 46 38 1 67 50 56 8 59 52 64 34 25 16 20 49 12 42 61 75 66 27 45 14 62 19 22 41 24 5 58 35 80 51 6 53 37 7 40 4 68 78 77 55 21 43 76 17 10 57 23 11 54 72 31 2 63 3 74 29
Genotype
C
2000
1800
1600
1400
1200
1000
800
600
400
200
0
Yield (kg ha-1)
CK CV LR PB
Breeding category
68 27 24 25 61 42 46 6 54 82 43 19 51 22 34 48 37 74 62 58 14 40 29 23 1 50 31 45 64 3 16 20 10 49 77 63 55 15 5 21 75 26 67 65 78 41 53 76 35 2 66 52 4 57 56 38 72 30
Genotype

Figure S5. Histograms of yield (kg ha^{-1}) values of genotypes comprising the HON panel tested at three field locations from 2014-2015. **A**. Elora 2014, **B**. Elora 2015 and **C**. Yorito. Breeding history category averages with standard errors are presented, followed by individual genotype LSmeans with standard errors. North American check genotypes (CK; purple), Honduran conventional genotypes (CV; blue), landraces (LR; green), and PPB varieties (PB; red). Genotype numbers correspond to those listed in Tables 6-8.
