# Supplementary material for: Genetic Diversity, Nitrogen Fixation, and Water Use Efficiency in a Panel of Honduran Common Bean (Phaseolus vulgaris L.) Landraces and Modern Genotypes
Source: Plants (Basel). 2020 Sep 19;9(9):1238. doi: 10.3390/plants9091238 (PMC7569834; doi:10.3390/plants9091238)
Supplement: Supplementary file 1 [file plants-09-01238-s001.zip › Supplementary tables_Sept9.docx]

**Table S1.** Candidate genes identified in regions of the *P. vulgaris* (2.0) genome where high nucleotide diversity (π) was discovered in landrace genotypes compared to PPB genotypes. Candidate genes within or overlapping with these regions are listed with annotation from JBrowse (<https://legumeinfo.org/genomes/jbrowse/>). (Chr – Chromosome)

|  | **Region of high diversity** | | **π value** | |  |  |  |  |
| --- | --- | --- | --- | --- | --- | --- | --- | --- |
| **Chr** | **Start (Mbp)** | **End (Mbp)** | **Landrace** | **PPB** | **Total number of genes per region** | **Middle American gene pool domestication genes*** | **QTL and genes identified in other studies** | **Description of QTL and genes identified in other studies** |
| **1** | 23.0 | 24.0 | 5.4997E-07 | 1.3107E-07 | 2 | **Phvul.001G100800** | **Phvul.001G108101** (23,191,560..23,195,119) MATE efflux family protein | QTL (23.2Mbp) associated with days to flowering and hundred seed weight in Middle American diversity panel (Wilker et al, unpublished). |
| **1** | 40.0 | 41.0 | 7.7102E-07 | 1.8305E-07 | 4 | **Phvul.001G148100 Phvul.001G150700 Phvul.001G150800 Phvul.001G151700** |  |  |
| **1** | 41.0 | 42.0 | 1.2422E-06 | 2.4915E-07 | 4 | **Phvul.001G156000 Phvul.001G156200 Phvul.001G156400 Phvul.001G160000** |  |  |
| **1** | 42.0 | 43.0 | 1.2226E-06 | 3.9831E-07 | 1 |  | **Phvul.001G167200** (42,856,008..42,859,005) RNA polymerase-associated protein RTF1 homolog [Glycine max] | QTL (42.9Mbp) associated with growth habit in Middle American diversity panel (Moghaddam et al, 2016). |
| **1** | 47.0 | 48.0 | 8.6039E-07 | 9.661E-08 | 3 | **Phvul.001G210000 Phvul.001G210300 Phvul.001G212400** |  |  |
| **2** | 4.0 | 5.0 | 1.9461E-06 | 6.5085E-07 | 0 |  |  |  |
| **2** | 22.0 | 23.0 | 1.7113E-06 | 1.6441E-07 | 8 | **Phvul.002G109400** | **Phvul.002G106500** (21,498,604..21,509,530) vacuolar sorting protein 35 | Genes underlying QTL (22.8Mbp) associated with %N in the Middle American diversity panel (Wilker et al, unpublished). GTPase gene (Phvul.002G106600), Flores et al, 2018. |
|  |  |  |  |  |  |  | **Phvul.002G106600** (21,510,899..21,513,855) RHO family GTPase |  |
|  |  |  |  |  |  |  | **Phvul.002G106700** (21,516,894..21,520,358) dehydroquinate synthase |  |
|  |  |  |  |  |  |  | **Phvul.002G106800** (21,521,806..21,525,244) Myb/SANT-like DNA-binding domain protein |  |
|  |  |  |  |  |  |  | **Phvul.002G106900** (21,526,081..21,530,585) uncharacterized protein |  |
|  |  |  |  |  |  |  | **Phvul.002G107000** (21,531,223..21,533,198) peroxidase |  |
|  |  |  |  |  |  |  | **Phvul.002G107100** (21,585,247..21,592,897) histidine kinase |  |
| **2** | 32.0 | 33.0 | 5.5262E-07 | 1.2203E-07 | 5 | **Phvul.002G172300 Phvul.002G172500 Phvul.002G172600 Phvul.002G174500 Phvul.002G174600** |  |  |
| **2** | 48.0 | 49.0 | 5.9207E-06 | 1.6232E-06 | 18 | **Phvul.002G320800** | **Phvul.002G320000** (47,985,266..47,989,823) Ubiquitin-conjugating enzyme E2 | Genes underlying QTL (48.6Mbp) associated with days to flowering in Middle American diversity panel (Wilker et al, unpublished). Calmodulin-like gene (Phvul.002G320800), Mohanta et al, 2017. |
|  |  |  |  |  |  |  | **Phvul.002G320100** (47,991,022..47,997,910) Histidyl-tRNA synthetase |  |
|  |  |  |  |  |  |  | **Phvul.002G320200** (47,999,699..48,001,370) Ribosomal protein S19 family protein |  |
|  |  |  |  |  |  |  | **Phvul.002G320300** (48,004,702..48,009,035) XAP5 family protein |  |
|  |  |  |  |  |  |  | **Phvul.002G320400** (48,014,427..48,017,026) XAP5 family protein (circadian clock regulator) |  |
|  |  |  |  |  |  |  | **Phvul.002G320500** (48,021,216..48,022,697) diphosphoinositol polyphosphate phosphohydrolase |  |
|  |  |  |  |  |  |  | **Phvul.002G320600** (48,029,075..48,030,117) unknown protein |  |
|  |  |  |  |  |  |  | **Phvul.002G320700** (48,030,317..48,033,276) uncharacterized putative methyltransferase |  |
|  |  |  |  |  |  |  | **Phvul.002G320800** (48,035,445..48,036,011) probable calcium-binding protein CML25-like |  |
|  |  |  |  |  |  |  | **Phvul.002G320900** (48,037,219..48,041,606) ubiquitin fusion degradation 1 |  |
|  |  |  |  |  |  |  | **Phvul.002G321000** (48,043,674..48,047,394) Ankyrin repeat family protein |  |
|  |  |  |  |  |  |  | **Phvul.002G321100** (48,047,868..48,055,921) protein VAC14 homolog [Glycine max] |  |
|  |  |  |  |  |  |  | **Phvul.002G321200** (48,057,723..48,067,814) nuclear pore complex protein Nup155-like [Glycine max] |  |
|  |  |  |  |  |  |  | **Phvul.002G321300** (48,070,793..48,074,236) RING-H2 finger protein [Glycine max] |  |
|  |  |  |  |  |  |  | **Phvul.002G321400** (48,077,719..48,079,912) Ribosomal S5 family protein |  |
|  |  |  |  |  |  |  | **Phvul.002G321500** (48,082,538..48,087,118) microtubule-associated proteins 70-2 |  |
|  |  |  |  |  |  |  | **Phvul.002G321600** (48,087,876..48,089,363) Mitochondrial transcription termination factor family protein |  |
|  |  |  |  |  |  |  | **Phvul.002G321700** (48,091,104..48,099,000) DYNAMIN-like 1C vacuolar sorting protein |  |
| **3** | 34.0 | 35.0 | 6.7266E-07 | 1.9661E-07 | 8 | **Phvul.003G144500 Phvul.003G145300 Phvul.003G145400 Phvul.003G145700 Phvul.003G146000 Phvul.003G146600 Phvul.003G146800 Phvul.003G148900** |  |  |
| **3** | 48.0 | 49.0 | 1.1682E-06 | 1.887E-07 | 8 | **Phvul.003G253300 Phvul.003G254000 Phvul.003G254800 Phvul.003G254900 Phvul.003G258800 Phvul.003G259500 Phvul.003G259700 Phvul.003G259800** |  |  |
| **4** | 12.0 | 13.0 | 1.5299E-06 | 2.6215E-07 | 0 |  |  |  |
| **4** | 17.0 | 18.0 | 4.0455E-07 | 6.5537E-08 | 0 |  |  |  |
| **4** | 38.0 | 39.0 | 4.7435E-07 | 9.661E-08 | 6 | **Phvul.004G116300 Phvul.004G116700 Phvul.004G117700 Phvul.004G118100 Phvul.004G119700 Phvul.004G120000** |  |  |
| **4** | 40.0 | 41.0 | 4.3152E-07 | 1.2655E-07 | 0 |  |  |  |
| **5** | 24.0 | 25.0 | 3.5537E-07 | 6.5537E-08 | 1 | **Phvul.005G089100** |  |  |
| **5** | 25.0 | 26.0 | 2.1047E-06 | 1.9209E-07 | 0 |  |  |  |
| **5** | 27.0 | 28.0 | 7.4564E-07 | 1.3107E-07 | 1 | **Phvul.005G092300** |  |  |
| **5** | 32.0 | 33.0 | 1.1592E-06 | 3.2768E-07 | 11 | **Phvul.005G109700 Phvul.005G110000** | **Phvul.005G104700** (31,137,049..31,138,536) 60S acidic ribosomal protein family | Genes underlying QTL (32.5Mbp) associated with hundred seed weight in the Middle American diversity panel (Wilker et al, unpublished). Gene (Phvul.005G105200) involved in phosophorus use efficiency, da Silva et al, 2019, and involved in abiotic stress, Konzen et al, 2019. |
|  |  |  |  |  |  |  | **Phvul.005G104800** (31,142,070..31,143,525) Clathrin light chain protein |  |
|  |  |  |  |  |  |  | **Phvul.005G104900** (31,158,921..31,164,749) pre-mRNA splicing factor-related |  |
|  |  |  |  |  |  |  | **Phvul.005G105000** (31,165,180..31,167,685) sugar porter (SP) family MFS transporter |  |
|  |  |  |  |  |  |  | **Phvul.005G105100** (31,190,419..31,191,384) hypothetical protein |  |
|  |  |  |  |  |  |  | **Phvul.005G105200** (31,203,406..31,204,629) ethylene responsive element binding factor 1 |  |
|  |  |  |  |  |  |  | **Phvul.005G105300** (31,226,716..31,230,233) Regulator of Vps4 activity in the MVB pathway protein |  |
|  |  |  |  |  |  |  | **Phvul.005G105400** (31,234,261..31,235,004) Late embryogenesis abundant (LEA) hydroxyproline-rich glycoprotein family |  |
|  |  |  |  |  |  |  | **Phvul.005G105500** (31,235,613..31,236,357) hypothetical protein |  |
| **6** | 9.0 | 10.0 | 1.2057E-06 | 6.5537E-08 | 7 | **Phvul.006G018100 Phvul.006G018700 Phvul.006G019300 Phvul.006G019500 Phvul.006G019600 Phvul.006G019700 Phvul.006G019900** | **Phvul.006G019300** | Gene (Phvul.006G019300) associated with phosphorus use efficiency, da Silva et al, 2019. |
| **6** | 13.0 | 14.0 | 1.018E-06 | 1.3107E-07 | 14 | **Phvul.006G032500 Phvul.006G033700 Phvul.006G034400** | **Phvul.006G039100** (14,865,891..14,874,127) DEAD-box ATP-dependent RNA helicase | Genes underlying QTL (13.9Mbp) associated with days to flowering in the Middle American diversity panel (Wilker et al, unpublished). Gene (Phvul.006G038100) involved in bean common mosaic virus response, Martin et al, 2016. |
|  |  |  |  |  |  |  | **Phvul.006G039000** (14,862,108..14,863,739) LRR and NB-ARC domain disease resistance protein |  |
|  |  |  |  |  |  |  | **Phvul.006G038800** (14,854,569..14,857,149) uncharacterized protein LOC100777222 isoform X2 [Glycine max] |  |
|  |  |  |  |  |  |  | **Phvul.006G038900** (14,855,007..14,855,339) uncharacterized protein LOC100803137 [Glycine max] |  |
|  |  |  |  |  |  |  | **Phvul.006G038700** (14,849,231..14,850,850) LRR and NB-ARC domain disease resistance protein |  |
|  |  |  |  |  |  |  | **Phvul.006G038600** (14,843,654..14,846,550) LHCP translocation defect protein, putative |  |
|  |  |  |  |  |  |  | **Phvul.006G038500** (14,834,906..14,839,087) serine/arginine repetitive matrix protein 2-like [Glycine max] |  |
|  |  |  |  |  |  |  | **Phvul.006G038400** (14,825,494..14,828,231) septum-promoting GTP-binding protein 1-like [Glycine max] |  |
|  |  |  |  |  |  |  | **Phvul.006G038300** (14,818,508..14,823,231) hypothetical protein |  |
|  |  |  |  |  |  |  | **Phvul.006G038200** (14,808,094..14,816,761) Nucleotide/sugar transporter family protein |  |
|  |  |  |  |  |  |  | **Phvul.006G038100** (14,805,826..14,807,523) disease resistance protein (CC-NBS-LRR class) family protein |  |
| **6** | 15.0 | 16.0 | 4.8229E-07 | 1.5537E-07 | 6 | **Phvul.006G040400 Phvul.006G040800 Phvul.006G043100 Phvul.006G044500 Phvul.006G046100 Phvul.006G046400** |  |  |
| **7** | 8.0 | 9.0 | 4.3205E-07 | 9.661E-08 | 1 | **Phvul.007G011700** |  |  |
| **7** | 20.0 | 21.0 | 5.2723E-07 | 6.5537E-08 | 1 | **Phvul.007G120500** |  |  |
| **7** | 26.0 | 27.0 | 3.9831E-06 | 1.0124E-06 | 0 |  |  |  |
| **7** | 35.0 | 36.0 | 3.3644E-06 | 7.3785E-07 | 9 |  | **Phvul.007G232000** (47,185,070..47,194,601) 3-isopropylmalate dehydratase, large subunit | Genes underlying QTL (35.6Mbp) associated with days to maturity in the Middle American diversity panel (Wilker et al, unpublished). |
|  |  |  |  |  |  |  | **Phvul.007G232100** (47,195,159..47,196,475) Calcium-dependent lipid-binding (CaLB domain) family protein |  |
|  |  |  |  |  |  |  | **Phvul.007G232200** (47,197,987..47,204,888) ras GTPase-activating protein-binding protein 2-like isoform X1 [Glycine max] |  |
|  |  |  |  |  |  |  | **Phvul.007G232300** (47,208,285..47,211,396) cation/H+ exchanger |  |
|  |  |  |  |  |  |  | **Phvul.007G232400** (47,219,495..47,223,009) cation/H+ exchanger |  |
|  |  |  |  |  |  |  | **Phvul.007G232500** (47,225,864..47,227,706) Pentatricopeptide repeat (PPR-like) superfamily protein |  |
|  |  |  |  |  |  |  | **Phvul.007G232600** (47,242,795..47,243,289) small acidic protein 1-like [Glycine max] |  |
|  |  |  |  |  |  |  | **Phvul.007G232800** (47,262,900..47,289,477) lipase-like isoform X2 [Glycine max] |  |
|  |  |  |  |  |  |  | **Phvul.007G232900** (47,293,727..47,320,751) DNA mismatch repair MUTS family protein |  |
| **7** | 39.0 | 40.0 | 2.4331E-06 | 3.3164E-07 | 8 | **Phvul.007G164500 Phvul.007G165900 Phvul.007G166100 Phvul.007G166200 Phvul.007G166300 Phvul.007G166700 Phvul.007G166800 Phvul.007G166900** |  |  |
| **8** | 11.0 | 12.0 | 5.4389E-06 | 1.7424E-06 | 1 | **Phvul.008G103600** |  |  |
| **8** | 15.0 | 16.0 | 1.6298E-06 | 5.322E-07 | 2 | **Phvul.008G121000 Phvul.008G121100** |  |  |
| **8** | 18.0 | 19.0 | 1.2977E-06 | 3.9774E-07 | 1 | **Phvul.008G125300** |  |  |
| **8** | 23.0 | 24.0 | 5.2723E-07 | 6.5537E-08 | 0 |  |  |  |
| **8** | 29.0 | 30.0 | 1.3453E-06 | 3.322E-07 | 0 |  |  |  |
| **8** | 38.0 | 39.0 | 3.2893E-07 | 3.3333E-08 | 0 |  |  |  |
| **8** | 41.0 | 42.0 | 7.6203E-07 | 2.2599E-07 | 2 | **Phvul.008G162600 Phvul.008G162700** |  |  |
| **8** | 44.0 | 45.0 | 1.3533E-06 | 1.6215E-07 | 0 |  |  |  |
| **8** | 45.0 | 46.0 | 9.1486E-07 | 2.8983E-07 | 0 |  |  |  |
| **8** | 51.0 | 52.0 | 2.8593E-06 | 6.3333E-07 | 6 | **Phvul.008G202200 Phvul.008G202300 Phvul.008G202400 Phvul.008G204800 Phvul.008G208700 Phvul.008G208800** |  |  |
| **8** | 52.0 | 53.0 | 1.211E-06 | 9.661E-08 | 12 | **Phvul.008G211100 Phvul.008G211200 Phvul.008G211600 Phvul.008G213000 Phvul.008G214400 Phvul.008G214600 Phvul.008G216100 Phvul.008G216200 Phvul.008G216300 Phvul.008G216600 Phvul.008G216700 Phvul.008G217100** | **Phvul.008G211200 Phvul.008G214400 Phvul.008G217100** | Genes (Phvul.008G211200, Phvul.008G214400) involved in bean rust response, Ayyappan et al, 2015. Gene (Phvul.008G217100) involved in nodule ontogeny, Guillen et al, 2013. |
| **8** | 57.0 | 58.0 | 3.4532E-07 | 6.5537E-08 | 0 |  |  |  |
| **9** | 16.0 | 17.0 | 7.8107E-07 | 1.5989E-07 | 0 |  |  |  |
| **9** | 18.0 | 19.0 | 9.0376E-07 | 3.3333E-08 | 3 | **Phvul.009G123600 Phvul.009G123900 Phvul.009G124700** |  |  |
| **9** | 21.0 | 22.0 | 1.9434E-06 | 4.9944E-07 | 0 |  |  |  |
| **10** | 17.0 | 18.0 | 2.9667E-07 | 9.322E-08 | 0 |  |  |  |
| **10** | 33.0 | 34.0 | 2.2597E-06 | 7.4124E-07 | 0 |  |  |  |
| **10** | 35.0 | 36.0 | 9.7832E-07 | 2.6215E-07 | 2 | **Phvul.010G101800 Phvul.010G102300** | **Phvul.010G101800 Phvul.010G102300** | Gene (Phvul.010G101800) described in bean metabolomics study, Perez de Souza et al, 2019. NF-Y family gene (Phvul.010G102300), Ripodas et al, 2014. |
| **10** | 37.0 | 38.0 | 1.0661E-06 | 1.9435E-07 | 7 | **Phvul.010G110900 Phvul.010G112300 Phvul.010G112400 Phvul.010G112500 Phvul.010G112600 Phvul.010G112800 Phvul.010G112900** | **Phvul.010G112400** | Gene (Phvul.010G112400) involved in Pseudomonas response, Gonzalez et al, 2017. |
| **10** | 39.0 | 40.0 | 2.9995E-06 | 7.339E-07 | 1 | **Phvul.010G121500** | **Phvul.010G121500** | Gene (Phvul.010G121500) involved in phosphorus use efficiency, da Silva et al, 2019. |
| **11** | 2.0 | 3.0 | 8.7414E-07 | 1.9322E-07 | 3 | **Phvul.011G026200 Phvul.011G026300 Phvul.011G026400** | **Phvul.011G026300** | Gene (Phvul.011G026300) involved in phosophorus use efficiency, da Silva et al, 2019. |
| **11** | 7.0 | 8.0 | 7.9958E-07 | 1.5537E-07 | 23 | **Phvul.011G077200 Phvul.011G077300 Phvul.011G078400 Phvul.011G078600 Phvul.011G078700 Phvul.011G078800 Phvul.011G079000 Phvul.011G079300 Phvul.011G079500 Phvul.011G079600 Phvul.011G079900 Phvul.011G080900 Phvul.011G081200 Phvul.011G081400 Phvul.011G081500 Phvul.011G081600 Phvul.011G081700 Phvul.011G081800 Phvul.011G082100 Phvul.011G082400 Phvul.011G082700 Phvul.011G083100 Phvul.011G083200** | **Phvul.011G079300** | Gene (Phvul.011G079300) involved in drought response under AM symbiosis, Recchia et al, 2018. |
| **11** | 10.0 | 11.0 | 1.3749E-06 | 3.0057E-07 | 7 | **Phvul.011G098500 Phvul.011G099300 Phvul.011G099700 Phvul.011G099900 Phvul.011G100000 Phvul.011G100800 Phvul.011G101000** |  |  |
| **11** | 37.0 | 38.0 | 2.6367E-06 | 7.8418E-07 | 1 | **Phvul.011G144600** |  |  |
| **11** | 40.0 | 41.0 | 9.0693E-07 | 3.0057E-07 | 3 |  | **Phvul.011G144600** (37,051,493..37,051,901) DEAD-box ATP-dependent RNA helicase | Genes underlying QTL (40.3Mbp) associated with days to maturity in the Middle American diversity panel (Wilker et al, unpublished). |
|  |  |  |  |  |  |  | **Phvul.011G144500** (37,048,250..37,049,231) UPF0481 protein At3g47200-like [Glycine max] |  |
|  |  |  |  |  |  |  | **Phvul.011G144700** (37,053,630..37,055,280) DEAD-box ATP-dependent RNA helicase 39-like [Glycine max] |  |
| **11** | 45.0 | 46.0 | 7.8477E-06 | 9.8023E-07 | 9 |  | **Phvul.011G175900** (45,068,805..45,071,512) putative ribonuclease H protein At1g65750-like [Glycine max] | Genes underlying QTL (45.1Mbp) associated with shoot biomass (Kamfwa et al, 2019; Heilig et al, 2017). |
|  |  |  |  |  |  |  | **Phvul.011G176000** (45,075,074..45,076,054) RNA recognition motif, a.k.a. RRM, RBD protein |  |
|  |  |  |  |  |  |  | **Phvul.011G176200** (45,088,313..45,089,882) salicylic acid carboxyl methyltransferase |  |
|  |  |  |  |  |  |  | **Phvul.011G176300** (45,106,776..45,110,315) receptor kinase 3 |  |
|  |  |  |  |  |  |  | **Phvul.011G176400** (45,118,181..45,121,444) Protein kinase superfamily protein |  |
|  |  |  |  |  |  |  | **Phvul.011G176500** (45,126,787..45,131,817) WD repeat-containing protein 5-like [Glycine max] |  |
|  |  |  |  |  |  |  | **Phvul.011G176600** (45,134,053..45,146,869) Transducin/WD40 repeat-like superfamily protein |  |
|  |  |  |  |  |  |  | **Phvul.011G176700** (45,151,411..45,155,628) Protein kinase superfamily protein |  |
|  |  |  |  |  |  |  | **Phvul.011G176800** (45,178,234..45,191,719) Protein kinase superfamily protein |  |
| **11** | 47.0 | 48.0 | 7.3062E-06 | 1.526E-06 | 0 |  |  |  |
| **11** | 48.0 | 49.0 | 1.5156E-06 | 4.9944E-07 | 0 |  |  |  |
| **11** | 53.0 | 54.0 | 6.8958E-07 | 2.2768E-07 | 11 |  | **Phvul.011G215400** (50,112,467..50,116,453) RING/U-box superfamily protein | Genes underlying QTL (53.5Mbp) associated with hundred seed weight in the Middle American diversity panel (Wilker et al, unpublished). |
|  |  |  |  |  |  |  | **Phvul.011G215500** (50,117,514..50,119,873) organic cation/carnitine transporter 2 |  |
|  |  |  |  |  |  |  | **Phvul.011G215600** (50,122,884..50,125,029) organic cation/carnitine transporter |  |
|  |  |  |  |  |  |  | **Phvul.011G215700** (50,126,054..50,130,876) unknown protein |  |
|  |  |  |  |  |  |  | **Phvul.011G215800** (50,132,400..50,137,832) eukaryotic translation initiation factor SUI1 family protein |  |
|  |  |  |  |  |  |  | **Phvul.011G215900** (50,142,442..50,154,147) dynamin-like 3 (GTPase activity, GTP binding) |  |
|  |  |  |  |  |  |  | **Phvul.011G216000** (50,155,120..50,159,314) Galactose-binding protein |  |
|  |  |  |  |  |  |  | **Phvul.011G216100** (50,160,731..50,171,829) Molybdenum cofactor sulfurase family protein |  |
|  |  |  |  |  |  |  | **Phvul.011G216200** (50,176,173..50,185,138) ATP binding protein, putative n=1 Tax=Ricinus communis RepID=B9SHL9_RICCO |  |
|  |  |  |  |  |  |  | **Phvul.011G216300** (50,188,296..50,190,525) Peroxidase superfamily protein |  |
|  |  |  |  |  |  |  | **Phvul.011G216400** (50,196,967..50,204,051) Signal transduction histidine kinase, hybrid-type, ethylene sensor |  |

*see Schmutz et al, 2014, "Supplementary Table 16 Mesoamerican domestication candidates" for details.

**Table S1 References**

**(Reference numbers correspond to those assigned in main reference list)**

[67] Ayyappan, V, Kalavacharla, V, Thimmapuram, J, Bhide, KP, Sripathi, VR, Smolinski, TG, Manoharan, M, Thurston, Y, Todd, A, Kingham, B. Genome-Wide Profiling of Histone Modifications (H3K9me2 and H4K12ac) and Gene Expression in Rust (*Uromyces appendiculatus*) Inoculated Common Bean (*Phaseolus vulgaris* L.). PLoS One. 2015; 10(7): e0132176. Published online 2015 Jul 13. doi: 10.1371/journal.pone.0132176

[37] Flores, AC, Via, VD, Savy, V, Villagra, UM, Zanetti, ME, Blanco, F. Comparative phylogenetic and expression analysis of small GTPases families in legume and non-legume plants. Plant Signaling & Behaviour. 2018; 13(2): e1432956. doi: 10.1080/15592324.2018.1432956

[114] González, AM, Godoy, L, Santalla, M. Dissection of Resistance Genes to *Pseudomonas syringae* pv. *phaseolicola* in UI3 Common Bean Cultivar. Int. J. Mol. Sci. 2017; 18. doi: 10.3390/ijms18122503

[38] Guillén, G, Díaz-Camino, C, Loyola-Torres, CA, Aparicio-Fabre, R, Hernández-López, A, Díaz-Sánchez, M, Sanchez, F. Detailed analysis of putative genes enco small proteins in legume genomes. Front. Plant Sci. 2013. doi: 10.3389/fpls.2013.00208

[33] Heilig, JA, Beaver, JS, Wright, EM, Song, Q, Kelly, JD. QTL Analysis of Symbiotic Nitrogen Fixation in a Black Bean Population. Crop Sci. 2017; 57. doi: 10.2135/cropsci2016.05.0348

[35] Kamfwa, K, Cichy, KA, Kelly, JD. Identification of quantitative trait loci for symbiotic nitrogen fixation in common bean. Theor. Appl. Genet. 2019; 132. doi: 10.1007/s00122-019-03284-6

[113] Konzen , ER, Recchia, GH, Cassieri, F, Caldas, DGG, Mier y Teran, JCB, Gepts, P, Tsai, SM. DREB genes from common bean (*Phaseolus vulgaris* L.) show broad to specific abiotic stress responses and distinct levels of nucleotide diversity. Hindawi Int. J. Gen. 2019. doi: 10.1155/2019/9520642

[115] Martin, K, Singh, J, Hill, JH, Whitham, SA, Cannon, SB. Dynamic transcriptome profiling of Bean Common Mosaic Virus (BCMV) infection in Common Bean (*Phaseolus vulgaris* L.). BMC Genomics. 2016; 17(613). doi: 10.1186/s12864-016-2976-8

[32] Moghaddam, SM, Mamidi, S, Osorno, JM, Lee, R, Brick, M, Kelly, J, Miklas, P, Urrea, C, Song, Q, Cregan, P, Grimwood, J, Schmutz, J, McClean, PE. Genome-Wide Association Study Identifies Candidate Loci Underlying Agronomic Traits in a Middle American Diversity Panel of Common Bean. Plant Genome. 2016; 9(3): doi: 10.3835/plantgenome2016.02.0012

[111] Mohanta, TK, Kumar, P, Bae, H. Genomics and evolutionary aspect of calcium signaling event in calmodulin and calmodulin-like proteins in plants. BMC Plant Biology. 2017; 17(38). doi: 10.1186/s12870-017-0989-3

[112] Souza, LPd, Scossa, F, Proost, S, Bitocchi, E, Papa, R, Tohge, T, Fernie, AR. Multi-tissue integration of transcriptomic and specialized metabolite profiling provides tools for assessing the common bean (*Phaseolus vulgaris*) metabolome. Plant J. 2019; 97. doi: 10.1111/tpj.14178

[46] Recchia, GH, Konzen, ER, Cassieri, F, Caldas, DGG, Tsai, SM. Arbuscular Mycorrhizal Symbiosis Leads to Differential Regulation of Drought-Responsive Genes in Tissue-Specific Root Cells of Common Bean. Front. Microbiol. 2018. doi: 10.3389/fmicb.2018.01339

[39] Rípodas, C, Castaingts, M, Clúa, J, Blanco, F, Zanetti, ME. Annotation, phylogeny and expression analysis of the nuclear factor Y gene families in common bean (*Phaseolus vulgaris*). Front. Plant Sci. 2015; 5. doi: 10.3389/fpls.2014.00761

[5] Schmutz, J, McClean, PE, Mamidi, S, Wu, GA, Cannon, SB, Grimwood, J, Jenkins, J, Shu, S, Song, Q, Chavarro, C, Torres-Torres, M, Geffroy, V, Moghaddam, SM, Gao, D, Abernathy, B, Barry, K, Blair, M, Brick, MA, Chovatia, M, Gepts, P, Goodstein, DM, Gonzales, M, Hellsten, U, Hyten, DL, Jia, G, Kelly, JD, Kudrna, D, Lee, R, Richard, MMS, Miklas, PN, Osorno, JM, Rodrigues, J, Thareau, V, Urrea, CA, Wang, M, Yu, Y, Zhang, M, Wing, RA, Cregan, PB, Rokhsar, DS, Jackson, SA. A reference genome for common bean and genome-wide analysis of dual domestications. Nature Genetics. 2014; 46(7). doi: 10.1038/ng.3008

[75] Silva, DAd, Tsai, SM, Chiorato, AF, da Silva Andrade, SC, Esteves, JAdF, Recchia, GH, Carbonell, SAM. Analysis of the common bean (*Phaseolus vulgaris* L.) transcriptome regarding efficiency of phosphorus use. PLoS ONE. 2019; 14(1). doi: 10.1371/journal.pone.0210428

**Table S2.** Candidate genes identified in regions of the *P. vulgaris* (2.0) genome where SNPs with significantly high weighted *F*_ST_ values (> 0.5) were found. Candidate genes within 100 Kb of the significant SNP are listed with annotation from JBrowse (<https://legumeinfo.org/genomes/jbrowse/>). (Chr – chromosome)

| **Chr** | **Position (Mbp)** | ***F*_ST_ value** | **Candidate genes annotated** |
| --- | --- | --- | --- |
| 2 | 48,908,956 | 0.541 | **Phvul.002G323708**  Disease resistance protein (TIR-NBS-LRR class) family; IPR025875 (Leucine rich repeat 4)  Position Pv02: 48,873,058..48,884,543  *(reference: Oladzad et al 2019, [40])*  **Phvul.002G323712**  Disease resistance protein (TIR-NBS-LRR class), putative; IPR000157 (Toll/interleukin-1 receptor homology (TIR) domain), IPR000767 (Disease resistance protein), IPR025875 (Leucine rich repeat 4), IPR027417 (P-loop containing nucleoside triphosphate hydrolase); GO:0000166 (nucleotide binding), GO:0005515 (protein binding), GO:0006952 (defense response), GO:0007165 (signal transduction), GO:0017111 (nucleoside-triphosphatase activity), GO:0043531 (ADP binding)  Position Pv02: 48,888,578..48,895,789  *(reference: Oladzad et al 2019, [40])*  **Phvul.002G323900**  Aspartate-semialdehyde dehydrogenase; IPR012080 (Aspartate-semialdehyde dehydrogenase), IPR016040 (NAD(P)-binding domain); GO:0003942 (N-acetyl-gamma-glutamyl-phosphate reductase activity), GO:0004073 (aspartate-semialdehyde dehydrogenase activity), GO:0005737 (cytoplasm), GO:0008652 (cellular amino acid biosynthetic process), GO:0009086 (methionine biosynthetic process), GO:0009088 (threonine biosynthetic process), GO:0009089 (lysine biosynthetic process via diaminopimelate), GO:0009097 (isoleucine biosynthetic process), GO:0046983 (protein dimerization activity), GO:0050661 (NADP binding), GO:0051287 (NAD binding), GO:0055114 (oxidation-reduction process)  Position Pv02: 48,895,240..48,898,436  **Phvul.002G324100**  PLATZ transcription factor family protein; IPR000315 (Zinc finger, B-box), IPR006734 (Protein of unknown function DUF597); GO:0005622 (intracellular), GO:0008270 (zinc ion binding)  Position Pv02: 48,906,246..48,908,726  **Phvul.002G324200**  Mitochondrial import receptor subunit TOM40-1-like [Glycine max]; IPR023614 (Porin domain), IPR027246 (Eukaryotic porin/Tom40); GO:0005741 (mitochondrial outer membrane), GO:0055085 (transmembrane transport)  Position Pv02: 48,913,224..48,918,020  **Phvul.002G324300**  40S ribosomal protein S16-like [Glycine max]; IPR000754 (Ribosomal protein S9), IPR020568 (Ribosomal protein S5 domain 2-type fold); GO:0003735 (structural constituent of ribosome), GO:0005840 (ribosome), GO:0006412 (translation)  Position Pv02: 48,919,930..48,920,791  **Phvul.002G324400**  Clathrin coat assembly protein AP180-like [Glycine max]; IPR008942 (ENTH/VHS), IPR011417 (AP180 N-terminal homology (ANTH) domain); GO:0005543 (phospholipid binding), GO:0005545 (1-phosphatidylinositol binding), GO:0030118 (clathrin coat), GO:0030276 (clathrin binding), GO:0048268 (clathrin coat assembly) Position Pv02: 48,924,528..48,925,847  **Phvul.002G324500**  THO complex subunit 2; IPR021418 (THO complex, subunitTHOC2, C-terminal), IPR021726 (THO complex, subunitTHOC2, N-terminal) Position Pv02: 48,926,349..48,950,645 |
| 2 | 49,075,854 | 0.546 | **Phvul.002G325500**  Disease resistance-responsive (dirigent-like protein) family protein; IPR004265 (Plant disease resistance response protein)  Position Pv02: 49,039,240..49,039,764  **Phvul.002G325600**  Uncharacterized protein LOC100820443 [Glycine max]; IPR006747 (Protein of unknown function DUF599)  Position Pv02: 49,053,148..49,053,874  **Phvul.002G325700**  Uncharacterized protein LOC100820443 [Glycine max]; IPR006747 (Protein of unknown function DUF599)  Position Pv02: 49,055,984..49,058,744  **Phvul.002G325800**  Uncharacterized protein LOC100820443 [Glycine max]; IPR006747 (Protein of unknown function DUF599)  Position Pv02: 49,062,024..49,064,981  **Phvul.002G326000**  Uncharacterized protein LOC100787767 [Glycine max]; IPR004864 (Late embryogenesis abundant protein, LEA-14)  Position Pv02: 49,075,423..49,076,898  **Phvul.002G326100**  2-oxoglutarate (2OG) and Fe(II)-dependent oxygenase superfamily protein; IPR005123 (Oxoglutarate/iron-dependent dioxygenase); GO:0005506 (iron ion binding), GO:0016491 (oxidoreductase activity), GO:0031418 (L-ascorbic acid binding), GO:0055114 (oxidation-reduction process)  Position Pv02: 49,081,592..49,085,315  **Phvul.002G326200**  Pentatricopeptide repeat (PPR) superfamily protein; IPR002885 (Pentatricopeptide repeat), IPR011990 (Tetratricopeptide-like helical); GO:0005515 (protein binding)  Position Pv02: 49,086,617..49,089,190  *(reference: MacQueen et al 2020, [34])*  **Phvul.002G326300**  T-complex protein 1 subunit delta, putative; IPR002423 (Chaperonin Cpn60/TCP-1), IPR027409 (GroEL-like apical domain), IPR027410 (TCP-1-like chaperonin intermediate domain), IPR027413 (GroEL-like equatorial domain); GO:0005524 (ATP binding), GO:0006457 (protein folding), GO:0044267 (cellular protein metabolic process), GO:0051082 (unfolded protein binding)  Position Pv02: 49,090,552..49,092,942  **Phvul.002G326400**  ATP-dependent RNA helicase, putative; IPR001650 (Helicase, C-terminal), IPR007502 (Helicase-associated domain), IPR011709 (Domain of unknown function DUF1605), IPR014001 (Helicase, superfamily 1/2, ATP-binding domain), IPR014720 (Double-stranded RNA-binding domain), IPR027417 (P-loop containing nucleoside triphosphate hydrolase); GO:0003676 (nucleic acid binding), GO:0004386 (helicase activity), GO:0005524 (ATP binding), GO:0008026 (ATP-dependent helicase activity)  Position Pv02: 49,094,816..49,105,934  **Phvul.002G326500**  Glycosyltransferase family 61 protein; IPR007657 (Glycosyltransferase AER61, uncharacterised)  Position Pv02: 49,107,649..49,109,932  **Phvul.002G326600**  1-aminocyclopropane-1-carboxylate oxidase; IPR005123 (Oxoglutarate/iron-dependent dioxygenase), IPR026992 (Non-haem dioxygenase N-terminal domain), IPR027443 (Isopenicillin N synthase-like); GO:0016491 (oxidoreductase activity), GO:0055114 (oxidation-reduction process)  Position Pv02: 49,114,990..49,116,625  *(reference: Nova-Franco et al 2015, [42])* |
| 2 | 49,136,945 | 0.603 | **Phvul.002G326300**  T-complex protein 1 subunit delta, putative; IPR002423 (Chaperonin Cpn60/TCP-1), IPR027409 (GroEL-like apical domain), IPR027410 (TCP-1-like chaperonin intermediate domain), IPR027413 (GroEL-like equatorial domain); GO:0005524 (ATP binding), GO:0006457 (protein folding), GO:0044267 (cellular protein metabolic process), GO:0051082 (unfolded protein binding)  Position Pv02: 49,090,552..49,092,942  **Phvul.002G326400**  ATP-dependent RNA helicase, putative; IPR001650 (Helicase, C-terminal), IPR007502 (Helicase-associated domain), IPR011709 (Domain of unknown function DUF1605), IPR014001 (Helicase, superfamily 1/2, ATP-binding domain), IPR014720 (Double-stranded RNA-binding domain), IPR027417 (P-loop containing nucleoside triphosphate hydrolase); GO:0003676 (nucleic acid binding), GO:0004386 (helicase activity), GO:0005524 (ATP binding), GO:0008026 (ATP-dependent helicase activity)  Position Pv02: 49,094,816..49,105,934  **Phvul.002G326500**  Glycosyltransferase family 61 protein; IPR007657 (Glycosyltransferase AER61, uncharacterised)  Position Pv02: 49,107,649..49,109,932  **Phvul.002G326600**  1-aminocyclopropane-1-carboxylate oxidase; IPR005123 (Oxoglutarate/iron-dependent dioxygenase), IPR026992 (Non-haem dioxygenase N-terminal domain), IPR027443 (Isopenicillin N synthase-like); GO:0016491 (oxidoreductase activity), GO:0055114 (oxidation-reduction process)  Position Pv02: 49,114,990..49,116,625  *(reference: Nova-Franco et al 2015, [42])*  **Phvul.002G326800**  Protein kinase superfamily protein; IPR011009 (Protein kinase-like domain); GO:0004672 (protein kinase activity), GO:0004674 (protein serine/threonine kinase activity), GO:0005524 (ATP binding), GO:0006468 (protein phosphorylation)  Position Pv02: 49,125,308..49,129,702  **Phvul.002G326900**  uncharacterized protein LOC100785837 [Glycine max]; IPR019448 (EEIG1/EHBP1 N-terminal domain)  Position Pv02: 49,131,317..49,134,457  **Phvul.002G327000**  uncharacterized protein LOC100785302 isoform X1 [Glycine max]  Position Pv02: 49,135,836..49,138,843  **Phvul.002G327100**  GTP binding Elongation factor Tu family protein; IPR000795 (Elongation factor, GTP-binding domain), IPR009000 (Translation protein, beta-barrel domain), IPR009001 (Translation elongation factor EF1A/initiation factor IF2gamma, C-terminal), IPR027417 (P-loop containing nucleoside triphosphate hydrolase); GO:0003924 (GTPase activity), GO:0005525 (GTP binding)  Position Pv02: 49,141,959..49,151,857  **Phvul.002G327200**  Glycosyltransferase family 29 (sialyltransferase) family protein; IPR001675 (Glycosyl transferase, family 29); GO:0006486 (protein glycosylation), GO:0008373 (sialyltransferase activity)  Position Pv02: 49,154,815..49,159,069  **Phvul.002G327300**  pre-mRNA-splicing factor SPF27 homolog [Glycine max]; IPR008409 (Pre-mRNA-splicing factor SPF27); GO:0006397 (mRNA processing)  Position Pv02: 49,161,117..49,164,161  **Phvul.002G327400**  peroxin 3; IPR006966 (Peroxin-3); GO:0005779 (integral component of peroxisomal membrane), GO:0007031 (peroxisome organization)  Position Pv02: 49,166,882..49,171,778  **Phvul.002G327500**  guanine nucleotide-binding protein subunit beta-like protein [Glycine max]; IPR015943 (WD40/YVTN repeat-like-containing domain), IPR020472 (G-protein beta WD-40 repeat); GO:0005515 (protein binding)  Position Pv02: 49,176,943..49,178,861  **Phvul.002G327600**  WD repeat-containing protein 5-like [Glycine max]; IPR011047 (Quinonprotein alcohol dehydrogenase-like superfamily), IPR015943 (WD40/YVTN repeat-like-containing domain); GO:0005515 (protein binding)  Position Pv02: 49,180,858..49,185,410 |
| 2 | 49,236,682  49,239,546 | 0.514  0.501 | **Phvul.002G327700**  flowering locus protein T; IPR008914 (Phosphatidylethanolamine-binding protein PEBP)  Position Pv02: 49,193,717..49,195,868  **Phvul.002G327800**  uncharacterized GPI-anchored protein [Glycine max]  Position Pv02: 49,203,167..49,205,710  **Phvul.002G327900**  uncharacterized protein At5g39865-like [Glycine max]; IPR012336 (Thioredoxin-like fold); GO:0009055 (electron carrier activity), GO:0015035 (protein disulfide oxidoreductase activity), GO:0045454 (cell redox homeostasis)  Position Pv02: 49,209,233..49,210,093  **Phvul.002G328000**  pentatricopeptide (PPR) repeat-containing protein; IPR002885 (Pentatricopeptide repeat), IPR011990 (Tetratricopeptide-like helical); GO:0005515 (protein binding)  Position Pv02: 49,211,691..49,214,375  **Phvul.002G328100**  pentatricopeptide (PPR) repeat-containing protein; IPR002885 (Pentatricopeptide repeat), IPR011990 (Tetratricopeptide-like helical); GO:0005515 (protein binding)  Position Pv02: 49,216,072..49,219,019  **Phvul.002G328104**  Eukaryotic aspartyl protease family protein; IPR021109 (Aspartic peptidase domain)  Position Pv02: 49,221,401..49,223,395  **Phvul.002G328300**  Protein kinase superfamily protein; IPR011009 (Protein kinase-like domain); GO:0004672 (protein kinase activity), GO:0004707 (MAP kinase activity), GO:0005524 (ATP binding), GO:0006468 (protein phosphorylation)  Position Pv02: 49,224,547..49,229,755  *(reference: Zuiderveen et al 2016, [36])*  **Phvul.002G328400**  Winged-helix DNA-binding transcription factor family protein, putative isoform 1 n=2 Tax=Theobroma cacao RepID=UPI00042B60CA; IPR011991 (Winged helix-turn-helix DNA-binding domain), IPR020478 (AT hook-like); GO:0000786 (nucleosome), GO:0003677 (DNA binding), GO:0005634 (nucleus), GO:0006334 (nucleosome assembly)  Position Pv02: 49,236,361..49,239,509  **Phvul.002G234300**  SMAD/FHA domain-containing protein; IPR008984 (SMAD/FHA domain); GO:0005515 (protein binding)  Position Pv02: 49,253,244..49,254,133  **Phvul.002G234304**  uncharacterized protein LOC100783352 isoform X1 [Glycine max]; IPR026847 (Vacuolar protein sorting-associated protein 13)  Position Pv02: 49,268,291..49,303,912 |
| 7 | 638,614  651,195 | 0.558  0.558 | **Phvul.007G008200**  ATP-binding ABC transporter; IPR013525 (ABC-2 type transporter), IPR027417 (P-loop containing nucleoside triphosphate hydrolase); GO:0000166 (nucleotide binding), GO:0005524 (ATP binding), GO:0016020 (membrane), GO:0016887 (ATPase activity), GO:0017111 (nucleoside-triphosphatase activity)  Position Pv07: 592,430..594,703  **Phvul.007G008300**  U4/U6 small nuclear ribonucleoprotein Prp3-like isoform X5 [Glycine max]; IPR010541 (Domain of unknown function DUF1115), IPR013881 (Pre-mRNA-splicing factor 3), IPR027104 (U4/U6 small nuclear ribonucleoprotein Prp3); GO:0046540 (U4/U6 x U5 tri-snRNP complex)  Position Pv07: 601,126..604,961  **Phvul.007G008400**  Peroxidase superfamily protein; IPR010255 (Haem peroxidase); GO:0004601 (peroxidase activity), GO:0006979 (response to oxidative stress), GO:0020037 (heme binding), GO:0055114 (oxidation-reduction process)  Position Pv07: 607,208..609,910  **Phvul.007G008500**  chalcone-flavanone isomerase family protein; IPR016087 (Chalcone isomerase); GO:0009813 (flavonoid biosynthetic process), GO:0016872 (intramolecular lyase activity), GO:0045430 (chalcone isomerase activity)  Position Pv07: 612,607..614,403  *(reference: Przysiecka et al 2015, [120])*  **Phvul.007G008600**  chalcone-flavanone isomerase family protein; IPR016087 (Chalcone isomerase); GO:0009813 (flavonoid biosynthetic process), GO:0016872 (intramolecular lyase activity), GO:0045430 (chalcone isomerase activity)  Position Pv07: 616,509..618,799  **Phvul.007G008700**  hypothetical protein  Position Pv07: 623,768..635,464  **Phvul.007G008800**  probable membrane-associated kinase regulator 4-like [Glycine max]  Position Pv07: 637,987..639,284  **Phvul.007G008900**  Ankyrin repeat family protein; IPR020683 (Ankyrin repeat-containing domain), IPR026961 (PGG domain), IPR027001 (Caskin/Ankyrin repeat-containing protein); GO:0005515 (protein binding)  Position Pv07: 643,030..647,444  **Phvul.007G009000**  Catalytic/ protein phosphatase type 2C/ protein serine/threonine phosphatase n=6 Tax=Panicoideae RepID=B6TEB8_MAIZE; IPR001932 (Protein phosphatase 2C (PP2C)-like domain), IPR015655 (Protein phosphatase 2C); GO:0003824 (catalytic activity), GO:0004722 (protein serine/threonine phosphatase activity), GO:0006470 (protein dephosphorylation)  Position Pv07: 649,915..654,480  **Phvul.007G009100**  3-ketoacyl-CoA synthase 19; IPR012392 (Very-long-chain 3-ketoacyl-CoA synthase), IPR016039 (Thiolase-like); GO:0003824 (catalytic activity), GO:0006633 (fatty acid biosynthetic process), GO:0008152 (metabolic process), GO:0008610 (lipid biosynthetic process), GO:0016020 (membrane)  Position Pv07: 657,680..659,317  **Phvul.007G009200**  3-ketoacyl-CoA synthase 19; IPR012392 (Very-long-chain 3-ketoacyl-CoA synthase), IPR016039 (Thiolase-like); GO:0003824 (catalytic activity), GO:0006633 (fatty acid biosynthetic process), GO:0008152 (metabolic process), GO:0008610 (lipid biosynthetic process), GO:0016020 (membrane)  Position Pv07: 661,883..663,334  **Phvul.007G009300**  Pectate lyase family protein; IPR011050 (Pectin lyase fold/virulence factor), IPR018082 (AmbAllergen)  Position Pv07: 673,746..677,076  **Phvul.007G009400**  60S ribosomal L35-like protein; IPR001854 (Ribosomal protein L29); GO:0003735 (structural constituent of ribosome), GO:0005622 (intracellular), GO:0005840 (ribosome), GO:0006412 (translation)  Position Pv07: 678,839..680,686  **Phvul.007G009500**  caffeoylshikimate esterase-like isoform X1 [Glycine max]; IPR000073 (Alpha/beta hydrolase fold-1), IPR022742 (Putative lysophospholipase)  Position Pv07: 686,105..689,556  **Phvul.007G009600**  splicing factor 3B subunit-like protein; IPR004871 (Cleavage/polyadenylation specificity factor, A subunit, C-terminal), IPR011047 (Quinonprotein alcohol dehydrogenase-like superfamily), IPR015943 (WD40/YVTN repeat-like-containing domain); GO:0003676 (nucleic acid binding), GO:0005515 (protein binding), GO:0005634 (nucleus)  Position Pv07: 690,051..695,717 |
| 7 | 4,174,714  4,209,419 | 0.505  0.511 | **Phvul.007G050800**  cysteine-rich receptor-like protein kinase 25-like [Glycine max]; IPR002902 (Gnk2-homologous domain)  Position Pv07: 4,130,561..4,131,931  **Phvul.007G050900**  cysteine-rich receptor-like protein kinase 25-like [Glycine max]; IPR002902 (Gnk2-homologous domain)  Position Pv07: 4,133,493..4,135,225  **Phvul.007G051000**  receptor kinase 2; IPR002902 (Gnk2-homologous domain), IPR011009 (Protein kinase-like domain), IPR013320 (Concanavalin A-like lectin/glucanase, subgroup); GO:0004672 (protein kinase activity), GO:0004674 (protein serine/threonine kinase activity), GO:0005524 (ATP binding), GO:0006468 (protein phosphorylation)  Position Pv07: 4,137,152..4,140,925  **Phvul.007G051100**  cysteine-rich RLK (RECEPTOR-like protein kinase) 25; IPR002902 (Gnk2-homologous domain), IPR011009 (Protein kinase-like domain), IPR013320 (Concanavalin A-like lectin/glucanase, subgroup); GO:0004672 (protein kinase activity), GO:0004674 (protein serine/threonine kinase activity), GO:0005524 (ATP binding), GO:0006468 (protein phosphorylation)  Position Pv07: 4,146,360..4,150,390  **Phvul.007G051200**  cysteine-rich RLK (RECEPTOR-like protein kinase) 25; IPR002902 (Gnk2-homologous domain), IPR011009 (Protein kinase-like domain), IPR013320 (Concanavalin A-like lectin/glucanase, subgroup); GO:0004672 (protein kinase activity), GO:0004674 (protein serine/threonine kinase activity), GO:0005524 (ATP binding), GO:0006468 (protein phosphorylation)  Position Pv07: 4,151,954..4,155,957  **Phvul.007G051300**  cysteine-rich RLK (RECEPTOR-like protein kinase) 25; IPR002902 (Gnk2-homologous domain), IPR011009 (Protein kinase-like domain), IPR013320 (Concanavalin A-like lectin/glucanase, subgroup); GO:0004672 (protein kinase activity), GO:0004674 (protein serine/threonine kinase activity), GO:0005524 (ATP binding), GO:0006468 (protein phosphorylation)  Position Pv07: 4,160,841..4,165,305  **Phvul.007G051401**  putative cysteine-rich receptor-like protein kinase 23-like [Glycine max]; IPR002902 (Gnk2-homologous domain)  Position Pv07: 4,180,350..4,182,754  **Phvul.007G051500**  receptor kinase 2; IPR002902 (Gnk2-homologous domain), IPR011009 (Protein kinase-like domain), IPR013320 (Concanavalin A-like lectin/glucanase, subgroup); GO:0004672 (protein kinase activity), GO:0004674 (protein serine/threonine kinase activity), GO:0005524 (ATP binding), GO:0006468 (protein phosphorylation)  Position Pv07: 4,198,645..4,203,117  **Phvul.007G051600**  GDSL esterase/lipase At5g33370-like [Glycine max]; IPR013831 (SGNH hydrolase-type esterase domain), IPR015939 (Fumarate reductase/succinate dehydrogenase flavoprotein-like, C-terminal); GO:0016491 (oxidoreductase activity), GO:0016787 (hydrolase activity), GO:0055114 (oxidation-reduction process)  Position Pv07: 4,209,700..4,213,437  **Phvul.007G051700**  Late embryogenesis abundant (LEA) hydroxyproline-rich glycoprotein family; IPR004864 (Late embryogenesis abundant protein, LEA-14)  Position Pv07: 4,216,491..4,217,619  **Phvul.007G051800**  transmembrane protein, putative  Position Pv07: 4,234,889..4,238,457  **Phvul.007G051900**  pyruvate, phosphate dikinase regulatory protein, putative; IPR005177 (Bifunctional kinase-pyrophosphorylase); GO:0005524 (ATP binding)  Position Pv07: 4,239,917..4,241,740  **Phvul.007G052000**  gibberellin 2-beta-dioxygenase 8-like [Glycine max]; IPR005123 (Oxoglutarate/iron-dependent dioxygenase), IPR026992 (Non-haem dioxygenase N-terminal domain), IPR027443 (Isopenicillin N synthase-like); GO:0016491 (oxidoreductase activity), GO:0055114 (oxidation-reduction process)  Position Pv07: 4,242,844..4,245,600 |
| 7 | 4,702,892 | 0.597 | **Phvul.007G055800**  seed linoleate 9S-lipoxygenase; IPR000907 (Lipoxygenase), IPR008976 (Lipase/lipooxygenase, PLAT/LH2), IPR027433 (Lipoxygenase, domain 3); GO:0005506 (iron ion binding), GO:0005515 (protein binding), GO:0016165 (linoleate 13S-lipoxygenase activity), GO:0046872 (metal ion binding), GO:0055114 (oxidation-reduction process)  Position Pv07: 4,657,074..4,663,363  **Phvul.007G055900**  photosystem I reaction center subunit II; IPR003685 (Photosystem I PsaD); GO:0009522 (photosystem I), GO:0009538 (photosystem I reaction center), GO:0015979 (photosynthesis)  Position Pv07: 4,666,084..4,667,007  **Phvul.007G056000**  haloacid dehalogenase-like hydrolase; IPR006439 (HAD hydrolase, subfamily IA), IPR010237 (Pyrimidine 5-nucleotidase), IPR023214 (HAD-like domain); GO:0008152 (metabolic process), GO:0016787 (hydrolase activity)  Position Pv07: 4,670,049..4,672,373  **Phvul.007G056100**  spermidine synthase 1; IPR001045 (Spermidine/spermine synthases family); GO:0003824 (catalytic activity)  Position Pv07: 4,712,606..4,715,663  **Phvul.007G056200**  Copper amine oxidase family protein; IPR000269 (Copper amine oxidase); GO:0005507 (copper ion binding), GO:0008131 (primary amine oxidase activity), GO:0009308 (amine metabolic process), GO:0048038 (quinone binding), GO:0055114 (oxidation-reduction process)  Position Pv07: 4,726,727..4,730,374  **Phvul.007G056300**  hypothetical protein  Position Pv07: 4,738,183..4,738,785  **Phvul.007G056400**  Copper amine oxidase family protein; IPR000269 (Copper amine oxidase); GO:0005507 (copper ion binding), GO:0008131 (primary amine oxidase activity), GO:0009308 (amine metabolic process), GO:0048038 (quinone binding), GO:0055114 (oxidation-reduction process)  Position Pv07: 4,742,026..4,744,904 |
| 7 | 38,944,990 | 0.574 | **Phvul.007G267900**  BEL1-like homeodomain protein 1-like isoform X2 [Glycine max]; IPR006563 (POX domain), IPR009057 (Homeodomain-like); GO:0003677 (DNA binding), GO:0003700 (sequence-specific DNA binding transcription factor activity), GO:0043565 (sequence-specific DNA binding)  Position Pv07: 38,901,727..38,906,220  **Phvul.007G268000**  NC domain-containing protein-related; IPR000064 (Endopeptidase, NLPC/P60 domain), IPR007053 (LRAT-like domain)  Position Pv07: 38,922,613..38,924,853  **Phvul.007G268100**  TGF beta-inducible nuclear protein 1 n=21 Tax=Endopterygota RepID=Q1W759_BOMMO; IPR022309 (Ribosomal protein S8e/ribosomal biogenesis NSA2)  Position Pv07: 38,926,720..38,929,382  **Phvul.007G268200**  Protein kinase superfamily protein; IPR001611 (Leucine-rich repeat), IPR003591 (Leucine-rich repeat, typical subtype), IPR011009 (Protein kinase-like domain), IPR013320 (Concanavalin A-like lectin/glucanase, subgroup); GO:0004672 (protein kinase activity), GO:0004674 (protein serine/threonine kinase activity), GO:0005515 (protein binding), GO:0005524 (ATP binding), GO:0006468 (protein phosphorylation)  Position Pv07: 38,944,235..38,948,252  *(reference: Duwadi et al 2018, [43])*  **Phvul.007G268300**  uncharacterized protein LOC100790193 [Glycine max]  Position Pv07: 38,950,910..38,952,376  **Phvul.007G268400**  Late embryogenesis abundant (LEA) hydroxyproline-rich glycoprotein family  Position Pv07: 38,960,563..38,961,721  **Phvul.007G268500**  uncharacterized protein [Glycine max]  Position Pv07: 38970861..38,971,710  **Phvul.007G268600**  Late embryogenesis abundant (LEA) hydroxyproline-rich glycoprotein family; IPR004864 (Late embryogenesis abundant protein, LEA-14)  Position Pv07: 38,981,117..38,982,594  **Phvul.007G268700**  protein YLS9 [Glycine max]; IPR004864 (Late embryogenesis abundant protein, LEA-14)  Position Pv07: 38,985,430..38,986,354  **Phvul.007G268800**  protein YLS9-like [Glycine max]  Position Pv07: 38,986,531..38,987,745 |
| 9 | 5,546,515 | 0.656 | **Phvul.009G022600**  Ribosomal protein S25 family protein; IPR004977 (Ribosomal protein S25)  Position Pv09: 5,527,580..5,529,548  **Phvul.009G022700**  DUF241 domain protein; IPR004320 (Protein of unknown function DUF241, plant)  Position Pv09: 5,531,980..5,533,006  **Phvul.009G022800**  uncharacterized protein LOC100809365 [Glycine max]; IPR004320 (Protein of unknown function DUF241, plant)  Position Pv09: 5,536,083..5,537,006  **Phvul.009G022900**  DUF241 domain protein; IPR004320 (Protein of unknown function DUF241, plant)  Position Pv09: 5,538,931..5,539,818  **Phvul.009G023000**  uncharacterized protein LOC100809365 [Glycine max]; IPR004320 (Protein of unknown function DUF241, plant)  Position Pv09:5,549,891..5,550,781  **Phvul.009G023100**  Glucose-1-phosphate adenylyltransferase family protein; IPR011831 (Glucose-1-phosphate adenylyltransferase); GO:0005978 (glycogen biosynthetic process), GO:0008878 (glucose-1-phosphate adenylyltransferase activity), GO:0009058 (biosynthetic process), GO:0016779 (nucleotidyltransferase activity)  Position Pv09: 5,557,792..5,562,086  **Phvul.009G023200**  SNF1-related protein kinase regulatory subunit gamma 1; IPR000644 (CBS domain); GO:0030554 (adenyl nucleotide binding)  Position Pv09: 5,562,406..5,564,703  **Phvul.009G023300**  Rer1 family protein; IPR004932 (Retrieval of early ER protein Rer1); GO:0016021 (integral component of membrane)  Position Pv09: 5,567,146..5,569,659 |
| 9 | 6,874,190 | 0.618 | **Phvul.009G029800**  trehalose-6-phosphate phosphatase; IPR006379 (HAD-superfamily hydrolase, subfamily IIB), IPR023214 (HAD-like domain); GO:0003824 (catalytic activity), GO:0005992 (trehalose biosynthetic process), GO:0008152 (metabolic process)  Position Pv09: 6,838,960..6,841,520  **Phvul.009G029900**  unknown protein; LOCATED IN: chloroplast  Position Pv09: 6,878,373..6,880,001  **Phvul.009G030000**  TPR repeat-containing thioredoxin TTL1-like [Glycine max]; IPR011990 (Tetratricopeptide-like helical), IPR012336 (Thioredoxin-like fold); GO:0005515 (protein binding), GO:0045454 (cell redox homeostasis)  Position Pv09: 6,896,081..6,899,881 |
| 9 | 7,701,485 | 0.546 | **Phvul.009G034800**  Target SNARE coiled-coil domain protein; IPR000727 (Target SNARE coiled-coil domain); GO:0005515 (protein binding)  Position Pv09: 7,655,743..7,658,686  **Phvul.009G034900**  ubiquitin-protein ligase 1; IPR000569 (HECT), IPR003903 (Ubiquitin interacting motif), IPR009060 (UBA-like), IPR010309 (E3 ubiquitin ligase, domain of unknown function DUF908), IPR016024 (Armadillo-type fold), IPR025527 (Domain of unknown function DUF4414); GO:0004842 (ubiquitin-protein ligase activity), GO:0005488 (binding), GO:0005515 (protein binding)  Position Pv09: 7,665,586..7,683,468  **Phvul.009G035000**  CRT (chloroquine-resistance transporter)-like transporter 2  Position Pv09: 7,690,678..7,698,131  **Phvul.009G035100**  neuroguidin-like isoform X3 [Glycine max]; IPR007146 (Sas10/Utp3/C1D)  Position Pv09: 7,703,487..7,706,835  **Phvul.009G035200**  Golgi-body localisation protein domain ; RNA pol II promoter Fmp27 protein domain; IPR019441 (FMP27, GFWDK domain), IPR019443 (FMP27, C-terminal)  Position Pv09: 7,722,501..7,749,619 |
| 9 | 7,807,599  7,872,442 | 0.677  0.589 | **Phvul.009G035400**  GATA transcription factor 15; IPR010399 (Tify), IPR010402 (CCT domain), IPR013088 (Zinc finger, NHR/GATA-type); GO:0003700 (sequence-specific DNA binding transcription factor activity), GO:0005515 (protein binding), GO:0008270 (zinc ion binding), GO:0043565 (sequence-specific DNA binding)  Position Pv09: 7,764,191..7,768,361  **Phvul.009G035500**  BEACH domain-containing protein lvsC-like isoform X4 [Glycine max]; IPR000409 (BEACH domain), IPR008985 (Concanavalin A-like lectin/glucanases superfamily), IPR013320 (Concanavalin A-like lectin/glucanase, subgroup), IPR015943 (WD40/YVTN repeat-like-containing domain), IPR016024 (Armadillo-type fold), IPR023362 (PH-BEACH domain); GO:0005488 (binding), GO:0005515 (protein binding)  Position Pv09: 7,785,251..7,852,293  **Phvul.009G035700**  Unknown protein  Position Pv09: 7,862,516..7,863,493  **Phvul.009G035800**  Protein kinase superfamily protein; IPR011009 (Protein kinase-like domain), IPR028324 (Serine/threonine-protein kinase CTR1/EDR1); GO:0004672 (protein kinase activity), GO:0004674 (protein serine/threonine kinase activity), GO:0005524 (ATP binding), GO:0006468 (protein phosphorylation)  Position Pv09: 7,865,582..7,873,144  **Phvul.009G035900**  Tetratricopeptide repeat (TPR)-like superfamily protein; IPR011990 (Tetratricopeptide-like helical); GO:0005515 (protein binding)  Position Pv09: 7,877,920..7,879,060  **Phvul.009G036000**  RAB geranylgeranyl transferase alpha subunit 1; IPR001611 (Leucine-rich repeat), IPR002088 (Protein prenyltransferase, alpha subunit), IPR025875 (Leucine rich repeat 4); GO:0005515 (protein binding), GO:0008318 (protein prenyltransferase activity), GO:0018342 (protein prenylation)  Position Pv09: 7,881,919..7,886,744  **Phvul.009G036100**  double-stranded-RNA-binding protein 4; IPR011907 (Ribonuclease III); GO:0003723 (RNA binding), GO:0004525 (ribonuclease III activity), GO:0016075 (rRNA catabolic process)  Position Pv09: 7,888,094..7,891,100  **Phvul.009G036200**  NADPH-cytochrome P450 family 2 reductase; IPR001094 (Flavodoxin), IPR023173 (NADPH-cytochrome p450 reductase, FAD-binding, alpha-helical domain-3); GO:0003958 (NADPH-hemoprotein reductase activity), GO:0005506 (iron ion binding), GO:0010181 (FMN binding), GO:0016491 (oxidoreductase activity), GO:0055114 (oxidation-reduction process)  Position Pv09: 7,905,486..7,913,740  **Phvul.009G036300**  myb-like DNA-binding domain protein; IPR009057 (Homeodomain-like); GO:0003677 (DNA binding), GO:0003682 (chromatin binding)  Position Pv09: 7,916,029..7,924,420 |
| 9 | 13,540,452  13,555,680 | 0.536  0.536 | **Phvul.009G081700**  Cytochrome P450 superfamily protein; IPR001128 (Cytochrome P450); GO:0005506 (iron ion binding), GO:0020037 (heme binding), GO:0055114 (oxidation-reduction process)  Position Pv09: 13,507,606..13,510,042  **Phvul.009G081800**  cytokinin oxidase/dehydrogenase 1; IPR016164 (FAD-linked oxidase-like, C-terminal), IPR016166 (FAD-binding, type 2), IPR016170 (Vanillyl-alcohol oxidase/Cytokinin dehydrogenase C-terminal domain); GO:0003824 (catalytic activity), GO:0008762 (UDP-N-acetylmuramate dehydrogenase activity), GO:0009690 (cytokinin metabolic process), GO:0016491 (oxidoreductase activity), GO:0019139 (cytokinin dehydrogenase activity), GO:0050660 (flavin adenine dinucleotide binding), GO:0055114 (oxidation-reduction process)  Position Pv09: 13,517,847..13,521,865  *(reference: Recchia et al 2018, [45])*  **Phvul.009G081900**  F-box/LRR-repeat protein 15-like [Glycine max]; IPR001810 (F-box domain), IPR006553 (Leucine-rich repeat, cysteine-containing subtype); GO:0005515 (protein binding)  Position Pv09: 13,539,823..13,545,847  **Phvul.009G082000**  uncharacterized protein LOC100787776 [Glycine max]  Position Pv09: 13,547,643..13,550,505  **Phvul.009G082100**  ferredoxin-related; IPR014044 (CAP domain)  Position Pv09: 13,553,843..13,556,650  **Phvul.009G082200**  uncharacterized protein LOC102659395 isoform X3 [Glycine max]  Position Pv09: 13,557,884..13,558,507  **Phvul.009G082300**  YGGT family protein; IPR003425 (Uncharacterised protein family Ycf19); GO:0016020 (membrane)  Position Pv09: 13,560,871..13,562,756  **Phvul.009G082400**  P-ATPase family transporter: copper ion; heavy metal transporting P-type ATPase-like protein n=1 Tax=Ostreococcus lucimarinus (strain CCE9901) RepID=A4S4X5_OSTLU; IPR001757 (Cation-transporting P-type ATPase), IPR023214 (HAD-like domain); GO:0000166 (nucleotide binding), GO:0006812 (cation transport), GO:0016021 (integral component of membrane), GO:0019829 (cation-transporting ATPase activity), GO:0030001 (metal ion transport), GO:0046872 (metal ion binding)  Position Pv09: 13,563,555..13,573,390  **Phvul.009G082466**  receptor-like protein kinase 2; IPR001611 (Leucine-rich repeat), IPR003591 (Leucine-rich repeat, typical subtype), IPR013210 (Leucine-rich repeat-containing N-terminal, type 2); GO:0005515 (protein binding)  Position Pv09: 13,588,429..13,593,654  **Phvul.009G082532**  ATP binding/protein serine/threonine kinase [Glycine max]; IPR011009 (Protein kinase-like domain); GO:0004672 (protein kinase activity), GO:0004674 (protein serine/threonine kinase activity), GO:0005524 (ATP binding), GO:0006468 (protein phosphorylation)  Position Pv09: 13,593,710..13,595,345  **Phvul.009G082600**  myosin, putative; IPR000048 (IQ motif, EF-hand binding site), IPR001609 (Myosin head, motor domain), IPR002710 (Dilute), IPR004009 (Myosin, N-terminal, SH3-like), IPR027417 (P-loop containing nucleoside triphosphate hydrolase); GO:0003774 (motor activity), GO:0005515 (protein binding), GO:0005524 (ATP binding), GO:0016459 (myosin complex)  Position Pv09: 13,597,488..13,621,793 |
| 9 | 13,667,257 | 0.556 | **Phvul.009G082800**  endoglucanase 16-like [Glycine max]; IPR001701 (Glycoside hydrolase, family 9), IPR008928 (Six-hairpin glycosidase-like); GO:0003824 (catalytic activity), GO:0005975 (carbohydrate metabolic process)  Position Pv09: 13,623,739..13,626,641  **Phvul.009G082900**  guanine nucleotide-binding protein alpha-1 subunit isoform X3 [Glycine max]; IPR001019 (Guanine nucleotide binding protein (G-protein), alpha subunit), IPR027417 (P-loop containing nucleoside triphosphate hydrolase); GO:0003924 (GTPase activity), GO:0004871 (signal transducer activity), GO:0005525 (GTP binding), GO:0006184 (GTP catabolic process), GO:0007165 (signal transduction), GO:0007186 (G-protein coupled receptor signaling pathway), GO:0019001 (guanyl nucleotide binding), GO:0031683 (G-protein beta/gamma-subunit complex binding)  Position Pv09: 13,627,456..13,632,983  **Phvul.009G083000**  Unknown protein  Position Pv09: 13,632,025..13,632,978  **Phvul.009G083100**  alpha/beta-Hydrolases superfamily protein; IPR000073 (Alpha/beta hydrolase fold-1)  Position Pv09: 13,649,605..13,651,144  **Phvul.009G083200**  alpha/beta-Hydrolases superfamily protein; IPR000073 (Alpha/beta hydrolase fold-1)  Position Pv09: 13,652,896..13,655,387  **Phvul.009G083300**  Protein kinase superfamily protein; IPR011009 (Protein kinase-like domain), IPR013320 (Concanavalin A-like lectin/glucanase, subgroup); GO:0004672 (protein kinase activity), GO:0004674 (protein serine/threonine kinase activity), GO:0005524 (ATP binding), GO:0006468 (protein phosphorylation)  Position Pv09: 13,660,303..13,662,564  **Phvul.009G083400**  copper transporter 5; IPR007274 (Ctr copper transporter); GO:0005375 (copper ion transmembrane transporter activity), GO:0016021 (integral component of membrane), GO:0035434 (copper ion transmembrane transport)  Position Pv09: 13,663,149..13,664,162  **Phvul.009G083500**  uncharacterized protein LOC100813662 [Glycine max]; IPR012876 (Protein of unknown function DUF1677, plant)  Position Pv09: 13,666,413..13,667,873  **Phvul.009G083600**  Smr (small MutS-related) domain protein; IPR002625 (Smr protein/MutS2 C-terminal), IPR013899 (Domain of unknown function DUF1771)  Position Pv09: 13,677,996..13,684,415  **Phvul.009G083700**  Pentatricopeptide repeat (PPR) superfamily protein; IPR002885 (Pentatricopeptide repeat), IPR011990 (Tetratricopeptide-like helical); GO:0005515 (protein binding)  Position Pv09: 13,686,702..13,690,344  **Phvul.009G083800**  DEAD-box ATP-dependent RNA helicase 21-like isoform 1 [Glycine max]; IPR000999 (Ribonuclease III domain), IPR001650 (Helicase, C-terminal), IPR003100 (Argonaute/Dicer protein, PAZ domain), IPR005034 (Dicer dimerisation domain), IPR014001 (Helicase, superfamily 1/2, ATP-binding domain), IPR027417 (P-loop containing nucleoside triphosphate hydrolase); GO:0003676 (nucleic acid binding), GO:0003723 (RNA binding), GO:0004386 (helicase activity), GO:0004525 (ribonuclease III activity), GO:0005515 (protein binding), GO:0005524 (ATP binding), GO:0006396 (RNA processing), GO:0008026 (ATP-dependent helicase activity)  Position Pv09: 13,693,775..13,712,211 |
| 9 | 30,555,204 | 0.543 | **Phvul.009G200800**  uncharacterized protein LOC100775409 [Glycine max]  Position Pv09: 30,528,511..30,529,161  **Phvul.009G200900**  protein CHUP1, chloroplastic-like [Glycine max]  Position Pv09: 30,538,426..30,540,922  **Phvul.009G201000**  Adenine nucleotide alpha hydrolases-like superfamily protein; IPR014729 (Rossmann-like alpha/beta/alpha sandwich fold); GO:0006950 (response to stress)  Position Pv09: 30,543,139..30,545,216  **Phvul.009G201100**  Eukaryotic aspartyl protease family protein; IPR001461 (Aspartic peptidase), IPR021109 (Aspartic peptidase domain); GO:0004190 (aspartic-type endopeptidase activity), GO:0006508 (proteolysis)  Position Pv09: 30,558,554..30,559,720  **Phvul.009G201200**  DEAD-box ATP-dependent RNA helicase-like protein; IPR001650 (Helicase, C-terminal), IPR014001 (Helicase, superfamily 1/2, ATP-binding domain), IPR014014 (RNA helicase, DEAD-box type, Q motif), IPR025313 (Domain of unknown function DUF4217), IPR027417 (P-loop containing nucleoside triphosphate hydrolase); GO:0003676 (nucleic acid binding), GO:0004386 (helicase activity), GO:0005524 (ATP binding), GO:0008026 (ATP-dependent helicase activity)  Position Pv09: 30,560,152..30,566,190  **Phvul.009G201300**  ATP binding microtubule motor family protein n=1 Tax=Theobroma cacao RepID=UPI00042B89EE; IPR001752 (Kinesin, motor domain), IPR027417 (P-loop containing nucleoside triphosphate hydrolase), IPR027640 (Kinesin-like protein); GO:0003777 (microtubule motor activity), GO:0005524 (ATP binding), GO:0005871 (kinesin complex), GO:0007018 (microtubule-based movement), GO:0008017 (microtubule binding)  Position Pv09: 30,591,988..30,596,555 |
| 11 | 52,372,623  52,413,349  52,419,673  52,445,931  52,474,138  52,487,507 | 0.553  0.506  0.553  0.553  0.553  0.553 | **Phvul.011G207000**  Pentatricopeptide repeat (PPR-like) superfamily protein; IPR002885 (Pentatricopeptide repeat), IPR011990 (Tetratricopeptide-like helical); GO:0005515 (protein binding)  Position Pv11: 52,348,900..52,357,148  **Phvul.011G206900**  DNA-directed RNA polymerase; IPR006592 (RNA polymerase, N-terminal), IPR007066 (RNA polymerase Rpb1, domain 3), IPR007080 (RNA polymerase Rpb1, domain 1), IPR007081 (RNA polymerase Rpb1, domain 5), IPR015801 (Copper amine oxidase, N2/N3-terminal), IPR021602 (Protein of unknown function DUF3223); GO:0003677 (DNA binding), GO:0003899 (DNA-directed RNA polymerase activity), GO:0005507 (copper ion binding), GO:0009308 (amine metabolic process), GO:0048038 (quinone binding)  Position Pv11: 52,366,172..52,383,595  **Phvul.011G206800**  beta-galactosidase-like [Glycine max]; IPR006101 (Glycoside hydrolase, family 2), IPR008979 (Galactose-binding domain-like), IPR011013 (Galactose mutarotase-like domain), IPR013812 (Glycoside hydrolase, family 2/20, immunoglobulin-like beta-sandwich domain), IPR017853 (Glycoside hydrolase, superfamily), IPR023230 (Glycoside hydrolase, family 2, conserved site), IPR023232 (Glycoside hydrolase, family 2, active site); GO:0003824 (catalytic activity), GO:0004565 (beta-galactosidase activity), GO:0005975 (carbohydrate metabolic process), GO:0009341 (beta-galactosidase complex), GO:0030246 (carbohydrate binding)  Position Pv11: 52,386,468..52,395,605  **Phvul.011G206700**  PREFOLDIN 1; IPR009053 (Prefoldin); GO:0006457 (protein folding), GO:0016272 (prefoldin complex), GO:0051082 (unfolded protein binding)  Position Pv11: 52,404,482..52,407,154  **Phvul.011G206600**  uncharacterized protein LOC100777329 isoform X1 [Glycine max]  Position Pv11: 52,408,489..52,410,925  **Phvul.011G206500**  Amidohydrolase family; IPR011059 (Metal-dependent hydrolase, composite domain), IPR013108 (Amidohydrolase 3)  Position Pv11: 52,411,332..52,421,199  **Phvul.011G206400**  protein LATERAL ROOT PRIMORDIUM 1-like isoform X1 [Glycine max]; IPR007818 (Protein of unknown function DUF702)  Position Pv11: 52,434,964..52,440,240  **Phvul.011G206300**  Phosphatidylinositol-4-phosphate 5-kinase family protein; IPR023610 (Phosphatidylinositol-4-phosphate 5-kinase), IPR027483 (Phosphatidylinositol-4-phosphate 5-kinase, C-terminal), IPR027484 (Phosphatidylinositol-4-phosphate 5-kinase, N-terminal domain); GO:0005524 (ATP binding), GO:0016307 (phosphatidylinositol phosphate kinase activity), GO:0016308 (1-phosphatidylinositol-4-phosphate 5-kinase activity), GO:0046488 (phosphatidylinositol metabolic process)  Position Pv11: 52,442,526..52,447,566  **Phvul.011G206200**  unknown protein; FUNCTIONS IN: molecular_function unknown; INVOLVED IN: biological_process unknown; LOCATED IN: endomembrane system  Position Pv11: 52,485,285..52,489,990  **Phvul.011G206100**  Eukaryotic aspartyl protease family protein; IPR001461 (Aspartic peptidase), IPR021109 (Aspartic peptidase domain); GO:0004190 (aspartic-type endopeptidase activity), GO:0006508 (proteolysis)  Position Pv11: 52,498,725..52,500,499 |

**Table S2 References**

**(Reference numbers correspond to those assigned in main reference list)**

[44] Duwadi, K, Austin, RS, Mainali, HR, Bett, K, Marsolais, F, Dhaubhadel, S. Slow darkening of pinto bean seed coat is associated with significant metabolite and transcript differences related to proanthocyanidin biosynthesis. BMC Genomics. 2018; 19: 260. Published online 2018 Apr 16. doi: 10.1186/s12864-018-4550-z

[116] MacQueen, AH, White, JW, Lee, R, Osorno, JM, Schmutz, J, Miklas, PN, Myers, J, McClean, PE, Juenger, TE. Genetic Associations in Four Decades of Multienvironment Trials Reveal Agronomic Trait Evolution in Common Bean. Genetics. 2020 May; 215(1): 267–284. Published online 2020 Mar 23. doi: 10.1534/genetics.120.303038

[42] Nova-Franco, B, Íñiguez, LP, Valdés-López, O, Alvarado-Affantranger, X, Leija, A, Fuentes, SI, Ramírez, M, Paul, S, Reyes, JL, Girard, L, Hernández, G. The Micro-RNA172c-APETALA2-1 Node as a Key Regulator of the Common Bean-Rhizobium etli Nitrogen Fixation Symbiosis. Plant Physiol. 2015 May; 168(1): 273–291. Published online 2015 Mar 4. doi: 10.1104/pp.114.255547

[40] Oladzad, A, Zitnick-Anderson, K, Jain, S, Simons, K, Osorno, JM, McClean, PE, Pasche, JS. Genotypes and Genomic Regions Associated with *Rhizoctonia solani* Resistance in Common Bean. Front Plant Sci. 2019; 10: 956. Published online 2019 Jul 24. doi: 10.3389/fpls.2019.00956

[117] Przysiecka, Ł, Książkiewicz, M, Wolko, B, Naganowska, B. Structure, expression profile and phylogenetic inference of chalcone isomerase-like genes from the narrow-leafed lupin (*Lupinus angustifolius* L.) genome. Front Plant Sci. 2015; 6: 268. Published online 2015 Apr 21. doi: 10.3389/fpls.2015.00268

[46] Recchia, GH, Konzen, ER, Cassieri, F, Caldas, DGG, Tsai, SM. Arbuscular Mycorrhizal Symbiosis Leads to Differential Regulation of Drought-Responsive Genes in Tissue-Specific Root Cells of Common Bean. Front Microbiol. 2018; 9: 1339. Published online 2018 Jun 21. doi: 10.3389/fmicb.2018.01339

[43] Zuiderveen, GH, Padder, BA, Kamfwa, K, Song, Q, Kelly, JD. Genome-Wide Association Study of Anthracnose Resistance in Andean Beans (*Phaseolus vulgaris*). PLoS One. 2016; 11(6): e0156391. Published online 2016 Jun 6. doi: 10.1371/journal.pone.0156391

**Table S3**. F-test of fixed and Pearson’s χ^2^ test of random effects in the combined GLIMMIX mixed model analysis of the Honduran panel genotypes tested in multiple field locations in Ontario, Canada and Yorito, Honduras, 2014-2015.

|  | **N derived from the atmosphere** | | **Carbon discrimination (Δ)** | | **Flowering** | | **Yield** | | **Hundred seed weight** | |
| --- | --- | --- | --- | --- | --- | --- | --- | --- | --- | --- |
|  | **(%)** | | **(‰)** | | **(days)** | | **(kg ha^-1^)** | | **(g)** | |
| **Fixed effect†** | **F-test** | **P-value** | **F-test** | **P-value** | **F-test** | **P-value** | **F-test** | **P-value** | **F-test** | **P-value** |
| **Genotype (G)** | 3.09 | <.0001 | 4.18 | <.0001 | 11.85 | <.0001 | 1.99 | 0.0008 | 10.37 | <.0001 |
| **Environment (E)** | 49.31 | 0.0051 | 54.87 | 0.0043 | 349.67 | 0.0003 | 180.93 | 0.0007 | 13.74 | 0.0657 |
| **G x E** | 3.79 | <.0001 | 2.34 | <.0001 | 3.52 | <.0001 | 2.18 | <.0001 | 2.20 | 0.0010 |
|  |  |  |  |  |  |  |  |  |  |  |
| **Random effects‡** | **χ^2^** | **Pr> χ^2^** | **χ^2^** | **Pr> χ^2^** | **χ^2^** | **Pr> χ^2^** | **χ^2^** | **Pr> χ^2^** | **χ^2^** | **Pr> χ^2^** |
| **BLOC** | 0.41 | 0.5207 | 6.26 | 0.0123 | 6.38 | 0.0115 | 0.77 | 0.3802 | 0.62 | 0.4321 |
| **IBLK(BLOC)** | 12.86 | 0.0003 | 0.87 | 0.3506 | 1.13 | 0.2880 | 7.30 | 0.0069 | 0.52 | 0.4692 |
| **Residual** | . | . | . | . | . | . | . | . | . | . |

DTF, and HSW were measured at Elora 2014 and 2015 only.

† Genotype (G), Environment (E), and G x E interaction

‡ block (BLOC), and incomplete block (IBLK)

**Table S4**. F-test of fixed effect of genotype overall and by breeding history category, and the Pearson’s χ^2^ test of random effects in the GLIMMIX analysis of 63 genotypes tested at Elora, Ontario, Canada, 2014.

|  | **N derived from the atmosphere** | | **Carbon discrimination (Δ)** | | **Flowering** | | **Maturity** | | **Yield** | | **Hundred seed weight** | |
| --- | --- | --- | --- | --- | --- | --- | --- | --- | --- | --- | --- | --- |
|  | **(%)** | | **(‰)** | | **(days)** | | **(days)** | | **(kg ha^-1^)** | | **(g)** | |
| **Fixed effect†** | **F-test** | **P-value** | **F-test** | **P-value** | **F-test** | **P-value** | **F-test** | **P-value** | **F-test** | **P-value** | **F-test** | **P-value** |
| **Genotype** | 2.52 | 0.0048 | 1.8 | 0.0496 | 5.89 | <.0001 | 8.08 | <.0001 | 10.61 | <.0001 | 5.44 | <.0001 |
|  |  |  |  |  |  |  |  |  |  |  |  |  |
| **Random effects‡** | **χ^2^** | **Pr> χ^2^** | **χ^2^** | **Pr> χ^2^** | **χ^2^** | **Pr> χ^2^** | **χ^2^** | **Pr> χ^2^** | **χ^2^** | **Pr> χ^2^** | **χ^2^** | **Pr> χ^2^** |
| **BLOC** | 0.0 | 1.0 | 0.22 | 0.3192 | 2.4 | 0.1211 | 0.0 | 1.0 | 0.0 | 1.0 | 0.0 | 1.0 |
| **IBLK(BLOC)** | 2.73 | 0.0983 | 0.41 | 0.2620 | 0.0 | 1.0 | 0.0 | 0.9758 | 5.79 | 0.0161 | 0.06 | 0.7990 |
| **Residual** | . | . | . | . | . | . | . | . | . | . | . | . |

† Genotype effects overall and by breeding history subcategory

‡ block (BLOC), and incomplete block (IBLK)

**Table S5**. F-test of fixed effect of genotype overall and by breeding history category, and the Pearson’s χ^2^ test of random effects in the GLIMMIX analysis of 63 genotypes tested at Elora, Ontario, Canada, 2015.

|  | **N derived from the atmosphere** | | **Carbon discrimination (Δ)** | | **Flowering** | | **Maturity** | | **Yield** | | **Hundred seed weight** | |
| --- | --- | --- | --- | --- | --- | --- | --- | --- | --- | --- | --- | --- |
|  | **(%)** | | **(‰)** | | **(days)** | | **(days)** | | **(kg ha^-1^)** | | **(g)** | |
| **Fixed effect†** | **F-test** | **P-value** | **F-test** | **P-value** | **F-test** | **P-value** | **F-test** | **P-value** | **F-test** | **P-value** | **F-test** | **P-value** |
| **Genotype** | 1.61 | 0.0449 | 2.38 | 0.0012 | 3.99 | <.0001 | 3.7 | <.0001 | 3.69 | <.0001 | 7.34 | <.0001 |
|  |  |  |  |  |  |  |  |  |  |  |  |  |
| **Random effects‡** | **χ^2^** | **Pr> χ^2^** | **χ^2^** | **Pr> χ^2^** | **χ^2^** | **Pr> χ^2^** | **χ^2^** | **Pr> χ^2^** | **χ^2^** | **Pr> χ^2^** | **χ^2^** | **Pr> χ^2^** |
| **BLOC** | 0.0 | 1.0 | 3.83 | 0.0251 | 0.0 | 1.0 | 0.0 | 1.0 | 0.0 | 1.0 | 0.53 | 0.4656 |
| **IBLK(BLOC)** | 5.76 | 0.0164 | 0.83 | 0.1811 | 0.0 | 1.0 | 8.09 | 0.0045 | 9.99 | 0.0016 | 0.0 | 1.0 |
| **Residual** | . | . | . | . | . | . | . | . | . | . | . | . |

† Genotype effects overall and by breeding history subcategory

‡ block (BLOC), and incomplete block (IBLK)

**Table S6**. F-test of fixed effect of genotype overall and by breeding history category, and the Pearson’s χ^2^ test of random effects in the GLIMMIX analysis of 63 genotypes tested at Yorito, Honduras, 2014-2015.

|  | **N derived from the atmosphere** | | **Carbon discrimination (Δ)** | | **Yield** | | **Height** | |
| --- | --- | --- | --- | --- | --- | --- | --- | --- |
|  | **(%)** | | **(‰)** | | **(kg ha^-1^)** | | **(cm)** | |
| **Fixed effect†** | **F-test** | **P-value** | **F-test** | **P-value** | **F-test** | **P-value** | **F-test** | **P-value** |
| **Genotype** | 18.82 | <.0001 | 7.19 | <.0001 | 0.94 | 0.5936 | 5.42 | <.0001 |
|  |  |  |  |  |  |  |  |  |
| **Random effects‡** | **χ^2^** | **Pr> χ^2^** | **χ^2^** | **Pr> χ^2^** | **χ^2^** | **Pr> χ^2^** | **χ^2^** | **Pr> χ^2^** |
| **BLOC** | 1.19 | 0.1375 | 1.12 | 0.2890 | 0.0 | 0.4919 | 0.0 | 1.0 |
| **IBLK(BLOC)** | 0.14 | 0.3531 | 0.0 | 1.0 | 0.05 | 0.4117 | 2.85 | 0.0911 |
| **Residual** | 186.47 | <.0001 | . | . | . | . | . | . |

† Genotype effects overall and by breeding history subcategory

‡ block (BLOC), and incomplete block (IBLK)

**Table S7**. F-test of fixed effects and Pearson’s χ^2^ test of random effects in the GLIMMIX mixed-model analysis of leaf chlorophyll content (SPAD) at early vegetative (SPAD1) and reproductive (SPAD2) stages in the Honduran panel overall (Combined) and at Elora in 2014 and 2015.

|  | **Combined** | | | | **Elora 2014** | | | | **Elora 2015** | | | |
| --- | --- | --- | --- | --- | --- | --- | --- | --- | --- | --- | --- | --- |
|  | **SPAD1** | | **SPAD2** | | **SPAD1** | | **SPAD2** | | **SPAD1** | | **SPAD2** | |
| **Fixed effect†** | **F-test** | **P-value** | **F-test** | **P-value** | **F-test** | **P-value** | **F-test** | **P-value** | **F-test** | **P-value** | **F-test** | **P-value** |
| **Genotype (G)** | 6.19 | <.0001 | 6.44 | <.0001 | 2.59 | <.0001 | 3.66 | <.0001 | 10.22 | <.0001 | 8.39 | <.0001 |
| **Environment (E)** | 32.21 | <.0001 | 0.98 | 0.3237 | - | - | - | - | - | - | - | - |
| **G x E** | 3.11 | <.0001 | 4.59 | <.0001 | - | - | - | - | - | - | - | - |
|  |  |  |  |  |  |  |  |  |  |  |  |  |
| **Random effects‡** | **χ^2^** | **Pr> χ^2^** | **χ^2^** | **Pr> χ^2^** | **χ^2^** | **Pr> χ^2^** | **χ^2^** | **Pr> χ^2^** | **χ^2^** | **Pr> χ^2^** | **χ^2^** | **Pr> χ^2^** |
| **BLOC** | . | . | 3.72 | 0.0539 | 1.60 | 0.2055 | 0.21 | 0.6461 | 0.95 | 0.3306 | 1.82 | 0.1767 |
| **IBLK(BLOC)** | . | . | 3.70 | 0.0543 | 2.80 | 0.0941 | 0.0 | 0.9619 | 0.95 | 0.3306 | 1.82 | 0.1767 |
| **Residual** | 3.76 | 0.0526 | . | . | . | . | . | . | . | . | . | . |

SPAD1 and SPAD2 were measured at Elora 2014 and 2015 only.

† Genotype (G), Environment (E), and G x E interaction

‡ block (BLOC), and incomplete block (IBLK)

**Table S8**. Phenotypic (r_p_) correlations among %Ndfa and other traits estimated in the Honduran panel grown in 3 locations in 2014-2015. Values shown for traits within environments are significantly correlated at the 5% level. The number of genotypes analyzed in each correlation is presented in brackets for each significant correlation.

| **Trait**  **Location** | **%Ndfa**  **E14** |  |  |  |  |  |  |  |  |  |  |  |  |  |
| --- | --- | --- | --- | --- | --- | --- | --- | --- | --- | --- | --- | --- | --- | --- |
| **%Ndfa**  **E15** |  | **%Ndfa**  **E15** |  |  |  |  |  |  |  |  |  |  |  |  |
| **%Ndfa**  **YOR** |  |  | **%Ndfa**  **YOR** |  |  |  |  |  |  |  |  |  |  |  |
| **SPAD2**  **E14** |  |  |  | **SPAD2**  **E14** |  |  |  |  |  |  |  |  |  |  |
| **SPAD2**  **E15** |  |  |  |  | **SPAD2**  **E15** |  |  |  |  |  |  |  |  |  |
| **DTF**  **E14** | -0.30704 (49) |  |  |  |  | **DTF**  **E14** |  |  |  |  |  |  |  |  |
| **DTF**  **E15** |  |  |  |  |  |  | **DTF**  **E15** |  |  |  |  |  |  |  |
| **HSW**  **E14** |  |  |  | 0.36357  (49) |  |  |  | **HSW**  **E14** |  |  |  |  |  |  |
| **HSW**  **E15** |  |  |  |  | 0.43931  (62) |  |  |  | **HSW**  **E15** |  |  |  |  |  |
| **ΔC**  **E14** | 0.45399  (48) |  |  |  |  |  |  |  |  | **ΔC**  **E14** |  |  |  |  |
| **ΔC**  **E15** |  |  |  |  |  |  | -0.36797  (57) |  |  |  | **ΔC**  **E15** |  |  |  |
| **ΔC**  **YOR** |  |  |  |  |  |  |  |  |  |  |  | **ΔC**  **YOR** |  |  |
| **Yield**  **E14** | 0.3803  (35) |  |  |  |  | -0.48231  (35) |  |  |  |  |  |  | **Yield**  **E14** |  |
| **Yield**  **E15** |  |  |  |  |  |  |  |  | 0.48942  (62) |  | -0.32591  (62) |  |  | **Yield**  **E15** |
| **Yield**  **YOR** |  |  |  |  |  |  |  |  |  |  |  |  |  |  |
